# Ferrocenyl chiral bisphosphorus ligands for highly enantioselective asymmetric hydrogenation *via* noncovalent ion pair interaction†
†Electronic supplementary information (ESI) available. CCDC 1456993. For ESI and crystallographic data in CIF or other electronic format see DOI: 10.1039/c6sc01845a



**DOI:** 10.1039/c6sc01845a

**Published:** 2016-06-30

**Authors:** Caiyou Chen, Heng Wang, Zhefan Zhang, Shicheng Jin, Songwei Wen, Jianjian Ji, Lung Wa Chung, Xiu-Qin Dong, Xumu Zhang

**Affiliations:** a College of Chemistry and Molecular Sciences , Wuhan University , Wuhan , Hubei 430072 , P. R. China . Email: xumu@whu.edu.cn ; Email: xiuqindong@whu.edu.cn; b Department of Chemistry South University of Science and Technology of China , Shenzhen , 518055 , P.R. China

## Abstract

A new class of ferrocenyl chiral bisphosphorus ligand, Wudaphos, was developed, and exhibits excellent *ee* and activity (*ee* up to 99%, TON up to 20 000) for the asymmetric hydrogenation of both 2-aryl and 2-alkyl acrylic acids through ion pair noncovalent interaction under base free and mild reaction conditions. Well-known anti-inflammatory drugs such as naproxen and ibuprofen together with the intermediate for the preparation of Roche ester and some bioactive compounds were also efficiently obtained with excellent *ee*. Control experiments were conducted and revealed that the ion pair noncovalent interaction and chain length played important roles.

## Abstract 2

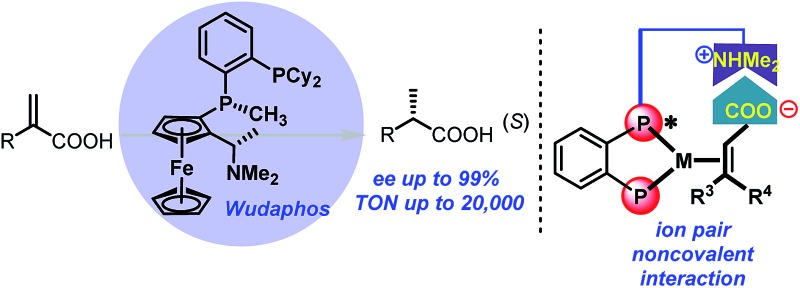
The new ferrocenyl bisphosphorus ligand, Wudaphos, was developed for highly enantioselective asymmetric hydrogenation based on noncovalent ion pair interaction.

## Introduction

Since the 1960s, the exploration of new efficient chiral phosphine ligands remains a continuous work for transition-metal catalyzed asymmetric reactions.^1^ The development of new asymmetric reactions largely relies on the exploration of new efficient chiral phosphine ligands.^2^ As a result, the research of developing more efficient and practical phosphine ligands in terms of excellent enantioselectivity and activity, good air stability, and ease of preparation remains highly desirable in asymmetric catalysis.

Attractive noncovalent interactions play a key role in the design of catalysts. Similar to enzymatic catalysis, attractive noncovalent interactions are responsible for many of the remarkable rate acceleration and stereoselectivity improvements by lowering the kinetic barrows through stabilization of the transition state and suppressing the degree of freedom in the transition state.^3^ Consequently, intensive research efforts have been directed toward the development of a new efficient catalytic system utilizing attractive noncovalent interactions.^4^ As summarized by Jacobson *et al.* (Table 1),^3^ among the three representative noncovalent interactions, the steric repulsion is highly distance dependent (1/*r* ^12^) wherein weak interaction was observed with long distance. H-Bond interaction (1/*r* ^2^) and ion pair interaction (1/*r*) are relatively less distance dependent and the ion pair interaction showed the strongest interaction. We anticipated that the strong ion pair noncovalent interaction can be utilized in the development of a new efficient catalytic system for asymmetric hydrogenation (AH), wherein the substrates can interact with the catalyst strongly, accelerating the reaction rate to a large extent. However, only a few examples have been reported concerning of the strong ion pair noncovalent interaction and the substrate scope was limited.^5^

**Table 1:** Distance dependencies of the representative noncovalent interactions

Entry	Noncovalent interaction	Energy dependence on distance
1	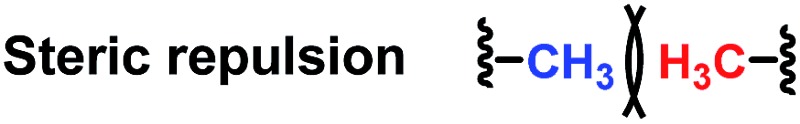	1/*r* ^12^
2	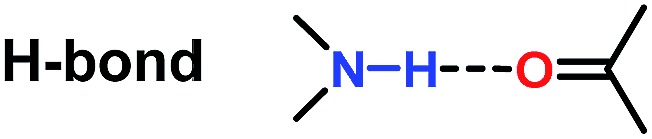	Complicated ∼1/*r* ^2^
3	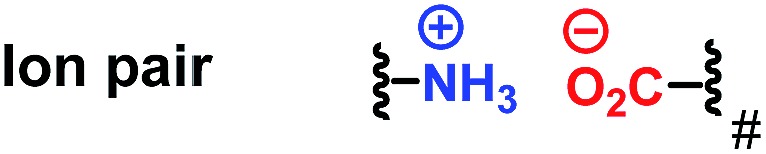	1/*r*

**Scheme 1:**
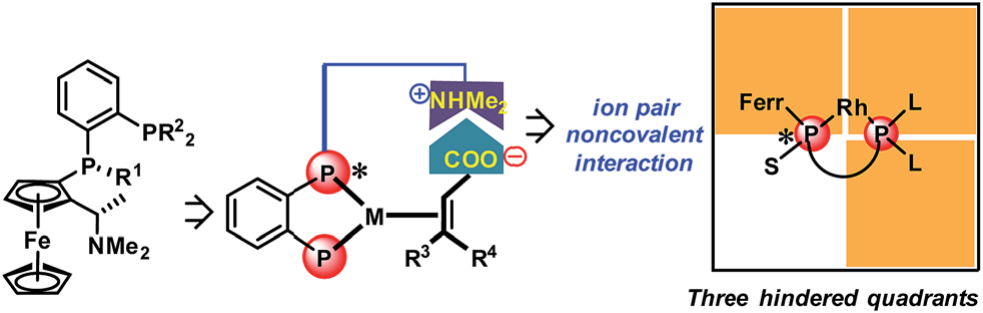
The new ferrocenyl ligands and the corresponding model of the three hindered quadrants. L = large substituent, S = small substituent, and Ferr = ferrocene.

With regard to the utilization of the strong ion pair noncovalent interaction in the development of new chiral catalysts for AH, we found that Ugi's amine is a privileged motif in which the dimethyl amine moiety can play as a proton acceptor which can interact with the acid substrates strongly through noncovalent ion pair interaction (Scheme 1). Moreover, the Ugi's amine motif can conveniently incorporate planar chirality, C-chirality and P-chirality into the catalytic system as exemplified by many efficient chiral ligands such as Josiphos,^6^ Walphos,^7^ Taniaphos,^8^ Bophoz,^9^ Mandyphos,^10^ TRAP,^11^ Trifer^5*a*^ and Chenphos.^5*b*^ Herein, we report a new class of bisphosphorus ligands incorporating the Ugi's amine motif (Scheme 1). This type of bisphosphorus ligand possesses one chiral phosphine and the chiral Ugi amine moiety. Two large substituents of the non-chiral phosphine together with a ferrocene backbone block three quadrants, and the small substituent of the chiral phosphine makes the remaining quadrant open. This three blocked quadrant model is believed to have good chiral induction.^12^ Furthermore, the dimethyl amine unit in the ligand can be a proton acceptor and thus can interact with the acid substrates through noncovalent ion pair interaction. According to the above hypothesis, this catalytic system is believed to exhibit excellent enantioselectivity and activity in the AH of unsaturated acid substrates.

**Scheme 2:**
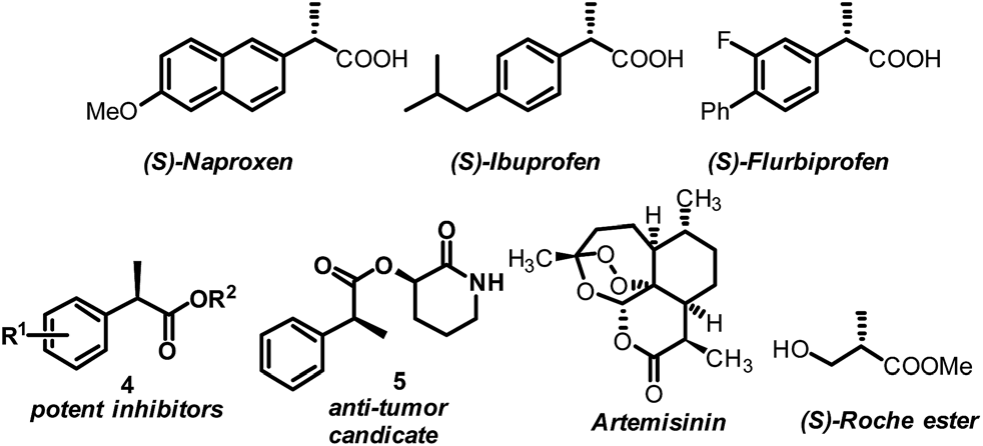
Representative drugs and chemicals featuring the α-substituted propanoic acid moiety.

**Scheme 3:**
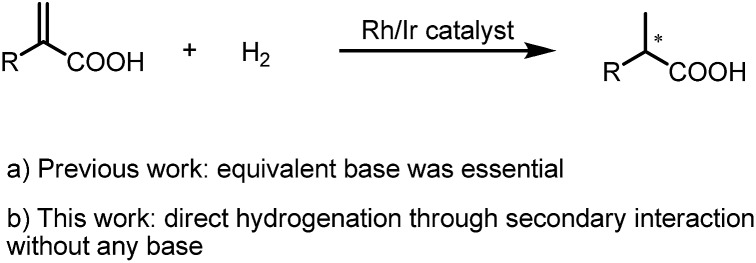
AH of 2-substituted acrylic acids.

AH of 2-substituted acrylic acids will generate chiral α-substituted propanoic acids which are important units that widely exist in pharmaceuticals and fine chemicals (Scheme 2). Good examples are the well-known non-steroid anti-inflammatory and analgesic drugs such as naproxen, ibuprofen and flurbiprofen,^13^ the esterification potent inhibitors **4** against the inflammatory phenotype of the cystic fibrosis (CF) lung disease,^14^ the bioactive natural product **5** isolated from the *Fusarium oxysporum* which shows cytotoxicity against three human cancer cell lines PC-3, PANC-1 and A549,^15^ and artemisnin^16^ which is a famous drug against *Plasmodium falciparum* malaria that won the 2015 Nobel Prize in medicine. Furthermore, α-substituted propanoic acid can also be used to prepare the synthetically important Roche ester, a well-known synthon in total synthesis.^17^ Although AH of 2-substituted acrylic acids was previously realized using chiral Ru catalysts,^18^ the reactions were limited by the generally required high pressure. Chiral Rh and Ir catalysts have also recently been reported to realize this valuable transformation.^19^ However, most of these catalytic systems need an equivalent base to neutralize the free acid.^19^ This method is non-direct and needs additional procedure to remove the base. Herein we report a ferrocenyl catalytic system that can finely utilize the free acid *via* the attractive noncovalent ion pair interaction to realize this valuable transformation in a direct and concise way without any base with *ee* (enantiomeric excess) up to 99% and TON up to 20 000 (Scheme 3).

## Results and discussion

**Scheme 4:**
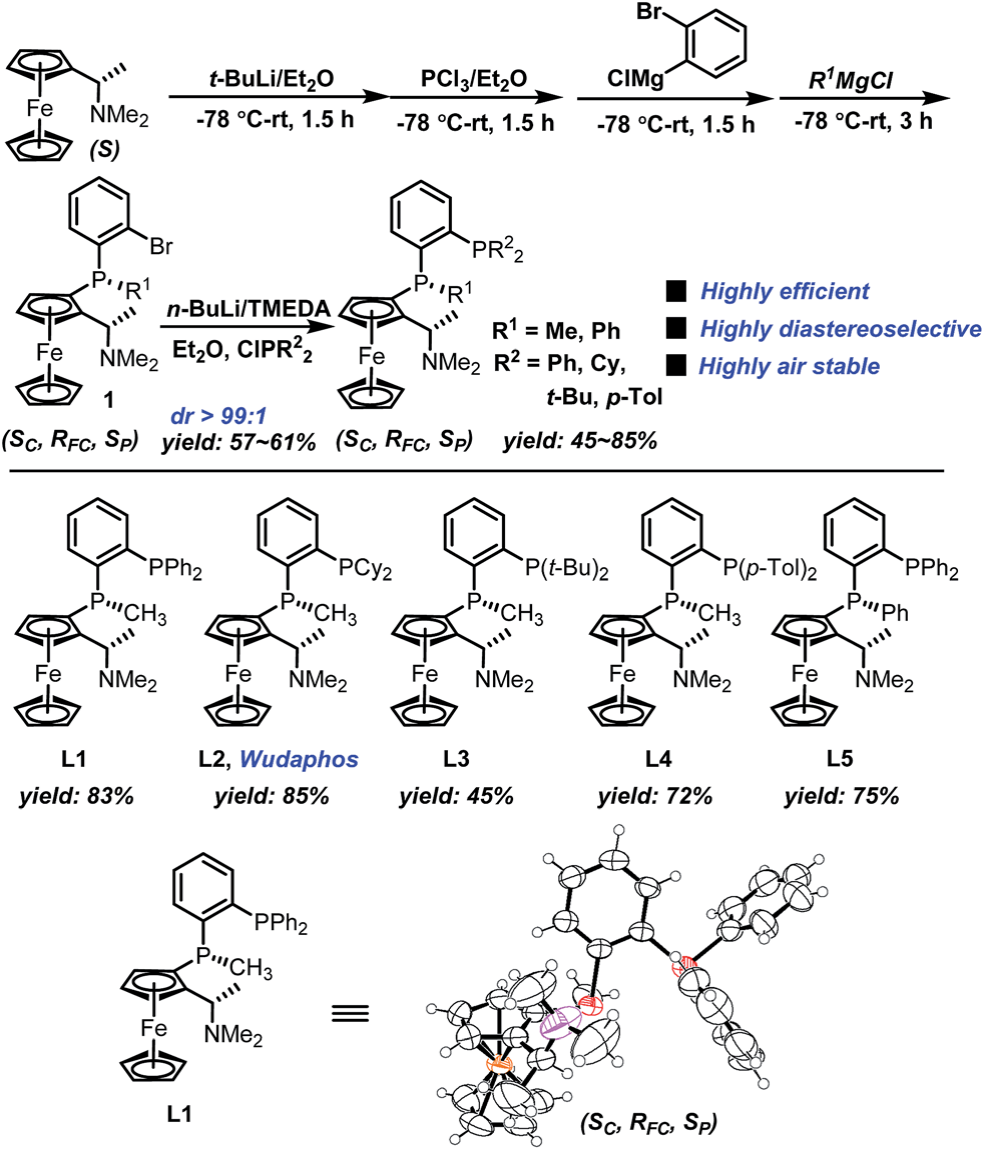
Synthesis of the ferrocenyl new bisphosphorus ligands.

The new ferrocenyl bisphosphorus ligands we report herein can be easily synthesized in two-pot with very high diastereoselectivity (dr > 99 : 1) (Scheme 4). Starting from (*S*)-Ugi amine, a one pot sequential reaction gave **1** efficiently. Importantly, compound **1** was obtained as a single diastereomer as determined by NMR which makes the synthesis to be very simple and practical. The subsequent lithiation followed by treating with different chlorophosphines afforded the desired ligands **L1–L5** in good yields. Moreover, the absolute configuration of **L1** was determined using X-ray spectrum analysis as (*S*_c_, *R*_FC_, *S*_p_).^20^ Importantly, ligands **L1–L5** are all highly air stable even when stored under air for more than one year.

With ligands **L1–L5** in hand, AH of 2-substituted acrylic acids was initiated by evaluating ligand effects using 2-phenyl acrylic acid **2a** as a model substrate (Table 2). We were pleased to find that the substrate was all smoothly converted except when using **L5** as the ligand (Table 2, entry 5) in the absence of any base. The hydrogenation results highly depended on the ligand structure. With **L1**, a good *ee* was obtained (Table 2, entry 1). Changing the phenyl group in **L1** into a cyclohexyl group (**L2**, Table 2, entry 2), the *ee* significantly increased to 98%. However, to our surprise, further changing the phenyl group into the steric bulky *t*-butyl group resulted in a very low *ee* and the product configuration was changed from (*S*) to (*R*) (**L3**, Table 2, entry 3). This is probably due to the much too big steric hindrance of the *t*-butyl group which influences the noncovalent ion pair interaction between the ligand and substrate. Further changing the phenyl group into the *p*-tolyl group resulted in a decreased *ee* (**L4**, Table 2, entry 4). Using **L5** as a ligand with a phenyl group instead of a methyl group attached to the chiral phosphine, the *ee* dropped significantly (**L5**, Table 2, entry 5). This result corresponds to the hypothesis that we made that the ligand should have three blocked quadrants and make the remaining quadrant open in order to get good enantioselectivities (Scheme 1). In terms of reactivity and enantioselectivity, **L2** was selected as the optimum ligand for further investigation. Due to the remarkable performance, herein we name **L2** Wudaphos.

**Table 2:** Ligand effect in the asymmetric hydrogenation of 2-phenyl acrylic acid^*a*^

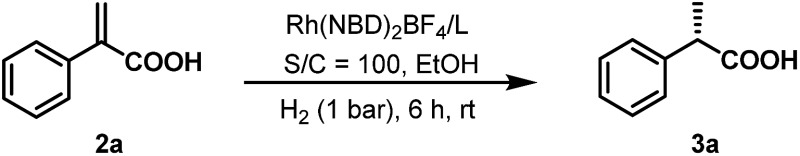				
Entry	Ligand	Conv.^*b*^%	*ee* ^*c*^%	Configuration^*d*^
1	**L1**	>99	84	(*S*)
2	**Wudaphos**	>99	98	(*S*)
3	**L3**	>99	33	(*R*)
4	**L4**	>99	74	(*S*)
5	**L5**	67	57	(*S*)

^*a*^The reaction was conducted in a 0.1 mmol scale in 1 mL of EtOH, [Rh(NBD)_2_]BF_4_ (NBD = norbornadiene) was used as metal precursor, S/C = 100, L/Rh = 1.1 : 1, temperature = rt, H_2_ pressure = 1 bar, reaction time = 6 h.

^*b*^Substrate conversion, determined using ^1^H NMR.

^*c*^Enantiomeric excess of **3a**, determined using chiral HPLC after treating **3a** with CH_2_N_2_.

^*d*^Configuration of **3a**, determined by comparing the optical rotation data with those reported in the literature.

Subsequently, the substrate scope of the AH of 2-substituted acrylic acids was investigated using the optimum ligand Wudaphos under the best reaction conditions (for the screening of the reaction conditions and the discussion of the solvent effects, see ESI†). As listed in Table 3, 2-aryl acrylic acids were efficiently hydrogenated with excellent enantioselectivities under mild reaction conditions in the absence of any base regardless of whether the substituents on the phenyl ring were electron donating (Table 3, **3a–3e**), electron withdrawing (Table 3, **3h**), or halogens (Table 3, **3f–3h**). 2-Alkyl acrylic acids were also smoothly hydrogenated with high *ee* (Table 3, **3i**, **3j**, **3m**). Thus, the intermediate for the preparation of Roche ester was conveniently obtained in high *ee* (Table 3, **3m**). Furthermore, the well-known anti-inflammatory drugs ibuprofen and naproxen were also easily obtained in excellent *ee* under mild conditions (Table 3, **3k**, **3l**). Our catalytic system shows a clear advance compared with the previous systems owing to its high enantioselectivity and base free conditions.

**Table 3:** Substrate scope using Wudaphos as the ligand^*a*^

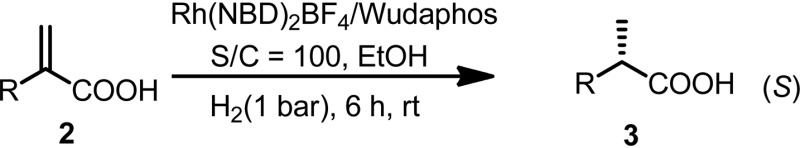
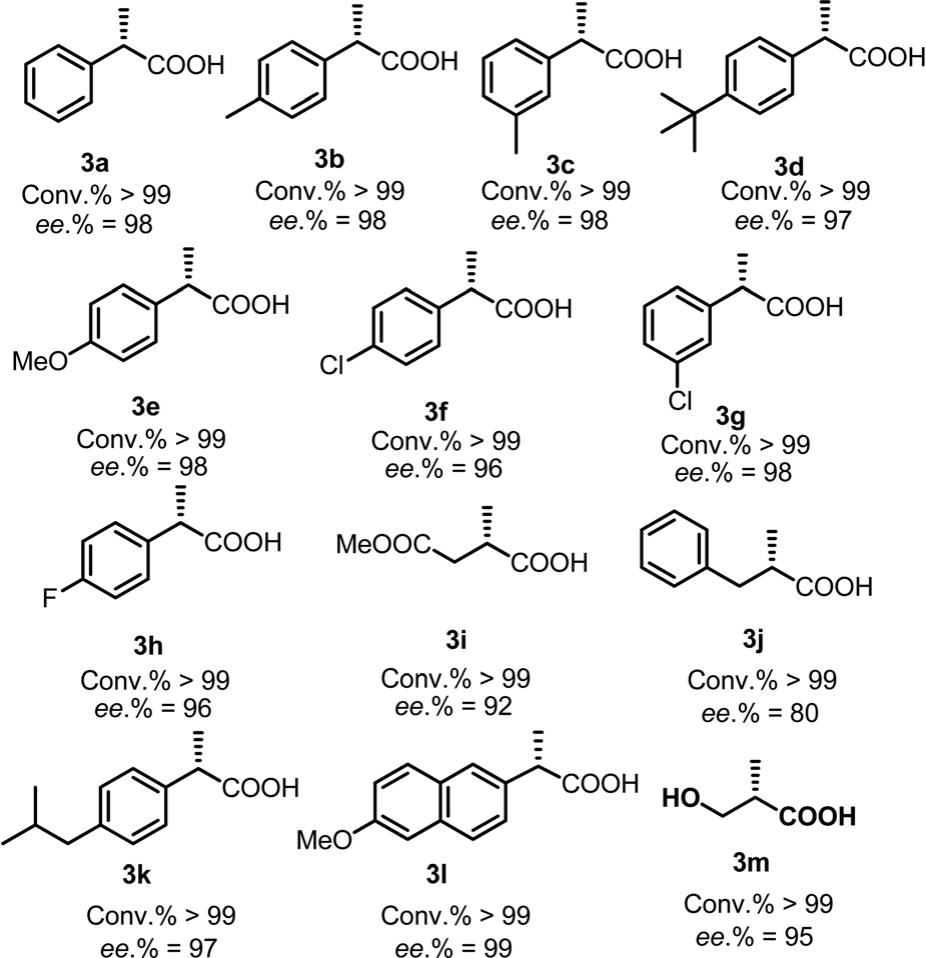

^*a*^The reaction was conducted in a 0.1 mmol scale in 1 mL of EtOH, [Rh(NBD)_2_]BF_4_ (NBD = norbornadiene) was used as the metal precursor, Wudaphos was used as the ligand, S/C = 100, L/Rh = 1.1 : 1, temperature = rt, H_2_ pressure = 1 bar, reaction time = 6 h, the configuration of all the product was determined as (*S*) by comparing the optical rotation data with those reported by the literature, the *ee* was determined *via* chiral HPLC after esterification with CH_2_N_2_, and the conversion of the substrates was determined using ^1^H NMR.

**Scheme 5:**
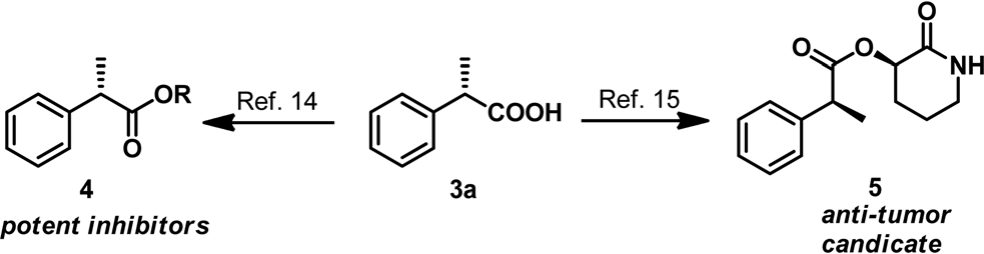
Synthesis of the potent inhibitors **4** and the bioactive natural product **5**.

The asymmetric hydrogenation of 2-substituted acrylic acids was also conducted with low catalyst loading using **2a** as a model substrate under 50 bar H_2_ atmosphere. Satisfyingly, our catalyst system showed excellent activity under very mild conditions in the absence of any base when the catalyst loading was 0.02 mol% (S/C = 5000) without any decrease of the *ee* (Table 4, entry 1). Moreover, the hydrogenation also proceeded smoothly with full conversion and high *ee* when lowering the catalyst loading to 0.01 mol% (S/C = 10 000, Table 4, entry 2) or 0.005 mol% (S/C = 20 000, Table 4, entry 3) *albeit* with a slight drop of *ee*. As shown in Scheme 5, the potent inhibitors **4** (ref. 14) and the bioactive natural product **5** (ref. 15) can be readily synthesized following literature procedures starting from the hydrogenation product **3a**.

**Table 4:** TON experiment with Wudaphos as the ligand^*a*^

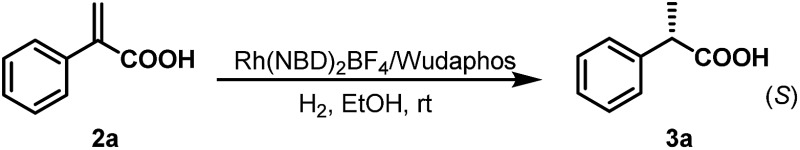				
Entry	S/C	Time (h)	Conv.^*b*^%	*ee* ^*c*^%
1	5000	6	>99	98
2	10 000	12	>99	97
**3**	**20 000**	**24**	**>99**	**96**

^*a*^The reaction was conducted in EtOH, [Rh(NBD)_2_]BF_4_ (NBD = norbornadiene) was used as the metal precursor, Wudaphos was used as the ligand, L/Rh = 1.1 : 1, temperature = rt, and H_2_ pressure = 50 bar.

^*b*^Substrate conversion, determined *via* ^1^H NMR.

^*c*^Enantiomeric excess of **3a**, determined using chiral HPLC after esterification with CH_2_N_2_.

**Scheme 6:**
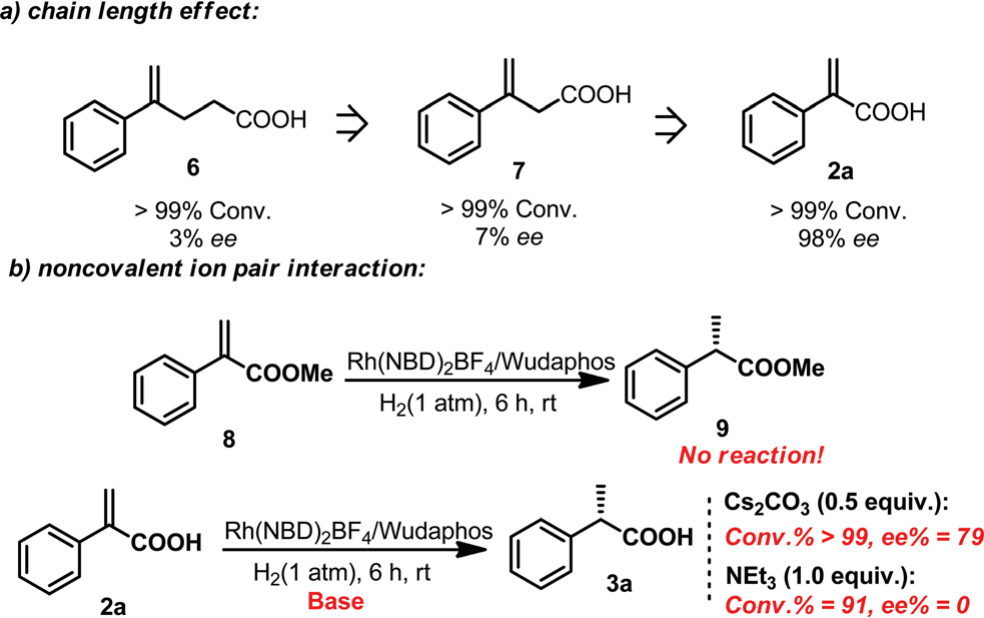
Control experiments for the investigation of the chain length effect and the ion pair noncovalent interaction effect.

In order to gain more insights into this catalytic system, several control experiments were also conducted. The effect of the chain length between the olefin and the acid moiety was first investigated. It was found that the chain length played an important role in determining the *ee*. Although the hydrogenation reactions of compounds **6**, **7** and **2a** all proceeded smoothly, the *ee* obtained in the hydrogenation of **6** and **7** was very low (Scheme 6), which indicated that the excellent enantiomeric control was based on a matched chain length. Subsequently, the effect of the ion pair noncovalent interaction was also investigated. Hydrogenation of the ester substrate **8** did not occur at all. The *ee* of the hydrogenation of **2a** also dropped evidently when adding 0.5 equivalent of Cs_2_CO_3_. Moreover, only a racemic product was observed when one equivalent amount of triethylamine was added. It is probably due to the reason that the ion pair interaction between the ligand and substrate was interrupted by the additional base. These results suggested that the ion pair noncovalent interaction between the ligand and the acid substrates is critical (Scheme 6).

**Scheme 7:**
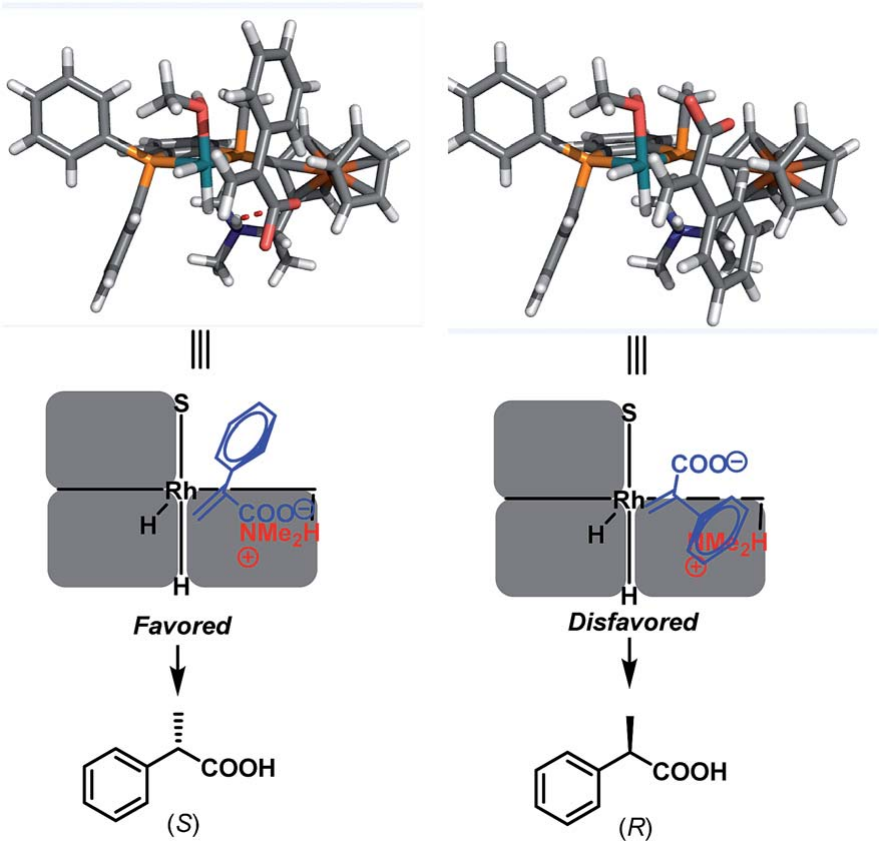
3D models and the predicted enantiomeric control.

On the basis of the observed (*S*)-enantioselectivity, the X-ray crystal structure of ligand **L1** (Scheme 1) and previous computational studies,^21^ 3D models were built to account for the important roles of the ion pair interaction and the small substituent on the phosphine ligand (Scheme 7). The favorable ionic pair interaction is present and the phenyl group on the substrate experiences less repulsion with the phosphine ligand for (*S*)-hydrogenation. In contrast, such an ionic pair interaction cannot form and the phenyl group on the substrate has larger repulsion with the ferrocenyl group for the (*R*)-hydrogenation. These 3D models also correspond to the hypothesis that we made that the good enantiomeric control of the Wudaphos is based on the three hindered quadrant model.

## Conclusions

In summary, a new class of ferrocenyl chiral bisphosphorus ligand, Wudaphos, was developed. The Wudaphos type ligands are highly air stable and exhibit excellent *ee* and activity (*ee* up to 99%, TON up to 20 000) for the asymmetric hydrogenation of both 2-aryl and 2-alkyl acrylic acids. Importantly, the hydrogenation reaction was efficiently realized through the attractive ion pair noncovalent interaction in base free and mild reaction conditions, which shows a clear advance compared with the previous catalytic system. Well-known anti-inflammatory drugs such as naproxen and ibuprofen together with the intermediate for the preparation of Roche ester and some bioactive compounds were efficiently obtained with an excellent *ee*. Control experiments were conducted and revealed the ion pair noncovalent interaction played an important role and the excellent enantiomeric control was based on the matched chain length between the olefin and the acid moiety.

## References

[cit1] (b) Comprehensive Asymmetric Catalysis, ed. E. N. Jacobsen, A. Pfaltz and H. Yamamoto, Springer, Berlin, 1999.

[cit2] (b) Comprehensive Chirality, ed. H. Yamamoto and E. Carreira, Elsevier, 2012.

[cit3] Knowles R. R., Jacobsen E. N. (2010). Proc. Natl. Acad. Sci. U. S. A..

[cit4] Sawamura M., Ito Y. (1992). Chem. Rev..

[cit5] Chen W., McCormack P. J., Mohammed K., Mbafor W., Roberts S. M., Whittall J. (2007). Angew. Chem., Int. Ed..

[cit6] Togni A., Breutel C., Schnyder A., Spindler F., Landert H., Tijani A. (1994). J. Am. Chem. Soc..

[cit7] Weissensteiner W., Sturm T., Spindler F. (2003). Adv. Synth. Catal..

[cit8] Lotz M., Polborn K., Knochel P. (2002). Angew. Chem., Int. Ed..

[cit9] Boaz N. W., Debenham S. D., Mackenzie E. B., Large S. E. (2002). Org. Lett..

[cit10] Perea A. J. J., Borner A., Knochel P. (1998). Tetrahedron Lett..

[cit11] Sawamura M., Hamashima H., Sugawara M., Kuwano N., Ito Y. (1995). Organometallics.

[cit12] Hoge G., Wu H., Kissel W. S., Pflum D. A., Greene D. J., Bao J. (2004). J. Am. Chem. Soc..

[cit13] (a) Lednicer D. and Mitscher L. A., The Organic Chemistry of Drug Synthesis, Wiley, New York, 1977, vol. 1.

[cit14] Tchilibon S., Zhang J., Yang Q.-F., Eidelman O., Kim H., Caohuy H., Jacobson K. A., Pollard B. S., Pollard H. B. (2005). Biochem. Pharmacol..

[cit15] Krishna P. R., Kumar P. V. A., Mallula V. S., Ramakrishna K. V. S. (2013). Tetrahedron.

[cit16] Tu Y., Ni M., Zhong Y., Li L., Cui S., Zhang M., Wang X., Liang X. (1981). Yaoxue Xuebao.

[cit17] Mickel S. J., Sedelmeier G. H., Niederer D., Schuerch F., Koch G., Kuesters E., Daeffler R., Osmani A., Weibel S., Schmid M. E., Hirni A., Schaer K., Gamboni R. (2004). Org. Process Res. Dev..

[cit18] Ohta T., Takaya H., Kitamura M., Nagai K., Noyori R. (1987). J. Org. Chem..

[cit19] Robin F., Mercier F., Ricard L., Mathey F., Spagnol M. (1997). Chem.–Eur. J..

[cit20] The X-ray crystal data of **L1** has been deposited with the Cambridge Crystallographic Data Centre as supplementary publication no. CCDC ; 1456993.

[cit21] Gridnev I. D. Imamoto T., ACS Catal., 2015, 5 , 2911 , , and references therein .

