# Supplementary material for: Ferrocenyl chiral bisphosphorus ligands for highly enantioselective asymmetric hydrogenation via noncovalent ion pair interaction
Source: Chem Sci. 2016 Jun 30;7(11):6669–73. doi: 10.1039/c6sc01845a (PMC5450526; doi:10.1039/c6sc01845a)
Supplement: Supplementary file 1 [file SC-007-C6SC01845A-s001.pdf]

Electronic Supplementary Material (ESI) for Chemical Science.
This journal is © The Royal Society of Chemistry 2016

Supporting Information for

Ferrocenyl Chiral Bisphosphorus Ligands for Highly Enantioseletive Asymmetric Hydrogenation via the Noncovalent Ion Pair Interaction

Caiyou Chen, [a] Heng Wang,[a] Zhefan Zhang,[a] Shicheng Jin, [a] Songwei Wen,[a] Jianjian Ji,[a] Lung Wa Chung,[b] Xiu-Qin Dong*[a], and Xumu Zhang*[a, b]

[a] *College of Chemistry and Molecular Sciences, Wuhan University, Wuhan, Hubei, 430072, P. R. China*
[b] *Department of Chemisty, South University of Science and Technology of China, Shenzhen, 518055, P.R. China.*

Contents

General Remarks..2
Synthesis ligands L1-L5..2
Data obtained under other reaction conditions 7
General procedure for the hydrogenation of 2-aryl and 2-alkyl acrylic acids 9
Characterization data of compounds 3a-3m 9
NMR Spectra 16
HPLC and GC results of compounds 3a-3m...40
References..53

General Remarks

All reactions were performed in the argon-filled glovebox or under nitrogen using standard Schlenk techniques, unless otherwise noted. Solvents were dried with standard procedures and degassed with N_2. Column chromatography was performed using 200~400 mesh silica gel. Thin layer chromatography (TLC) was performed on EM reagents 0.25 mm silica 60-F plates. ^{1}H, ^{13}C, ^{31}P NMR spectrum were recorded on Bruker-400, with $CDCl_3$ as the solvent and tetramethylsilane (TMS) as the internal standard. Chemical shifts were reported in ppm, upfield to TMS (0.00 ppm) for and relative to $CDCl_3$ (7.26 ppm, 77.3 ppm) for ^{1}H NMR and ^{13}C NMR. HPLC analysis was conducted on an Agilent 1260 Series instrument. GC analysis was carried out on SHIMADZU Lab Solution using achiral capillary columns. High resolution mass spectrum was obtained on Thermo LTQ XL Orbitrap. Unless otherwise noted, all reagents and solvents were purchased from commercial suppliers and used without further purification. Substrates **2a** and **2j** were commercially available. Substrates **2b-2i**[1], **2k**[2], **2l**[3], **2m**[4]**,** **4**[5] and **5**[6] were prepared according to the literature procedures.

Synthesis ligands L1-L5

1. Synthesis of intermediate 1a

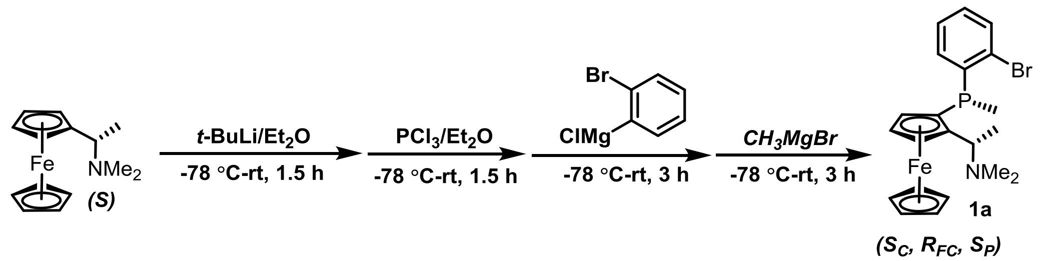

To an oven dried schlenk flask was added (*S*)-ugi's amine (10 mmol, 2.5715 g) and 20 mL of dry Et_2O under N_2 atmosphere. The resulting solution was cooled to -78 ℃ and *t*-BuLi (11 mmol, 1.5 M in pentane, 7.6 mL) was added carefully and dropwise. After the addition, the solution was allowed to warm to room temperature (rt) and stirred for 1.5 h. The schlenk flask was cooed to -78 ℃ and PCl_3 (10 mmol, 1.0 mL) was added in one portion. The suspension was allowed to warm to rt and

stirred for 1.5 h. The schlenk flask was cooled to -78 ℃ and (2-bromophenyl)magnesium chloride (11 mmol, prepared by treating 1-bromo-2-iodobenzene with *i*-PrMgCl in an 1:1 molar ratio under -40 ℃ for 1 h in Et_2O) was added dropwise and the resulting suspension was allowed to warm to rt and stirred for 1.5 h. The schlenk flask was cooled to -78 ℃ again and CH_3MgCl (11 mmol, 3 M in Et_2O, 3.7 mL) was added dropwise. The resulting suspension was allowed to warm to rt and stirred for 3 h. Water (20 mL) was added into the schlenk flask and the solution was stirred for 10 min. The organic phase was seprated and the water phase was extracted by ethyl acetate (50 mL X 3). The organic phases were combined , dried and concentrated under reduced pressure. The residue was purified by column chromatography to give the desired product **1a** as a yellow solid (2.79 g, 61% yield). ^{1}H NMR (400 MHz, $CDCl_3$): δ 7.46-7.42 (m, 1H), 7.04-7.00 (m, 2H), 6.91 (d, *J* = 7.6 Hz, 1H), 4.42-4.37 (m, 3H), 4.16 (s, 5H), 4.12-4.06 (m, 1H), 1.69 (s, 6H), 1.56 (d, *J* = 4.4 Hz, 3H), 1.22 (d, *J* = 6.4 Hz, 3H) ppm; ^{13}C NMR (101 MHz, $CDCl_3$) δ 144.85 (d, *J* = 13.5 Hz), 132.93, 131.75 (d, *J* = 2.9 Hz), 128.71, 127.72, 127.40, 126.42, 76.50 (d, *J* = 7.9 Hz), 70.10, 69.90 (d, *J* = 3.8 Hz), 69.73, 69.49 (d, *J* = 4.8 Hz), 68.24, 57.11 (d, *J* = 7.1 Hz), 39.07, 11.21 (d, *J* = 10.5 Hz) ppm; ^{31}P NMR (162 MHz, $CDCl_3$): δ -40.787 ppm. HRMS (ESI) calculated for $C_{21}H_{26}BrFeNP^+$ [M + H^+]: 458.0330; found: 458.0330.

2. Synthesis of intermediate 1b

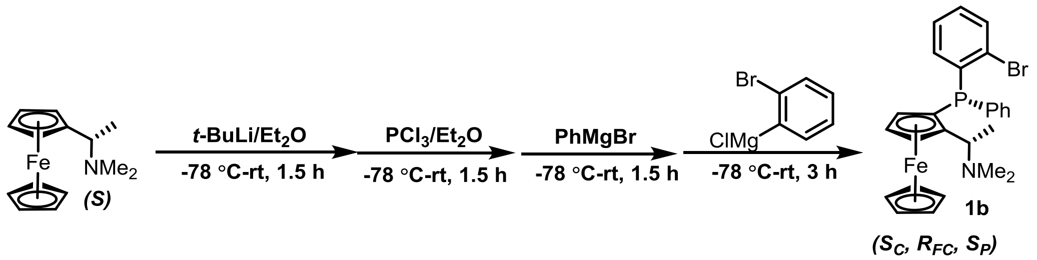

To an oven dried schlenk flask was added (*S*)-ugi's amine (10 mmol, 2.5715 g) and 20 mL of dry Et_2O under N_2 atmosphere. The resulting solution was cooled to -78 ℃ and *t*-BuLi (11 mmol, 1.5 M in pentane, 7.6 mL) was added carefully and dropwise. After the addition, the solution was allowed to warm to room temperature (rt) and stirred for 1.5 h. The schlenk flask was cooed to -78 ℃ and PCl_3 (10 mmol,

1.0 mL) was added in one portion. The suspension was allowed to warm to rt and stirred for 1.5 h. The schlenk flask was cooled to -78 ℃ and PhMgCl (11 mmol, 2 M, 5.5 mL) was added dropwise. The schlenk flask was cooled to -78 ℃ and (2-bromophenyl)magnesium chloride (11 mmol, prepared by treating 1-bromo-2-iodobenzene with *i*-PrMgCl in an 1:1 molar ratio under -40 ℃ for 1 h in Et_2O) was added dropwise and the resulting suspension was allowed to warm to rt and stirred for 1.5 h. The resulting suspension was allowed to warm to rt and stirred for 3 h. Water (20 mL) was added into the schlenk flask and the solution was stirred for 10 min. The organic phase was seprated and the water phase was extracted by ethyl acetate (50 mL X 3). The organic phases were combined , dried and concentrated under reduced pressure. The residue was purified by column chromatography to give the desired product **1a** as a yellow solid (2.96 g, 57% yield). ^{1}H NMR (400 MHz, $CDCl_3$) δ 7.70 (s, 1H), 7.35 (d, J = 6.9 Hz, 1H), 7.27 – 7.07 (m, 7H), 4.45 (s, 1H), 4.37 (s, 1H), 4.09 (s, 2H), 4.03 (s, 5H), 1.91 (s, 6H), 1.35 (d, J = 6.7 Hz, 3H), ppm; ^{13}C NMR (101 MHz, $CDCl_3$): δ 141.87 (d, J = 11.4 Hz), 141.12 (d, J = 12.0 Hz), 137.84, 133.08 (d, J = 3.7 Hz), 132.33 (d, J = 18.8 Hz), 131.89 (d, J = 39.1 Hz), 130.61, 127.64 (d, J = 5.8 Hz), 127.16 (d, J = 18.2 Hz), 97.73 (d, J = 25.0 Hz), 75.86 (d, J = 15.1 Hz), 71.36 (d, J = 2.7 Hz), 69.95, 69.63 (d, J = 4.2 Hz), 69.16, 56.91 (d, J = 8.3 Hz), 39.90, 11.31 ppm; ^{31}P NMR (162 MHz, $CDCl_3$): δ -22.86 (s) ppm. HRMS (ESI) calculated for $C_{26}H_{28}BrFeNP^+$ [M + H$^+$]: 520.0487; found: 520.0487.

3. **General procedure for the synthesis of ligands L1-L5**

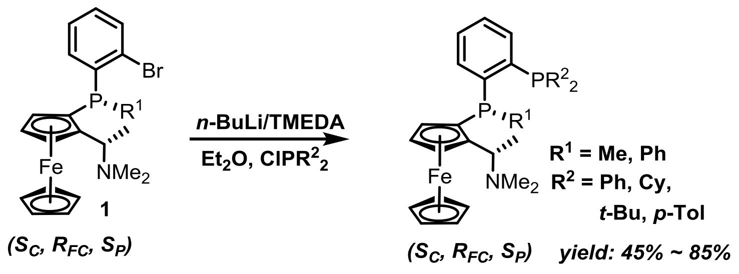

To an oven dried schlenk flask was added intermediate **1a** (3.0 mmol, 1.3746 g) or **1b** (3.0 mmol, 1.56 g), TMEDA (N^1, N^1, N^2, N^2-tetramethylethane-1, 2-diamine, 3.3 mmol, 383.5 mg) and 20 mL of dry Et_2O under N_2 atmosphere. The resulting solution was cooled to -78 ℃ and *n*-BuLi (3.3 mmol, 2.3 M, 1.4 mL) was added dropwise.

The resulting solution was stirred at -78 ℃ for 1 h. The corresponding phosphine chloride was added dropwise at -78 ℃ and the resulting solution was allowed to warm to rt and stirred for 3 h. Water (20 mL) was added into the schlenk flask and the solution was stirred for 10 min. The organic phase was seprated and the water phase was extracted by ethyl acetate (30 mL X 3). The organic phases were combined , dried and concentrated under reduced pressure. The residue was purified by column chromatography to give the desired ligands **L1-L5** as yellow solid.

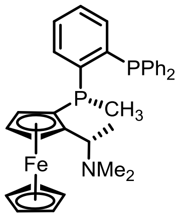

(S_C, R_{FC}, S_P) L1

L1: Yellow solid, 1.40 g, 83% yield. $[\alpha]_D^{20}$ = +1.3 (c 0.4, $CHCl_3$). ^1H NMR (400 MHz, $CDCl_3$): δ 7.40 – 7.21 (m, 11H), 7.17 – 7.02 (m, 3H), 6.98 – 6.89 (m, 1H), 4.393 – 4.343 (m, 3H), 4.12 (s, 5H), 4.07 (dd, *J* = 6.7, 2.8 Hz, 1H), 1.66 (s, 6H), 1.30 (d, *J* = 4.5 Hz, 3H), 1.26 (d, *J* = 6.3 Hz, 3H) ppm; ^{13}C NMR (101 MHz, $CDCl_3$): δ 134.14 (d, *J* = 19.5 Hz), 133.65 (d, *J* = 19.2 Hz), 133.33 (d, *J* = 6.6 Hz), 131.42 (d, *J* = 9.7 Hz), 128.38 (d, *J* = 3.0 Hz), 128.35 (d, *J* = 16.3 Hz), 128.22, 127.46, 77.52 (d, *J* = 52.7 Hz), 69.71, 69.59 (d, *J* = 4.9 Hz), 68.06 (d, *J* = 2.6 Hz), 56.85, 39.68, 12.70 (dd, *J* = 10.2, 1.8 Hz) ppm; ^{31}P NMR (162 MHz, $CDCl_3$): δ -16.79 (d, *J* = 164.5 Hz), -50.76 (d, *J* = 164.1 Hz) ppm. HRMS (ESI) calculated for $C_{33}H_{36}FeNP_2^+$ [M + H^+]: 564.1667; found: 564.1667.

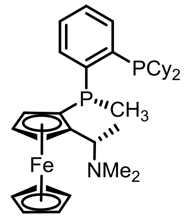

(S_C, R_{FC}, S_P) L2

L2: Yellow solid, 1.47 g, 85% yield. $[\alpha]_D^{20}$ = -53.0 (c 0.4, $CHCl_3$). ^1H NMR (400 MHz, $CDCl_3$): δ 7.43 – 7.35 (m, 1H), 7.13 (td, *J* = 7.4, 1.2 Hz, 1H), 7.04 (t, *J* = 7.5 Hz, 1H), 6.91 (dt, *J* = 6.5, 2.7 Hz, 1H), 4.43 (s, 1H), 4.40 (t, *J* = 2.3 Hz, 1H), 4.30 (s, 1H), 4.15 (s, 5H), 3.98 (dd, *J* = 6.7, 3.2 Hz, 1H), 1.77 (s, 6H), 1.64 (d, *J* = 4.2 Hz, 4H), 1.32 (d, *J* = 6.7 Hz, 3H), 1.27 – 2.06 (m, 10H) ppm; ^{13}C NMR (101 MHz, $CDCl_3$): δ 153.23 (dd, *J* = 31.9, 14.4 Hz), 137.77 (dd, *J* = 26.9, 14.9 Hz), 131.73, 131.14 (d, *J* = 8.9 Hz), 127.85, 126.43, 97.48 (d, *J* = 25.3 Hz), 78.08 (dd, *J* = 14.3, 12.0 Hz), 70.22 (dd, *J* = 4.6, 2.6 Hz), 69.68, 68.34, 60.44, 56.77 (d, *J* = 8.4 Hz), 40.31, 36.26 (dd, *J* = 13.8, 3.0 Hz), 33.97 (dd, *J* = 12.5, 5.1 Hz), 30.82 – 30.48 (m), 30.28 (d,

J = 11.7 Hz), 28.72 (d, *J* = 5.6 Hz), 27.40 (dd, *J* = 7.5, 2.8 Hz), 27.34 (d, *J* = 41.4 Hz), 27.25, 26.49 (d, *J* = 7.2 Hz), 21.11, 14.24, 13.49 (dd, *J* = 10.1, 5.9 Hz), 11.81 ppm; ^{31}P NMR (162 MHz, $CDCl_3$): δ -13.73 (d, *J* = 147.0 Hz), -48.37 (d, *J* = 147.1 Hz) ppm. HRMS (ESI) calculated for $C_{33}H_{48}FeNP_2^+$ [M + H^+]: 576.2606; found: 576.2606.

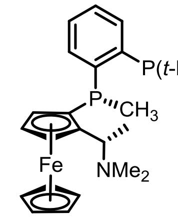

***(SC, RFC, SP)* L3**

L3: Yellow solid, 707 mg, 45% yield. $[\alpha]_D^{20}$ = +34.1 (c 0.4, $CHCl_3$). ^{1}H NMR (400 MHz, $CDCl_3$): δ 7.34 (td, *J* = 7.7, 1.7 Hz, 2H), 7.19 – 7.15 (m, 2H), 4.35 (dd, *J* = 8.6, 1.4 Hz, 2H), 4.30 – 4.20 (m, 4H), 4.17 (dd, *J* = 2.2, 1.0 Hz, 1H), 4.14 (t, *J* = 4.6 Hz, 1H), 4.08 (dd, *J* = 6.7, 2.3 Hz, 1H), 1.68 (s, 6H), 1.58 (d, *J* = 4.5 Hz, 3H), 1.20 (dd, *J* = 14.0, 11.2 Hz, 21H) ppm; ^{13}C NMR (101 MHz, $CDCl_3$) δ 143.40 (d, *J* = 10.7 Hz), 134.99 (d, *J* = 21.1 Hz), 131.47 (d, *J* = 19.0 Hz), 127.40 (d, *J* = 7.1 Hz), 127.33, 77.69 (d, *J* = 8.3 Hz), 74.09 (d, *J* = 12.9 Hz), 73.29 (d, *J* = 10.8 Hz), 72.74 (dd, *J* = 3.0, 1.5 Hz), 72.45 – 72.25 (m), 71.54, 71.31 (d, *J* = 3.7 Hz), 70.82 (d, *J* = 5.6 Hz), 70.09, 69.72, 57.02 (d, *J* = 6.5 Hz), 38.68, 32.75 (dd, *J* = 20.4, 6.7 Hz), 30.84 (dd, *J* = 13.1, 10.4 Hz), 13.16 (dd, *J* = 9.3, 1.6 Hz) ppm; ^{31}P NMR (162 MHz, $CDCl_3$): δ 26.96 (s), -44.92 (s) ppm. HRMS (ESI) calculated for $C_{29}H_{44}FeNP_2^+$ [M + H^+]: 524.2293; found: 524.2293.

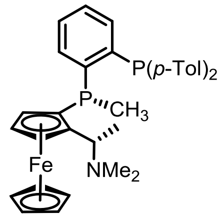

(S_C, R_{FC}, S_P) L4

L4: Yellow solid, 1.28 g, 72% yield. $[\alpha]_D^{20}$ = +25.5 (c 0.2, $CHCl_3$). ^{1}H NMR (400 MHz, $CDCl_3$): δ 7.24 – 7.01 (m, 11H), 6.952 – 6.917 (m, 1H), 4.43 – 4.33 (m, 3H), 4.13 (s, 3H), 4.05 (dd, *J* = 4.5, 2.2 Hz, 1H), 2.35 (d, *J* = 8.4 Hz, 6H), 1.67 (s, 6H), 1.39 – 1.13 (m, 7H) ppm; ^{13}C NMR (101 MHz, $CDCl_3$): δ 138.57, 138.33, 135.09 (dd, *J* = 11.5, 8.7 Hz), 134.71 (dd, *J* = 9.9, 2.9 Hz), 134.34 (d, *J* = 19.7 Hz), 133.91 (d, *J* = 19.4 Hz), 133.44 (d, *J* = 6.4 Hz), 131.62 (dd, *J* = 7.7, 2.4 Hz), 129.45 (t, *J* = 7.0 Hz), 128.41, 127.70, 78.13 (dd, *J* = 23.5, 11.6 Hz), 70.69, 70.40, 69.98, 69.83 (dd, *J* = 4.8, 1.8 Hz), 68.41, 57.17 (d, *J* = 7.6 Hz), 40.03, 21.60 (d, *J* = 4.5 Hz), 13.04 (dd, *J* = 10.4, 1.7 Hz) ppm; ^{31}P NMR (162 MHz, $CDCl_3$): δ -18.51 (d, *J*

= 165.2 Hz), -51.64 (d, *J* = 164.9 Hz) ppm. HRMS (ESI) calculated for $C_{35}H_{40}FeNP_2^+$ [M + H$^+$]: 592.1980; found: 592.1980.

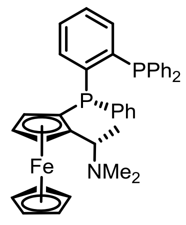

(S_C, R_{FC}, S_P) L5

L5: Yellow solid, 1.41 g, 75% yield. $[\alpha]_D^{20}$ = +220.0 (c 0.4, $CHCl_3$). ^{1}H NMR (400 MHz, $CDCl_3$): δ 7.41 (td, *J* = 7.5, 1.7 Hz, 1H), 7.25-7.26 (m, 2H), 7.21 – 7.02 (m, 4H), 7.01 – 6.90 (m, 1H), 6.76-6.80 (m, 1H), 4.39 (s, 1H), 4.29 – 4.19 (m, 1H), 3.95 (s, 2H), 3.80 (s, 1H), 1.76 (s, 3H), 1.30 – 1.15 (m, 2H) ppm; ^{13}C NMR (101 MHz, $CDCl_3$): δ 140.35, 139.99 (d, *J* = 14.9 Hz), 138.92 (dd, *J* = 13.0, 8.0 Hz), 137.78, 136.69 (dd, *J* = 11.4, 2.8 Hz), 135.84 (d, *J* = 20.9 Hz), 134.45 (d, *J* = 4.8 Hz), 133.65 (dd, *J* = 29.4, 19.2 Hz), 132.95 (d, *J* = 8.4 Hz), 128.43, 128.37, 128.13, 128.09, 128.06, 128.02, 127.97, 127.90, 127.82, 72.74 (dd, *J* = 5.1, 2.4 Hz), 69.92, 69.82 (d, *J* = 3.9 Hz), 68.39, 57.33 (d, *J* = 7.8 Hz), 39.54, 29.97 ppm; ^{31}P NMR (162 MHz, $CDCl_3$): δ -17.08 (d, *J* = 168.2 Hz), -30.35 (d, *J* = 165.1 Hz) ppm. HRMS (ESI) calculated for $C_{38}H_{38}FeNP_2^+$ [M + H$^+$]: 626.1823; found: 626.1823.

Data obtained under other reaction conditions

Table 1. Screening of solvents using 2a as the standard substrate [a]

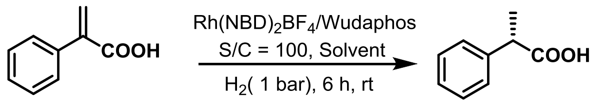

Entry	Solvent	Conv.% [b]	EE% [c]
1	MeOH	> 99	97
2	***EtOH***	***> 99***	***98***
3	*i*-PrOH	> 99	97
4	CF_3CH_2OH	> 99	95
5	CH_2Cl_2	56	89
6	$ClCH_2CH_2Cl$	12	34
7	CH_3Cl	0	-
8	THF	70	3
9	CH_3CN	58	95
10	EA	14	42
11	Toluene	15	47

[a]The reaction was conducted in 0.1 mmol scale in 1 mL of solvents, $[Rh(NBD)_2]BF_4$ (NBD = norbornadiene) was used as metal precursor, Wudaphos was used as the ligand, S/C = 100, L/Rh = 1.1:1, temperature = rt, H_2 pressure = 10 bar, reaction time = 6 h. [b]Substrate conversion, determined by ^1H NMR. [c]Enantiomeric excess of **3a**, determined by chiral HPLC after treating **3a** with CH_2N_2.

Table 2. Screening of metal precursors and H_2 pressure using 2a as the standard substrate [a]

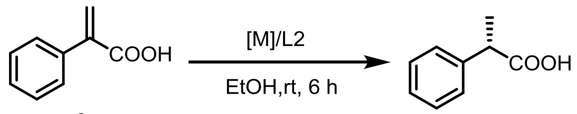

Entry	Metal precursor	P (bar)[b]	Conversion (%)[c]	Ee (%)[d]
**1**	**$[Rh(NBD)_2]BF_4$**	**10**	**>99**	**98**
2	$[Rh(COD)_2]BF_4$	10	>99	97
3	$[Rh(COD)Cl]_2$	10	>99	97
4	$[Ir(COD)Cl]_2$	10	66	-5
5	$RuCl_2Ph$	10	>99	16
6	$[Rh(NBD)_2]BF_4$	20	>99	98
**7**	**$[Rh(NBD)_2]BF_4$**	**1**	**>99**	**98**

[a]The reaction was conducted in 0.1 mmol scale in 1 mL of sovents EtOH, Wudaphos was used as the ligand, L/M = 1.1:1, S/C = 100, temperature = rt, reaction time = 6 h. [c]Substrate conversion, determined by ^1H NMR. [d]Enantiomeric excess of **3a**, determined by chiral HPLC after treating the **3a** with CH_2N_2.

Using Wudaphos as the optimized ligand, solvent effects were investigated using **2a** as the standard substrate. As depicted in Table 1, solvent has a significant influence on the reactivity and enantioselectivity for the hydrogenation of **2a**. It was found that only the polar protic alcohol solvents give excellent results in high activity and enantioselectivity (Table 1, entries 1-4). For the other polar aprotic or nonpolar aprotic solvents, low activity and eantioselectivity were observed (Table1, entries 5-11). These results suggested that there probably existed the hydrogen bond effect among the protic solvent, the acid substrate and the ligand (Scheme 1). And the interaction of the substrate and ligand could be adjusted by the hydrogen bond effect to give the high efficiency of the catalyst; otherwise, unsatisfactory results were obtained. Subsequently, metal precursors and H_2 pressure were also screened in order to determine the optimized reaction conditions. The results were listed in table 2.

$[Rh(NBD)_2]BF_4$ was selected as the optimized precursor for the slightly better *ee* obtained compared with the other two rhodium precursors. We further found that H_2 pressure had little effect on both the conversion and *ee* when the reaction was conducted using 1 mol% catalyst loading. The reaction proceeded smoothly with 1 bar of H_2 pressure with excellent *ee*. As a result, conditions in entry 7, Table 2 was selected as the optimized for the further investigations.

General procedure for the hydrogenation of 2-aryl and 2-alkyl acrylic acids

In an argon-filled glove box, $[Rh(NBD)_2]BF_4$ (0.01 mmol) and Wudaphos (0.011 mmol) were dissolved in EtOH (1 mL) and stirred for 30 min. 0.1 mL of the resulting solution was transferred by syringe into the vials charged with different substrates (0.1 mmol for each). Additional EtOH was added to bring the total reaction volume to 1 mL. The vials were subsequently transferred into an autoclave which was charged with hydrogen (1 bar). The reaction was then stirred at rt for 6 h. The hydrogen gas was released slowly and carefully in a well-ventilated hood. The solution was passed through a short column of silica gel (eluent: EtOAc) to remove the metal complex and concentrated to give compounds **3**. The ee values of compounds **3** were then determined by HPLC analysis on a chiral stationary phase after treating the products by using CH_2N_2.

Characterization data of compounds 3a-3m

According to the above mentioned procedure, compounds **3a-3m** can be obtained. Characterization data are as follows.

(*S*)-2-phenylpropanoic acid, **3a**

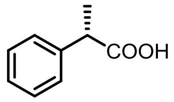

3a

>99% conv., 98% ee, white solid,; $[\alpha]_D^{20}$ = +69.2 (c 0.322, $CHCl_3$) [$[\alpha]_D^{20}$ = +71.5 (c 2.0, $CHCl_3$) for optically pure S-isomer][8]; ^1H NMR (500 MHz, $CDCl_3$): δ = 7.31-7.25 (m, 5H), 3.73 (q, J = 7.2 Hz, 1H), 1.50 (d, J = 7.2 Hz, 3H) ppm; ^{13}C NMR (126 MHz, $CDCl_3$): δ 180.85, 140.11, 128.95, 127.89, 127.64, 45.69, 18.41. The enantiomeric excess of **3a** was determined by chiral HPLC analysis on Chiralpak OJ-H column after esterification with CH_2N_2. Conditions: hexane/isopropanol = 99 :1, flow rate = 1.0 mL/min, uv-vis detection at λ = 205 nm, t_R = 15.0 min (major), 18.9 min (minor).

(*S*)-2-(p-tolyl)propanoic acid, **3b**

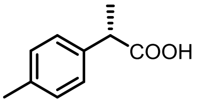

3b

>99% conv., 98% ee, colorless oil; $[\alpha]_D^{20}$ = +57.3 (c 0.26, $CHCl_3$) [$[\alpha]_D^{20}$ = +66.4 (c 0.71, $CHCl_3$) for 100% ee, *S*-isomer][9]; ^1H NMR (500 MHz, $CDCl_3$): δ = 8.98 (s, br, 1H), 7.19 (d, J = 8.4 Hz, 2H), 7.12 (d, J = 8.0 Hz, 2H), 3.68 (q, J = 6.8 Hz, 1H), 2.31 (s, 3H), 1.47 (d, J = 7.2 Hz, 3H) ppm; ^{13}C NMR (126 MHz, $CDCl_3$): δ 180.95, 137.27, 137.22, 129.62, 127.74, 45.29, 21.32, 18.43 ppm. The enantiomeric excess of **3b** was determined by chiral HPLC analysis on Chiralpak OJ-H column after esterification with CH_2N_2. Conditions: hexane/isopropanol = 99 :1, flow rate = 1.0 mL/min, uv-vis detection at λ = 220 nm, t_R = 14.3 min (minor), 16.9 min (major).

(*S*)-2-(m-tolyl)propanoic acid, **3c**

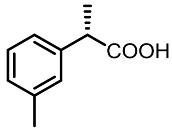

3c

>99% conv., 98% ee, colorless oil; $[\alpha]_D^{20}$ = +61.3 (c 0.256, $CHCl_3$) [$[\alpha]_D^{24}$ = +64.0 (c 1.0, $CHCl_3$) for 92% ee, *S*-isomer][12]. ^1H NMR (500 MHz, $CDCl_3$); δ: 7.21-7.18 (m, 1H); 7.11-7.06 (m, 3H); 3.68 (ddd, $J_1 = J_2 - J_3$ = 7.5 Hz, 1H); 2.33 (s, 3H); 1.48 (d, J=7.5 Hz, 3H) ppm; ^{13}C NMR (126 MHz, $CDCl_3$): δ 180.93, 140.12, 138.60, 128.84, 128.61, 128.39, 124.89, 45.66, 21.68, 18.42 ppm. The enantiomeric

excess of **3c** was determined by chiral HPLC analysis on Chiralpak OJ-H column after esterification with CH_2N_2. Conditions: hexane/isopropanol = 99 :1, flow rate = 1.0 mL/min, uv-vis detection at λ = 220 nm, t_R = 11.7 min (major), 17.1 min (minor).

(*S*)-2-(4-(tert-butyl)phenyl)propanoic acid, **3d**

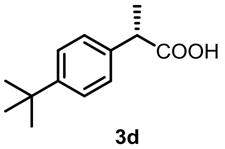

3d

>99% conv., 97% ee, white solid; $[\alpha]_D^{20}$ = +35.2 (c 0.352, $CHCl_3$) [$[\alpha]_D^{25}$= +129.0 (c 0.25, $CHCl_3$) for 90% ee, *S*-isomer][13]; ^{1}H NMR (500 MHz, DMSO): 12.27 (br, 1H), 7.34-7.32 (m, 2H), 7.25-7.23 (m, 2H), 3.79 (q, *J* = 7.0 Hz, 1H), 1.48 (d, *J* = 7.0 Hz, 3H), 1.30 (s, 9H) ppm; ^{13}C NMR (126 MHz, $CDCl_3$) δ 180.98, 150.46, 137.05, 127.52, 125.84, 45.20, 34.73, 31.61, 18.37 ppm. The enantiomeric excess of **3d** was determined by chiral HPLC analysis on Chiralpak OJ-H column after esterification with CH_2N_2. Conditions: hexane/isopropanol = 99 :1, flow rate = 1.0 mL/min, uv-vis detection at λ = 220 nm, t_R = 7.2 min (minor), 8.7 min (major).

(*S*)-2-(4-methoxyphenyl)propanoic acid, **3e**

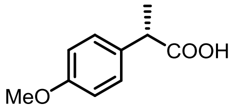

3e

>99% conv., 98% ee, white solid; $[\alpha]_D^{20}$ = +45.1 (c 0.264, $CHCl_3$) [$[\alpha]_D^{20}$ = -76.3 (c 1.02, $CHCl_3$) for 95% ee, *R*-isomer][10]; ^{1}H NMR (500 MHz, $CDCl_3$): δ = 7.82 (s, br, 1H), 7.22 (d, *J* = 8.8 Hz, 2H), 6.84 (d, *J* = 8.4 Hz, 2H), 3.77 (s, 3H), 3.66 (q, *J* = 5.6 Hz, 1H), 1.46 (d, *J* = 6.8 Hz, 3H) ppm; ^{13}C NMR (126 MHz, $CDCl_3$): δ 181.05, 159.08, 132.36, 128.88, 114.31, 55.52, 44.88, 18.48 ppm. The enantiomeric excess of **3e** was determined by chiral HPLC analysis on Chiralpak OJ-H column after esterification with CH_2N_2. Conditions: hexane/isopropanol = 99 :1, flow rate = 1.0 mL/min, uv-vis detection at λ = 220 nm, t_R = 27.3 min (major), 30.1 min (minor).

(*S*)-2-(4-chlorophenyl)propanoic acid, **3f**

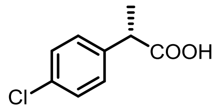

3f

>99% conv., 96% ee, white solid; $[\alpha]_D^{20}$ = +45.3 (c 0.316, $CHCl_3$) [$[\alpha]_D^{20}$ = +66.3 (c 0.9, $CHCl_3$) for 98% ee, *S*-isomer][9]; ^{1}H NMR (500 MHz, $CDCl_3$): δ = 7.28-7.24 (m, 2H), 7.01-6.98 (m, 2H), 3.69 (q, *J* = 7.2 Hz, 1H), 1.47 (d, *J* = 7.2 Hz, 3H) ppm; ^{13}C NMR (126 MHz, $CDCl_3$): δ 180.81, 163.35, 161.39, 135.83, 135.80, 129.48, 129.42, 115.85, 115.68, 44.99, 18.52 ppm. The enantiomeric excess of **3f** was determined by chiral HPLC analysis on Chiralpak OJ-H column after esterification with CH_2N_2. Conditions: hexane/isopropanol = 99 :1, flow rate = 1.0 mL/min, uv-vis detection at λ = 210 nm, t_R = 10.7 min (minor), 11.7 min (major).

(*S*)-2-(3-chlorophenyl)propanoic acid, **3g**

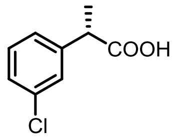

3g

>99% conv., 98% ee, white solid; $[\alpha]_D^{20}$ = +49.8 (c 0.312, $CHCl_3$) [$[\alpha]_D^{20}$= +53.9 (c 1.2, $CHCl_3$) for 97% ee, *S*-isomer][9]; ^{1}H NMR (500 MHz, $CDCl_3$): δ = 7.29-7.17 (m, 4H), 3.67 (q, *J* = 7.2 Hz, 1H), 1.46 (d, *J* = 7.2 Hz, 3H) ppm; ^{13}C NMR (126 MHz, $CDCl_3$): δ 180.21, 142.11, 134.72, 130.15, 128.14, 127.82, 126.16, 45.53, 18.34 ppm. The enantiomeric excess of **3g** was determined by chiral HPLC analysis on Chiralpak OJ-H column after esterification with CH_2N_2. Conditions: hexane/isopropanol = 99 :1, flow rate = 1.0 mL/min, uv-vis detection at λ = 210 nm, t_R = 8.8 min (major), 9.3 min (minor).

(*S*)-2-(4-fluorophenyl)propanoic acid, **3h**

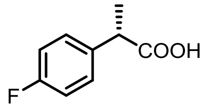

3h

>99% conv., 96% ee, white solid; $[\alpha]_D^{20}$ = +40.7 (c 0.256, $CHCl_3$) [$[\alpha]_D^{20}$= +53.5 (c 0.62, $CHCl_3$) for 98% ee, *S*-isomer][9]; ^{1}H NMR (500 MHz, $CDCl_3$): δ = 8.27 (s, br, 1H), 7.28-7.25 (m, 2H), 7.01-6.97 (m, 2H), 3.70 (q, *J* = 7.2 Hz, 1H), 1.47 (d, *J* = 7.2

Hz, 3H); ^{13}C NMR (126 MHz, $CDCl_3$): δ 180.75, 162.36 (d, *J* = 245.8 Hz), 135.85 (d, *J* = 3.0 Hz), 129.44 (d, *J* = 8.0 Hz), 115.75 (d, *J* = 21.4 Hz), 45.00, 18.52 ppm. The enantiomeric excess of **3h** was determined by chiral HPLC analysis on Chiralpak OJ-H column after esterification with CH_2N_2. Conditions: hexane/isopropanol = 99 :1, flow rate = 1.0 mL/min, uv-vis detection at λ = 220 nm, t_R = 10.8 min (major), 11.5 min (minor).

(*S*)-4-methoxy-2-methyl-4-oxobutanoic acid, **3i**

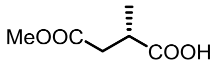

3i

>99% conv., 92% ee, colorless oil; $[\alpha]_D^{20}$ = -8.3 (c 0.202, $CHCl_3$) [$[\alpha]_D^{20}$= +9.0 (c 0.9, $CHCl_3$) for 97% ee, *R*-isomer][7]; ^{1}H NMR (400 MHz, $CDCl_3$) 9.36 (brs, 1H), 3.70 (s, 3H, CH_3), 2.96 (sextet, *J* = 7.2 Hz, 1H), 2.74 (dd, *J* = 16.4 and 8.0 Hz, 1H), 2.43 (dd, *J* = 16.4 and 6.0 Hz, 1H), 1.26 (d, *J* = 7.2 Hz, 3H) ppm; ^{13}C NMR (101 MHz, $CDCl_3$): δ 181.77, 172.51, 52.12, 37.29, 35.90, 17.09 ppm. The enantiomeric excess of **3i** was determined by chiral GC analysis on Chiral β-dex225 column after esterification with CH_2N_2. Conditions: oven temperature = 250 °C, column temperature = 80-220 °C, programming rate = 5 °C/min, detector temperature = 260 °C, N_2 flow rate = 1.0 mL/min, t_R = 13.6 min (major), 14.0 min (minor).

(S)-2-methyl-3-phenylpropanoic acid, **3j**

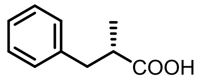

3j

>99% conv., 80% ee, colorless oil; $[\alpha]_D^{20}$ = +20.1 (*c* 0.358, $CHCl_3$) [$[\alpha]_D^{25}$ = -27.0 (*c* 1.00, CHCl3) for 98% ee, *R*-isomer] [7]; ^{1}H NMR (500 MHz, $CDCl_3$): δ = 9.47 (s, br, 1H), 7.33-7.21 (m, 5H), 3.12 (dd, *J* = 13.2, 6.0 Hz, 1H), 2.82-2.78 (m, 1H), 2.72-2.68 (m, 1H), 1.21 (d, *J* = 6.8 Hz, 3H) ppm; ^{13}C NMR (126 MHz, $CDCl_3$): δ 182.73, 139.38, 129.29, 128.69, 126.68, 41.61, 39.61, 16.75 ppm. The enantiomeric excess of **3j** was determined by chiral HPLC analysis on Chiralpak OJ-H column after esterification with CH_2N_2. Conditions: hexane/isopropanol = 99 : 1, flow rate = 1.0 mL/min, uv-vis detection at λ = 205 nm, t_R = 9.1 min (minor), 9.9 min (major).

(*S*)-2-(4-isobutylphenyl)propanoic acid, **3k**

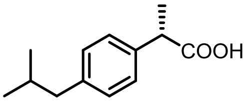
3k

>99% conv., 97% ee, white solid; $[\alpha]_D^{20}$ = +49.8 (*c* 0.368, $CHCl_3$) [$[\alpha]_D^{20}$ = -45.4 (*c* 1.00, $CHCl_3$) for 82% ee, *R*-isomer][11]; ^{1}H NMR (500 MHz, $CDCl_3$): δ = 7.21 (d, *J* = 8.0 Hz, 2H), 7.09 (d, *J* = 8.0 Hz, 2H), 3.70 (q, *J* = 7.2 Hz, 1H), 2.44 (d, *J* = 7.2 Hz, 2H), 1.89-1.78 (heptet, *J* = 6.8 Hz, 1H), 1.49 (d, *J* = 7.2 Hz, 3H), 0.89 (d, *J* = 6.8 Hz, 6H) ppm; ^{13}C NMR (126 MHz, $CDCl_3$): δ 180.77, 140.77, 137.15, 129.37, 127.30, 45.06, 30.16, 22.40, 18.16 ppm. The enantiomeric excess of **3k** was determined by chiral HPLC analysis on Chiralpak OJ-H column after esterification with CH_2N_2. Conditions: hexane/isopropanol = 99 : 1, flow rate = 1.0 mL/min, uv-vis detection at λ = 210 nm, t_R = 7.2 min (major), 7.9 min (minor).

(*S*)-2-(6-methoxynaphthalen-2-yl)propanoic acid, **3l**

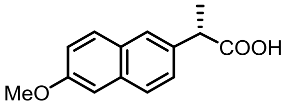
3l

>99% conv., 99% ee, white solid; $[\alpha]_D^{20}$ = +49.5 (*c* 0.41, $CHCl_3$) [$[\alpha]_D^{20}$ = +65.0 (*c* 1.0, $CHCl_3$) for optically pure *S*-isomer][7]; ^{1}H NMR (400 MHz, $CDCl_3$): δ = 7.70 (s, 1H), 7.67 (s, 2H), 7.40 (dd, *J* = 8.4, 1.6 Hz, 1H), 7.12 (dd, *J* = 8.8, 2.4 Hz, 1H), 7.09 (d, *J* = 2.0 Hz, 1H), 3.90 (s, 3H), 3.86 (q, *J* = 6.8 Hz, 1H), 1.57 (d, *J* = 7.2 Hz, 3H) ppm; ^{13}C NMR (101 MHz, $CDCl_3$): δ 181.15, 157.93, 135.12, 134.06, 129.57, 129.13, 127.49, 126.46, 126.41, 119.31, 105.81, 55.56, 45.54, 18.38 ppm. The enantiomeric excess of **3l** was determined by chiral HPLC analysis on Chiralpak OJ-H column after esterification with CH_2N_2. Conditions: hexane/isopropanol = 90:10, flow rate = 1.0 mL/min, uv-vis detection at λ = 210 nm, t_R = 25.3min (minor), 26.2 min (major).

(S)-3-hydroxy-2-methylpropanoic acid, **3m**

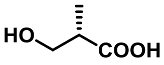
3m

>99% conv., 95% ee, colorless oil; $[\alpha]_D^{20}$ = +10.1 (c 0.186, $CHCl_3$) [$[\alpha]_D^{20}$ = + 12.72 (*c* 12.5, EtOH) for optically pure *S*-isomer][14]; ^1H NMR (400 MHz, $CDCl_3$) δ = 4.16-3.95 (m, 2H), 2.59-2.51 (m, 1H), 1.18 (d, J = 7.2 Hz, 3H). The enantiomeric excess of **3i** was determined by chiral GC analysis on Chiral CB column after esterification with CH_2N_2. Conditions: oven temerature = 230 °C, column temperature = 90-190 °C (rate = 5 °C/min), detector temperature = 240 °C, N_2 flow rate = 1.0 mL/min, t_R = 6.4 min (major), 6.6 min (minor).

NMR Spectra

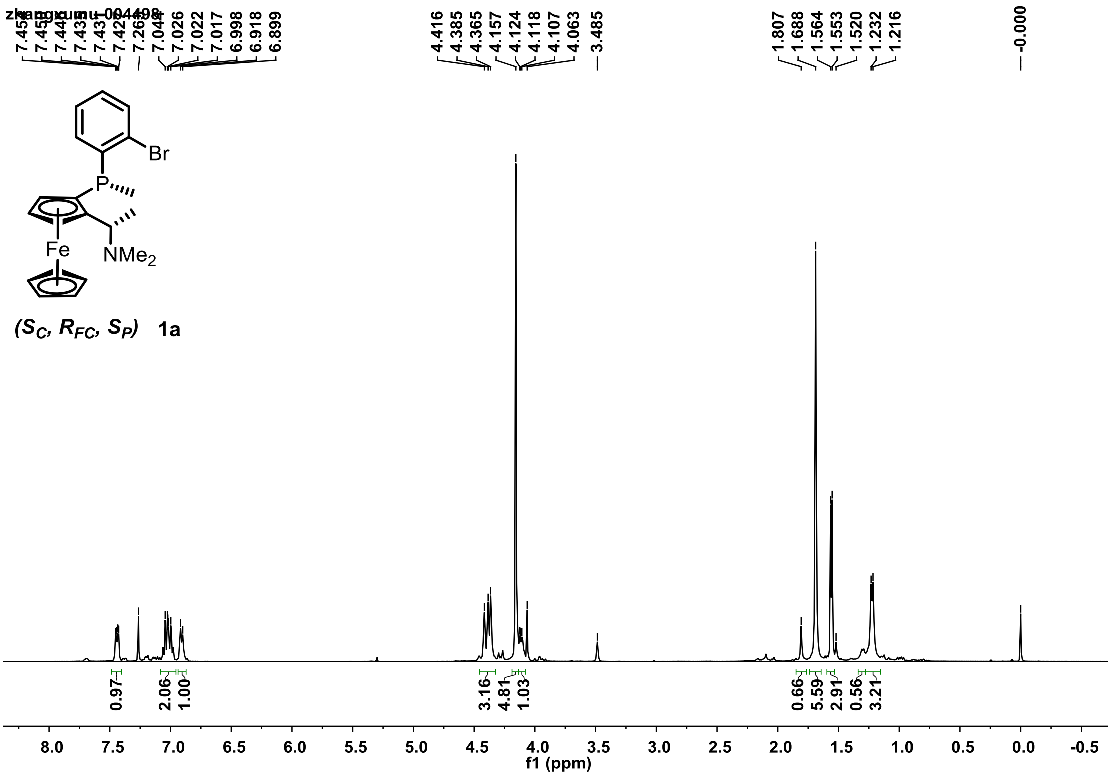
zhangxumu-004498
7.453
7.263
7.044
7.026
7.022
7.017
6.998
6.918
6.899
4.416
4.385
4.365
4.157
4.124
4.118
4.107
4.063
3.485
1.807
1.688
1.564
1.553
1.520
1.232
1.216
-0.000
Br
P
Fe
NMe2
(SC, RFC, SP) 1a
0.97
2.06
1.00
3.16
4.81
1.03
0.66
5.59
2.91
0.56
3.21
8.0 7.5 7.0 6.5 6.0 5.5 5.0 4.5 4.0 3.5 3.0 2.5 2.0 1.5 1.0 0.5 0.0 -0.5
f1 (ppm)

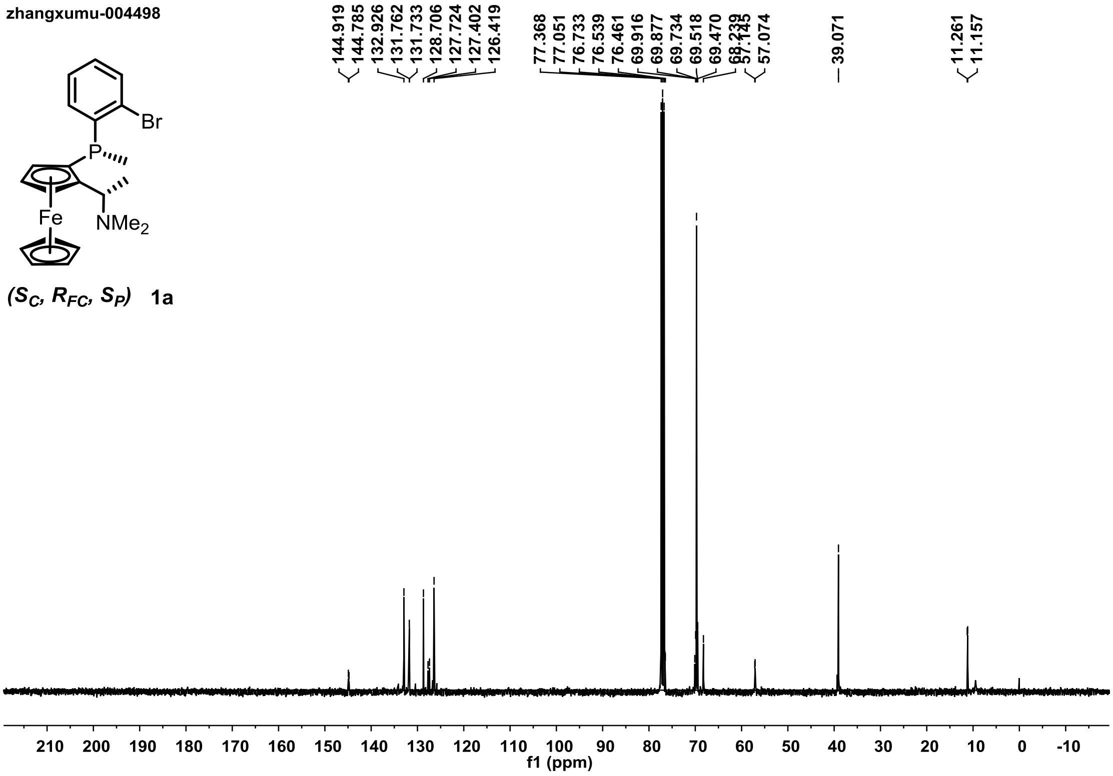
zhangxumu-004498
144.919
144.785
132.926
131.762
131.733
128.706
127.724
127.402
126.419
77.368
77.051
76.733
76.539
76.461
69.916
69.877
69.734
69.518
69.470
57.074
39.071
11.261
11.157
Br
P
Fe
NMe2
(SC, RFC, SP) 1a
210 200 190 180 170 160 150 140 130 120 110 100 90 80 70 60 50 40 30 20 10 0 -10
f1 (ppm)

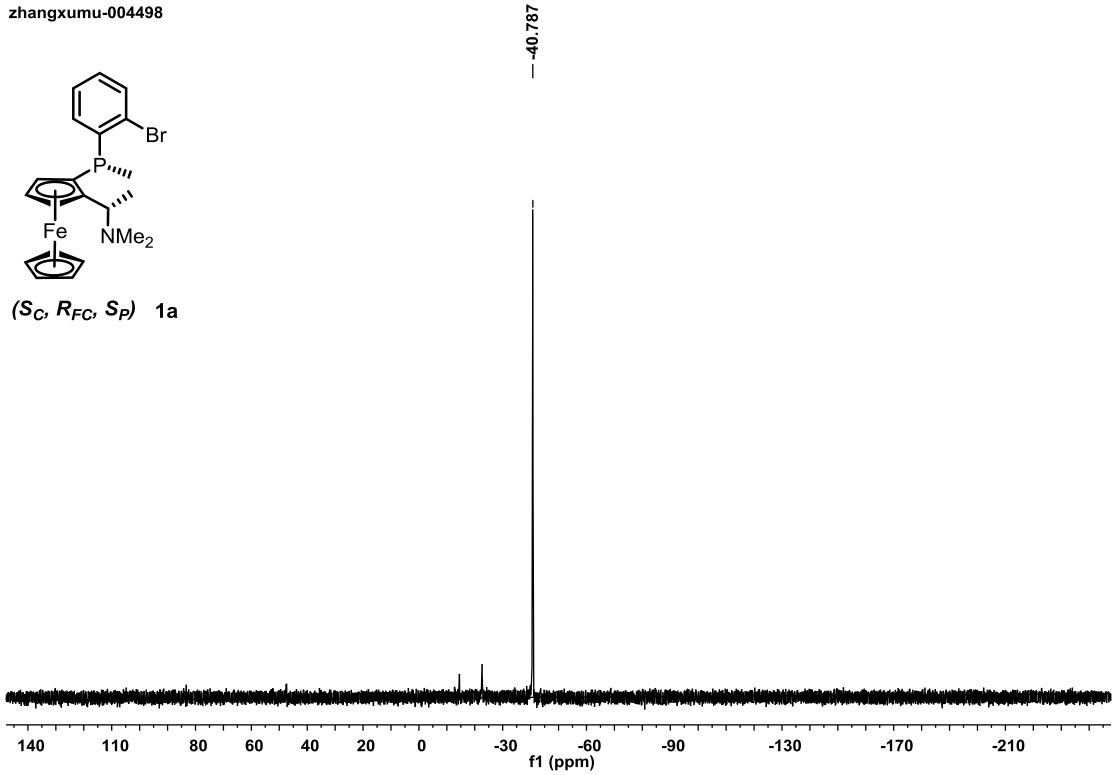
zhangxumu-004498
-40.787
Br
P
Fe
NMe2
(SC, RFC, SP) 1a
140 110 80 60 40 20 0 -30 -60 -90 -130 -170 -210
f1 (ppm)

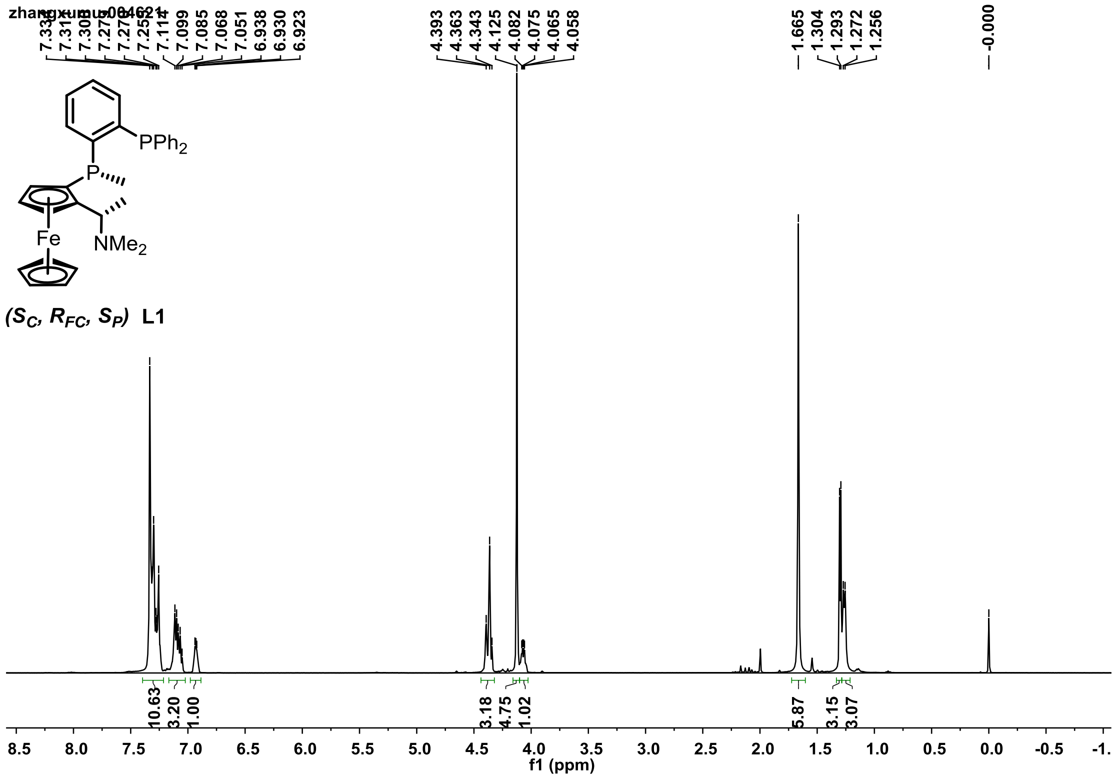
zhangxumu-004621
7.114 7.099 7.085 7.068 7.051 6.938 6.930 6.923
4.393 4.363 4.343 4.125 4.082 4.075 4.065 4.058
1.665 1.304 1.293 1.272 1.256
-0.000
PPh2
P
Fe
NMe2
(SC, RFC, SP) L1
10.63 3.20 1.00
3.18 4.75 1.02
5.87 3.15 3.07
8.5 8.0 7.5 7.0 6.5 6.0 5.5 5.0 4.5 4.0 3.5 3.0 2.5 2.0 1.5 1.0 0.5 0.0 -0.5 -1.
f1 (ppm)

zhangxumu-004621

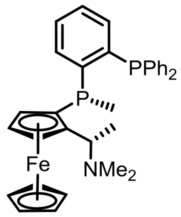

(*S*$_C$, *R*$_{FC}$, *S*$_P$) **L1**

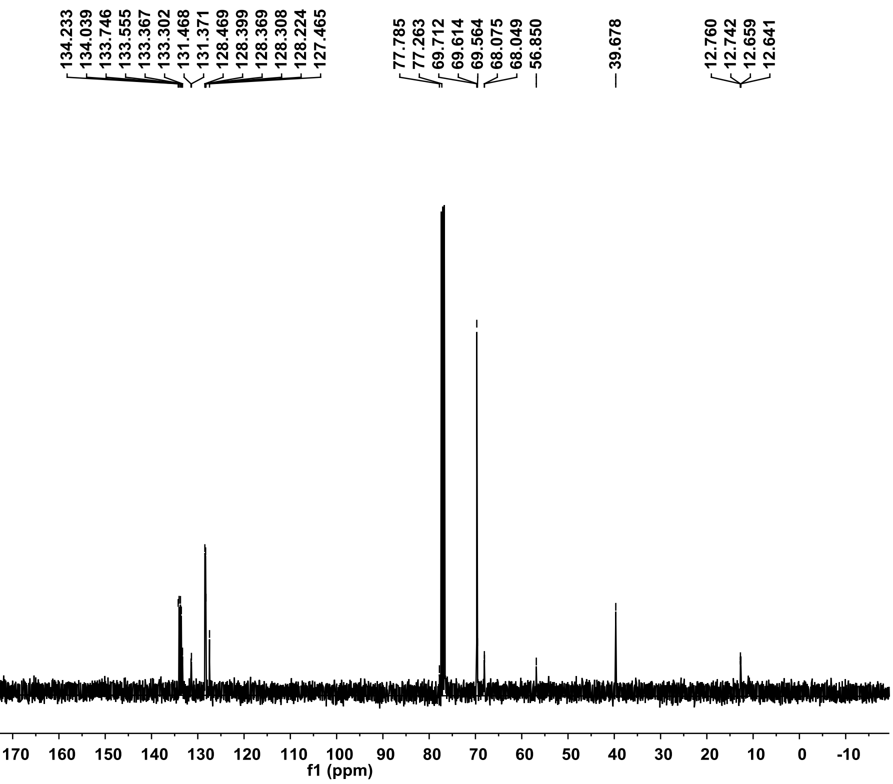

zhangxumu-004612

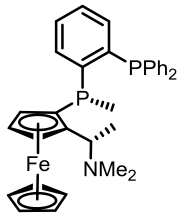

(*S*$_C$, *R*$_{FC}$, *S*$_P$) **L1**

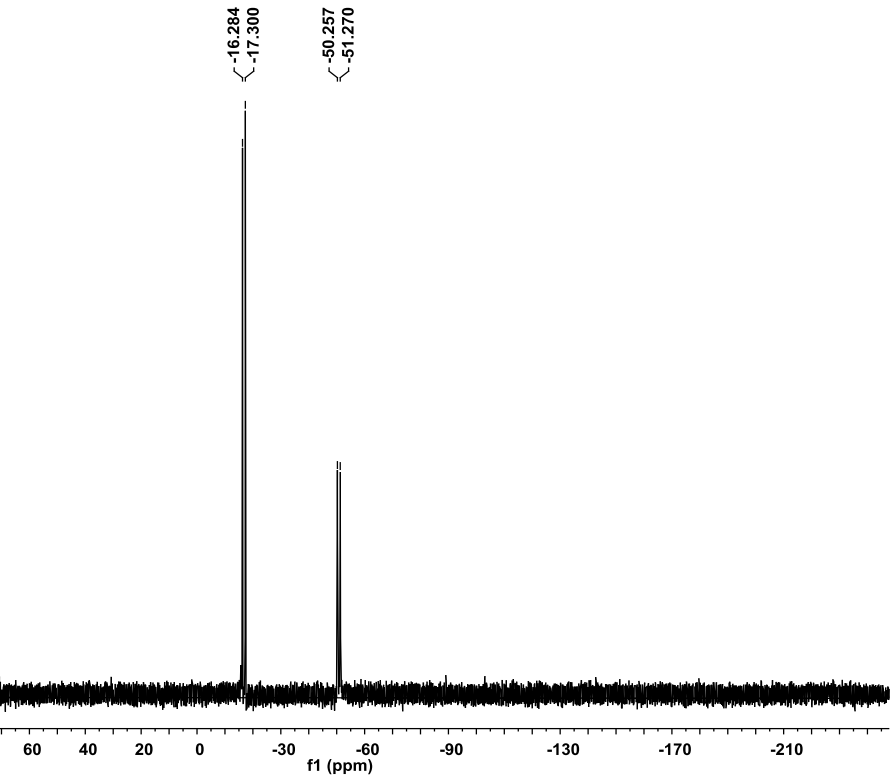

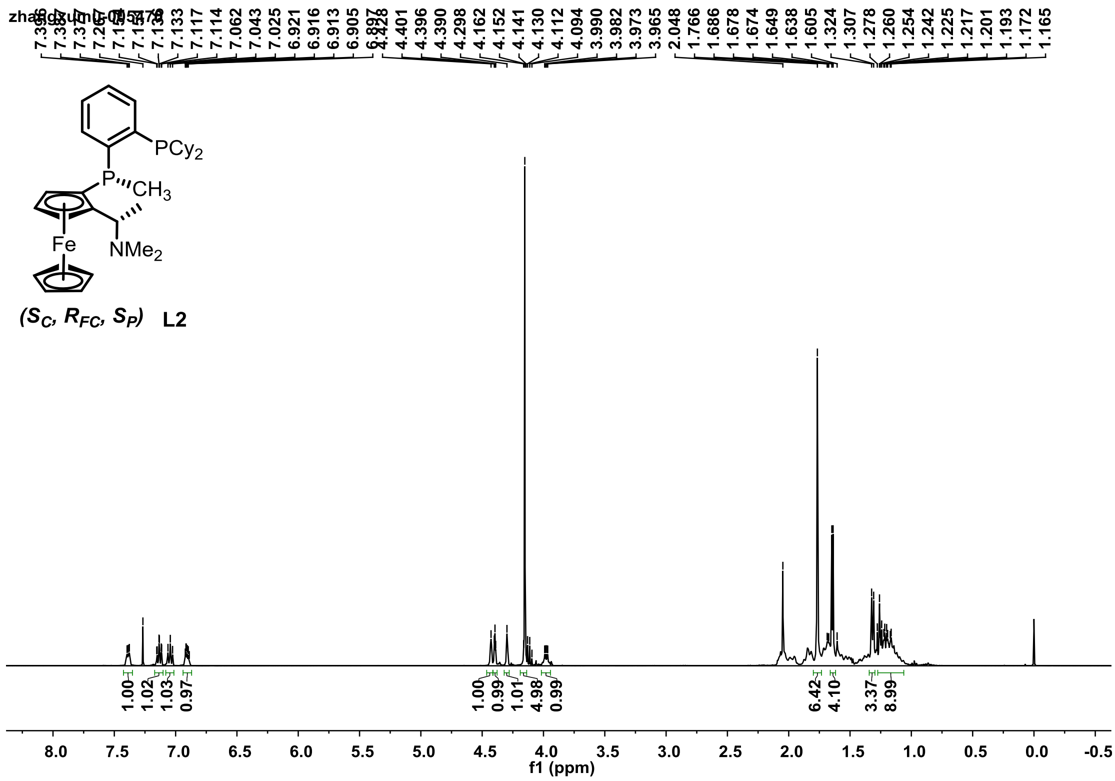
zhangxumu-005470
PCy2
P
CH3
Fe
NMe2
(SC, RFC, SP) L2
f1 (ppm)

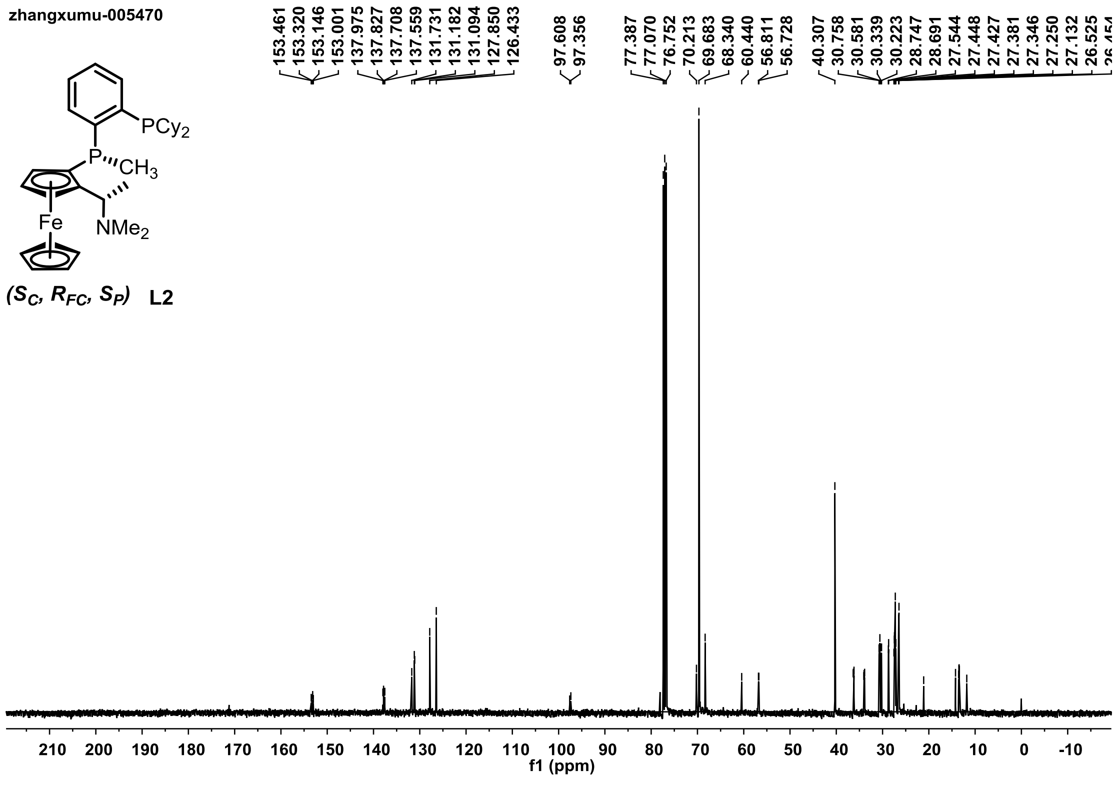
zhangxumu-005470
PCy2
P
CH3
Fe
NMe2
(SC, RFC, SP) L2
f1 (ppm)

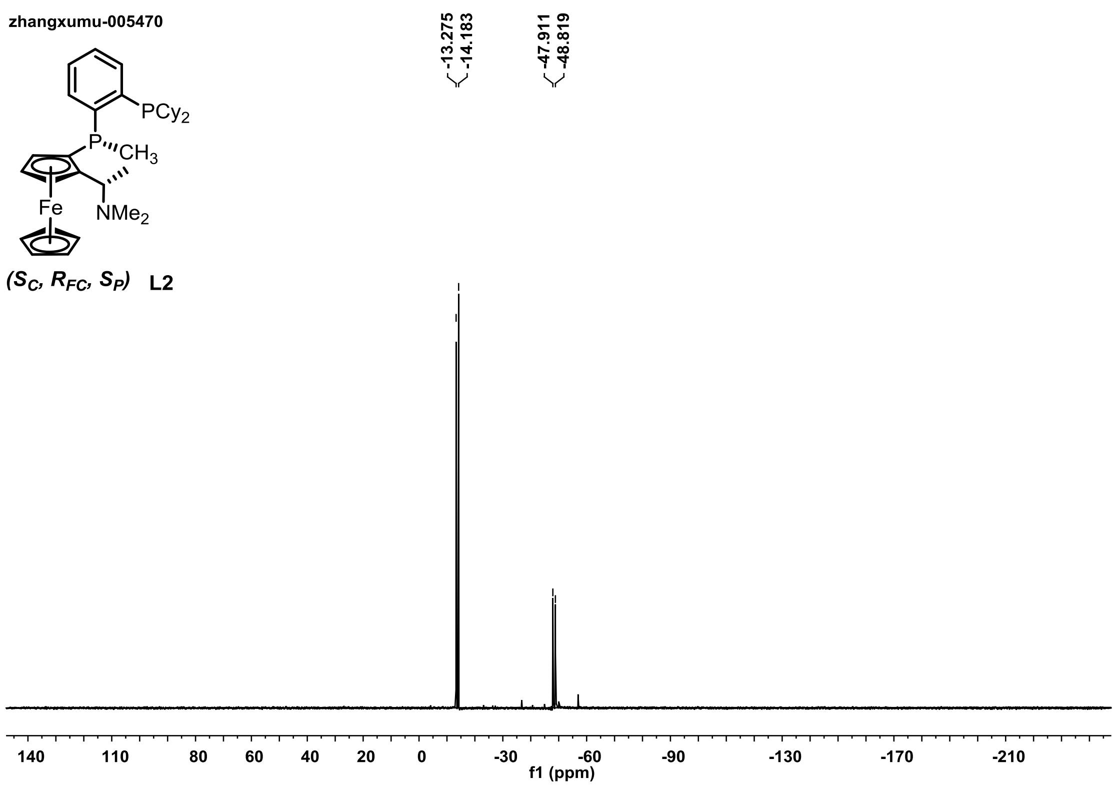
zhangxumu-005470
-13.275
-14.183
-47.911
-48.819
PCy2
P
CH3
Fe
NMe2
(SC, RFC, SP) L2
140
110
80
60
40
20
0
-30
-60
-90
-130
-170
-210
f1 (ppm)

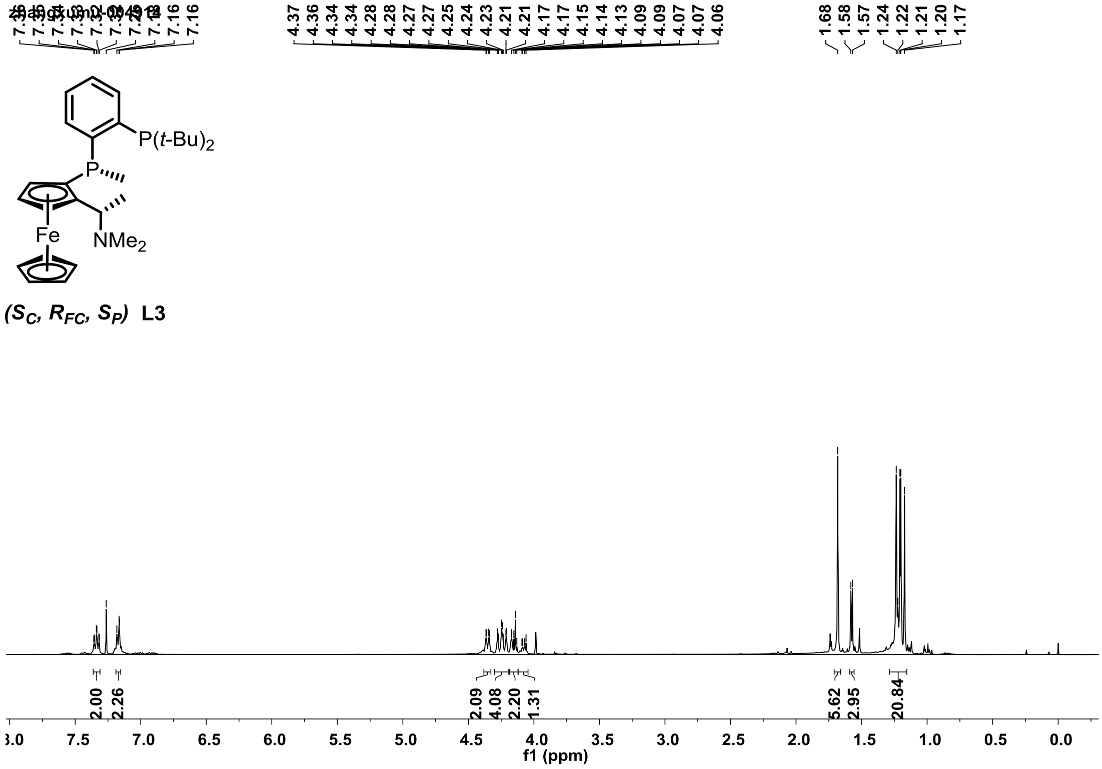
7.16
7.16
4.37
4.36
4.34
4.34
4.28
4.28
4.27
4.27
4.25
4.24
4.23
4.21
4.21
4.17
4.17
4.15
4.14
4.13
4.09
4.09
4.07
4.07
4.06
1.68
1.58
1.57
1.24
1.22
1.21
1.20
1.17
P(t-Bu)2
P
Fe
NMe2
(SC, RFC, SP) L3
2.00
2.26
2.09
4.08
2.20
1.31
5.62
2.95
20.84
7.5
7.0
6.5
6.0
5.5
5.0
4.5
4.0
3.5
3.0
2.5
2.0
1.5
1.0
0.5
0.0
f1 (ppm)

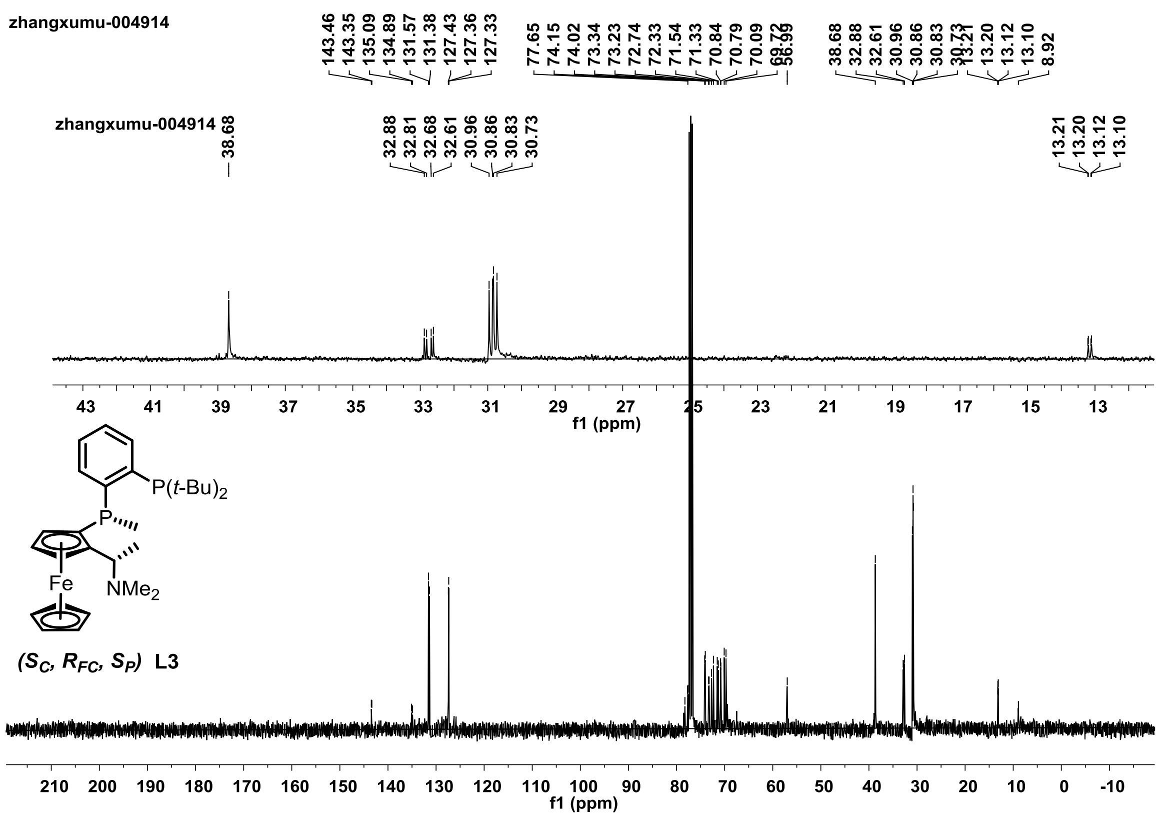

(S_C, R_{FC}, S_P) **L3**

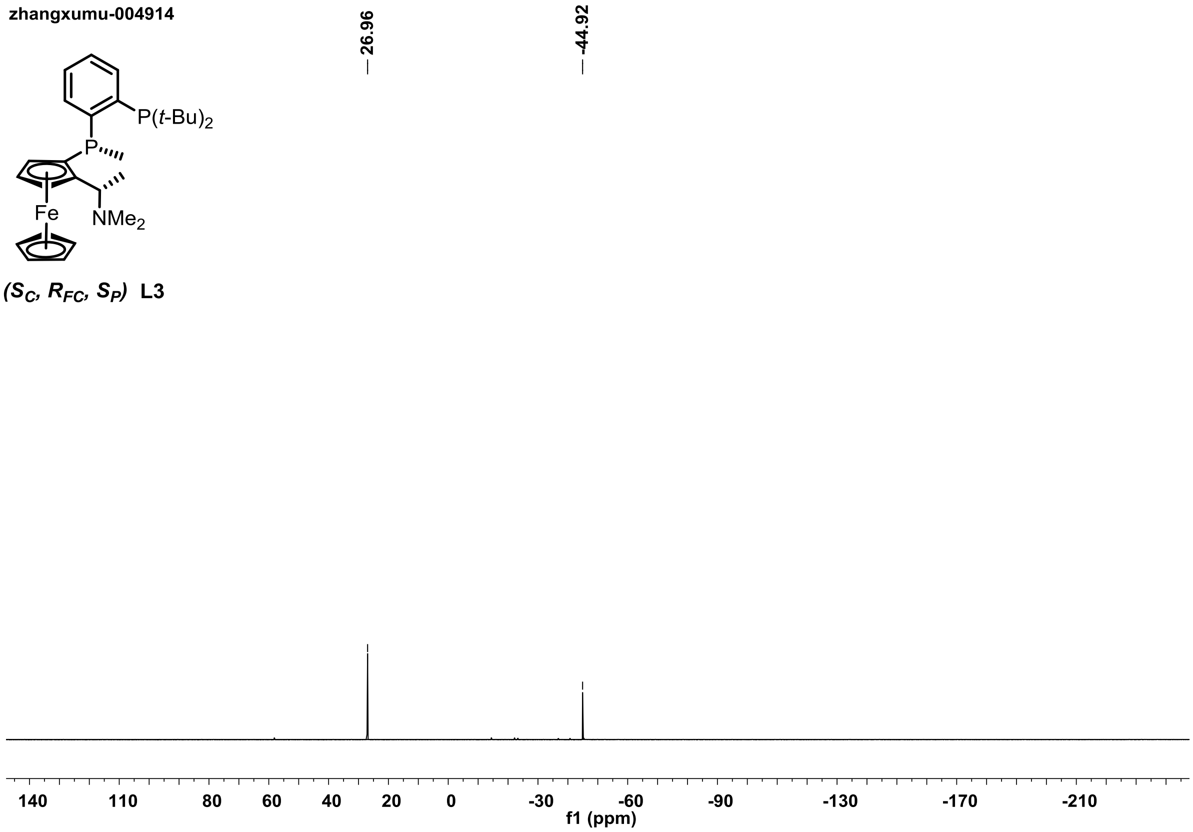

(S_C, R_{FC}, S_P) **L3**

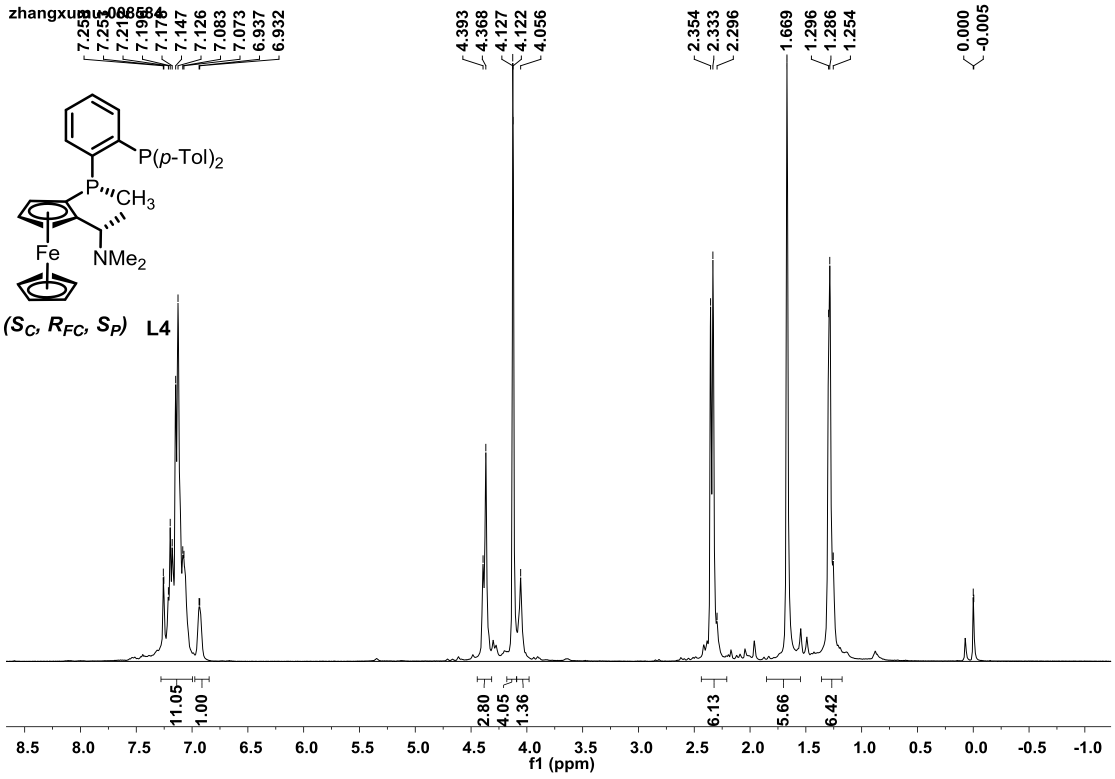
7.147
7.126
7.083
7.073
6.937
6.932
4.393
4.368
4.127
4.122
4.056
2.354
2.333
2.296
1.669
1.296
1.286
1.254
0.000
-0.005
P(p-Tol)2
P
CH3
Fe
NMe2
(SC, RFC, SP) L4
11.05
1.00
2.80
4.05
1.36
6.13
5.66
6.42
8.5 8.0 7.5 7.0 6.5 6.0 5.5 5.0 4.5 4.0 3.5 3.0 2.5 2.0 1.5 1.0 0.5 0.0 -0.5 -1.0
f1 (ppm)

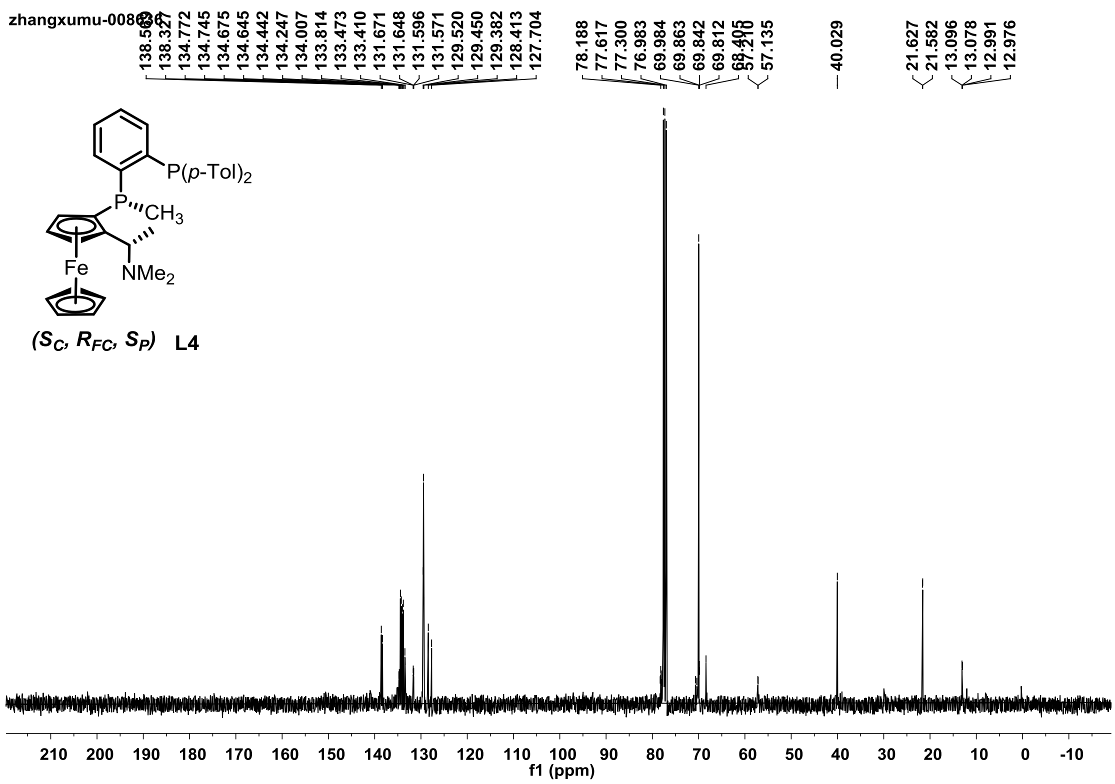
138.327
134.772
134.745
134.675
134.645
134.442
134.247
134.007
133.814
133.473
133.410
131.671
131.648
131.596
131.571
129.520
129.450
129.382
128.413
127.704
78.188
77.617
77.300
76.983
69.984
69.863
69.842
69.812
57.135
40.029
21.627
21.582
13.096
13.078
12.991
12.976
P(p-Tol)2
P
CH3
Fe
NMe2
(SC, RFC, SP) L4
210 200 190 180 170 160 150 140 130 120 110 100 90 80 70 60 50 40 30 20 10 0 -10
f1 (ppm)

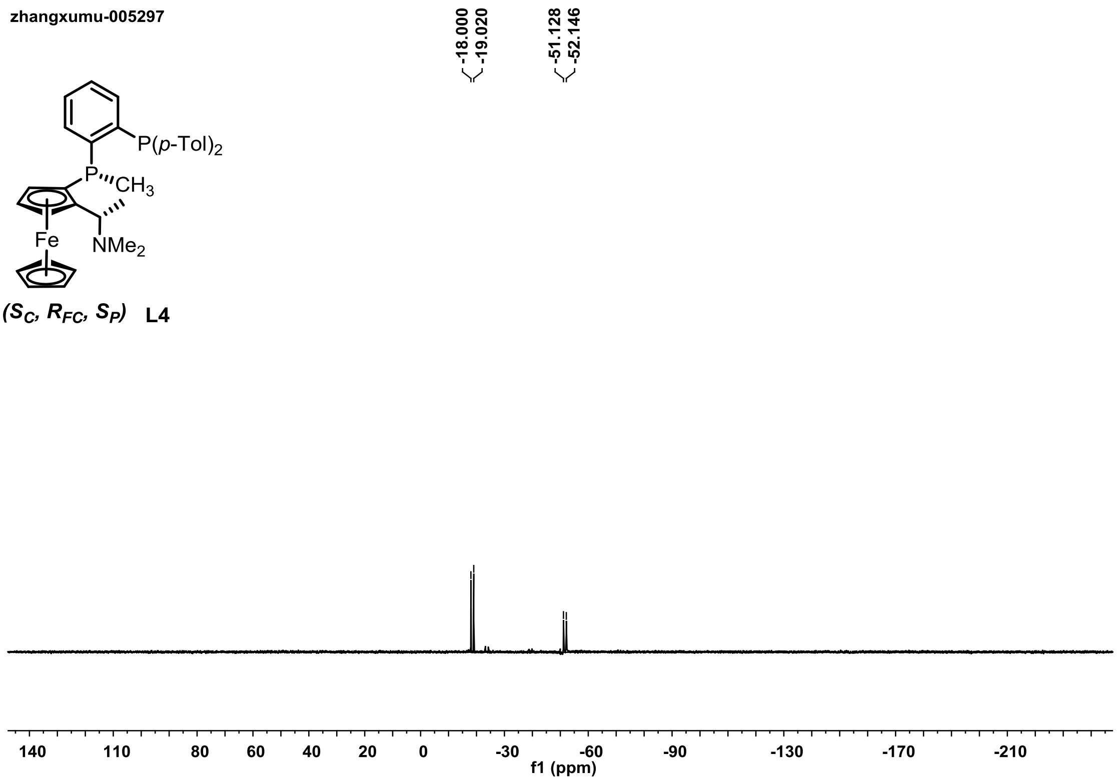

zhangxumu-005297
-18.000
-19.020
-51.128
-52.146
P(p-Tol)2
CH3
Fe
NMe2
(SC, RFC, SP) L4
140 110 80 60 40 20 0 -30 -60 -90 -130 -170 -210
f1 (ppm)

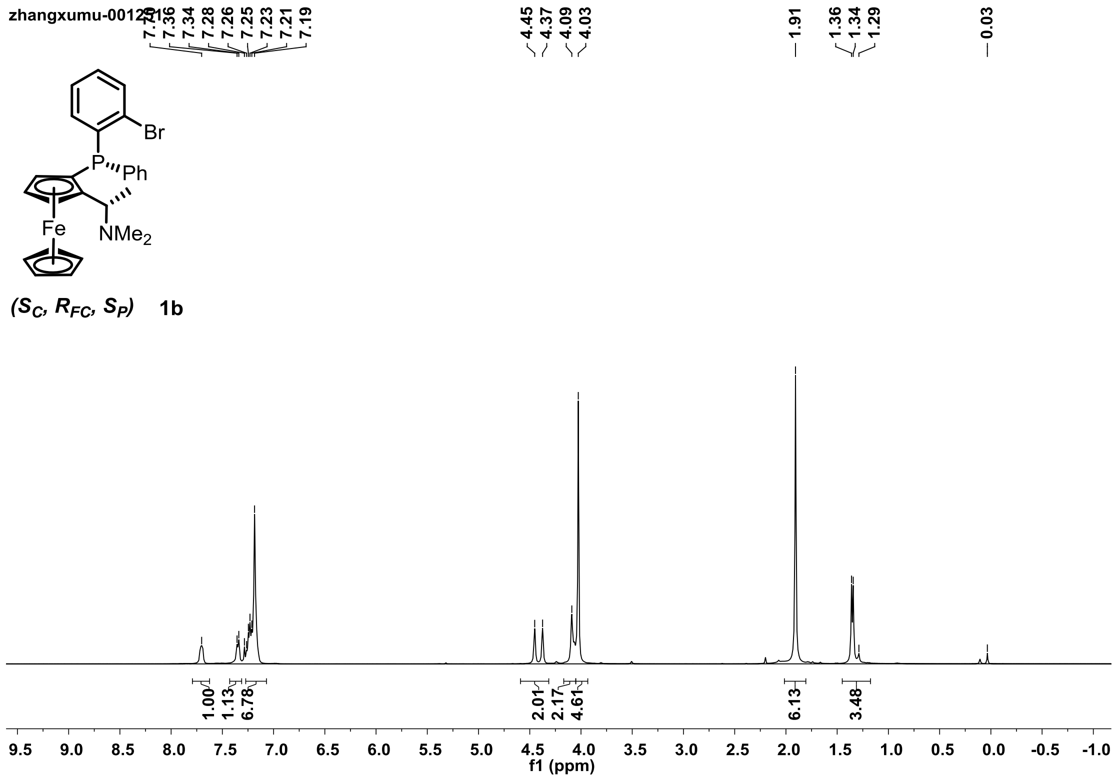

7.36 7.34 7.28 7.26 7.25 7.23 7.21 7.19
4.45 4.37 4.09 4.03
1.91
1.36 1.34 1.29
0.03
Br
Ph
Fe
NMe2
(SC, RFC, SP) 1b
1.00 1.13 6.78
2.01 2.17 4.61
6.13
3.48
9.5 9.0 8.5 8.0 7.5 7.0 6.5 6.0 5.5 5.0 4.5 4.0 3.5 3.0 2.5 2.0 1.5 1.0 0.5 0.0 -0.5 -1.0
f1 (ppm)

zhangxumu-001251

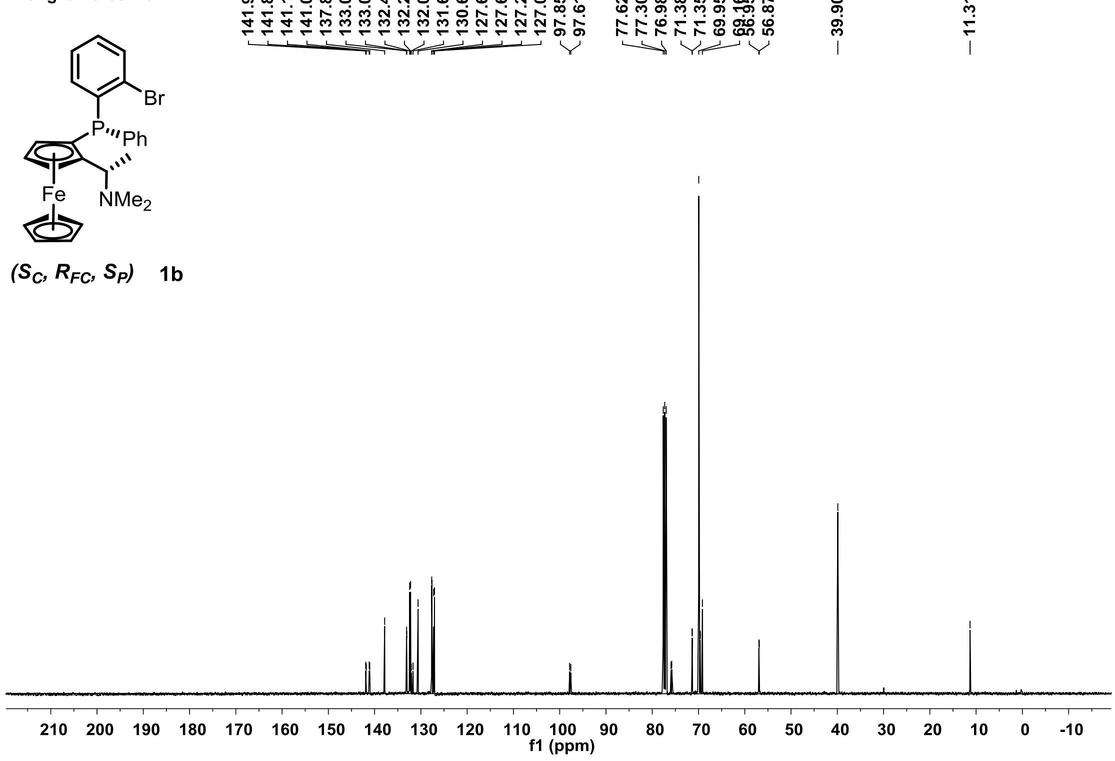

(S_C, R_{FC}, S_P) **1b**

zhangxumu-001251

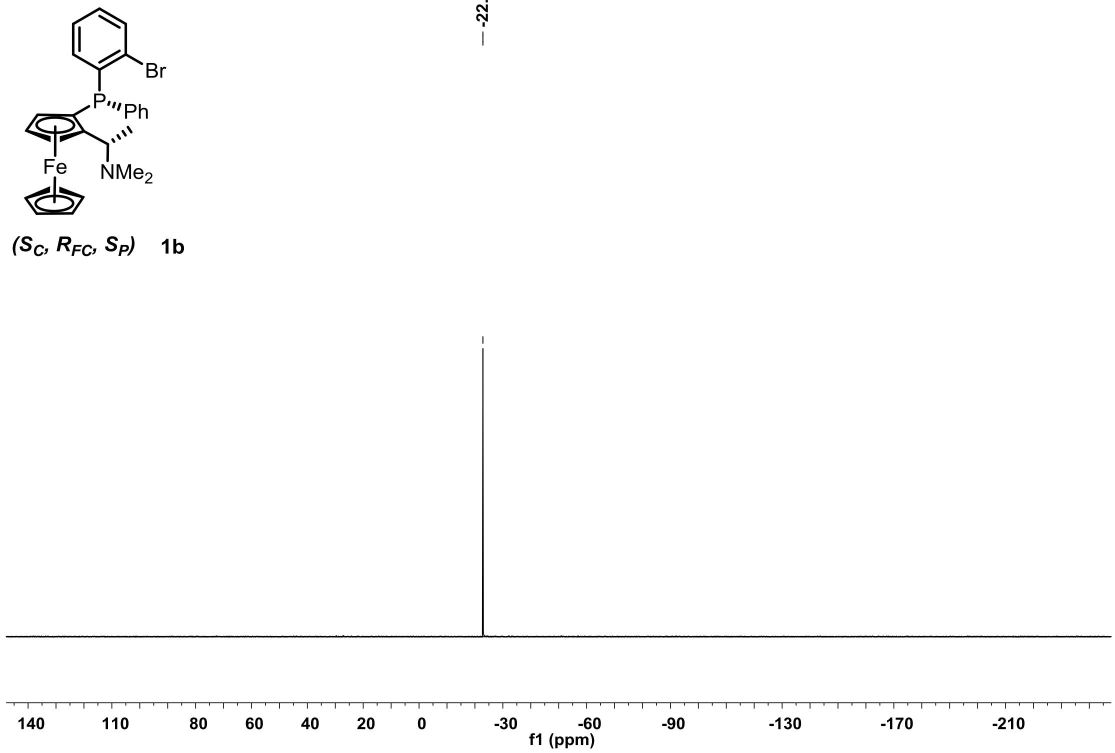

(S_C, R_{FC}, S_P) **1b**

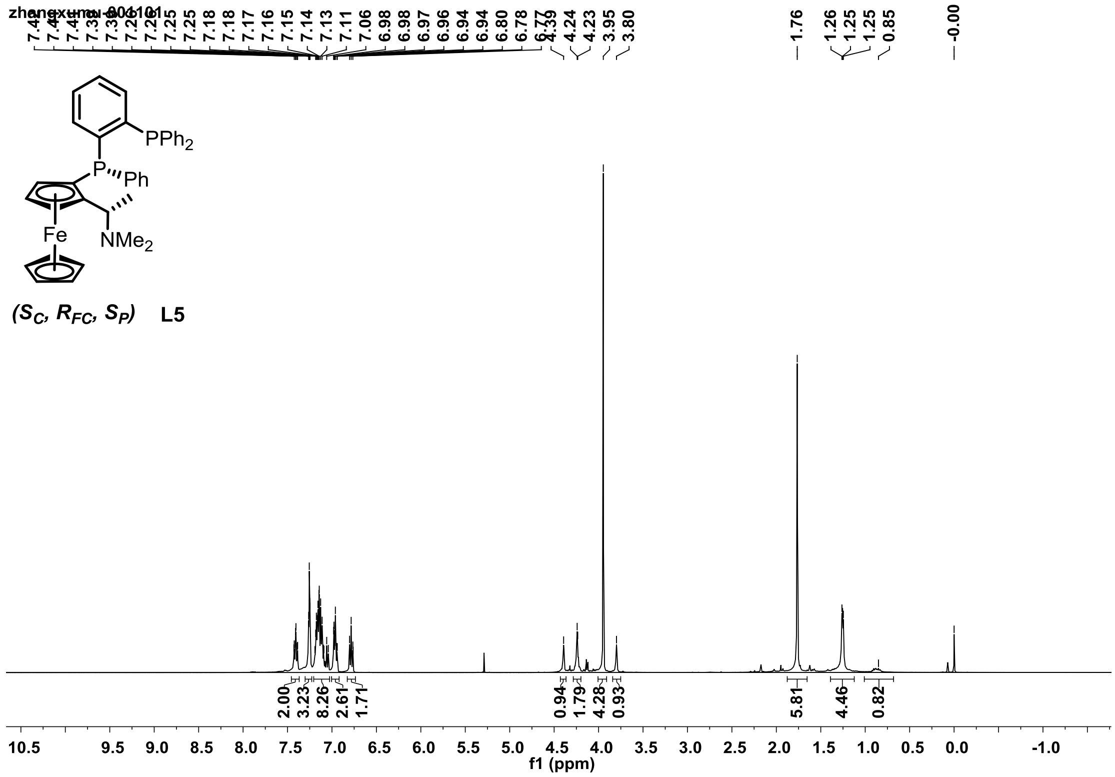
PPh2
P
Ph
Fe
NMe2
(SC, RFC, SP) L5
7.14 7.13 7.11 7.06 6.98 6.98 6.97 6.96 6.94 6.94 6.80 6.78
4.24 4.23 3.95 3.80
1.76
1.26 1.25 1.25 0.85
-0.00
2.00 3.23 8.26 2.61 1.71
0.94 1.79 4.28 0.93
5.81 4.46 0.82
f1 (ppm)

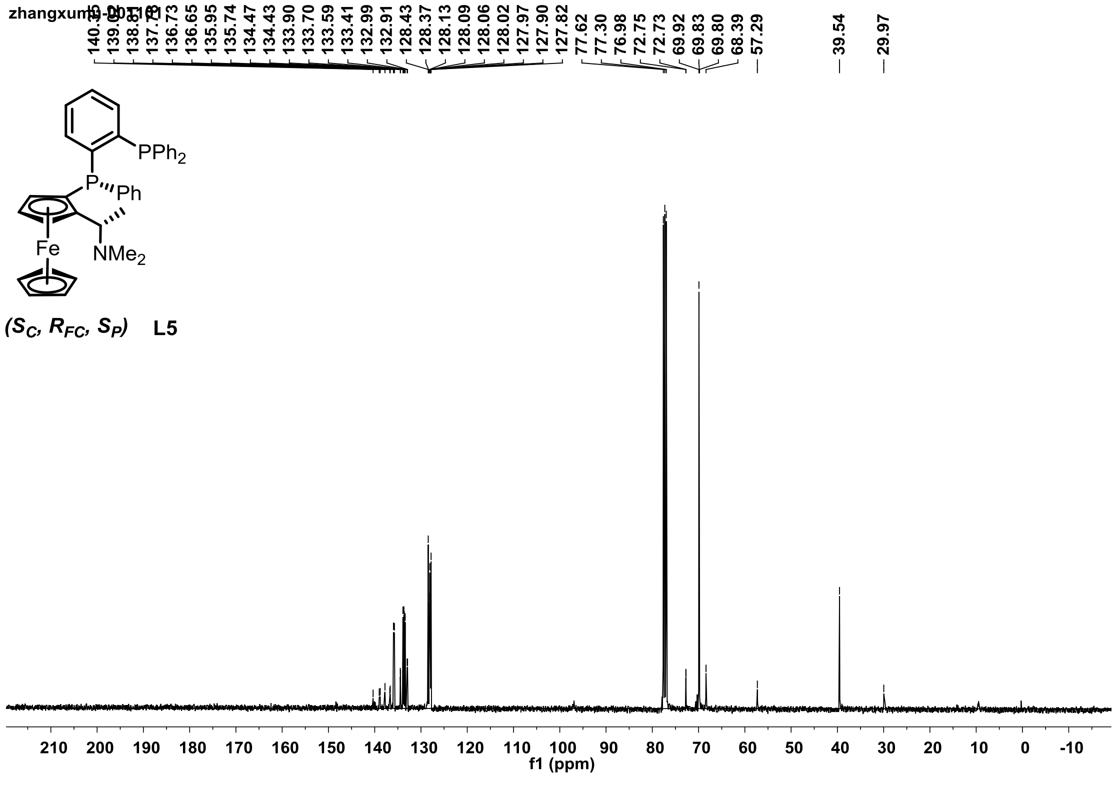
PPh2
P
Ph
Fe
NMe2
(SC, RFC, SP) L5
137.73 136.65 135.95 135.74 134.47 134.43 133.90 133.70 133.59 133.41 132.99 132.91 128.43 128.37 128.13 128.09 128.06 128.02 127.97 127.90 127.82
77.62 77.30 76.98 72.75 72.73 69.92 69.83 69.80 68.39 57.29
39.54
29.97
f1 (ppm)

zhangxumu-001101

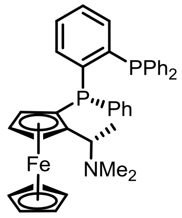

(S$_C$, R$_{FC}$, S$_P$) **L5**

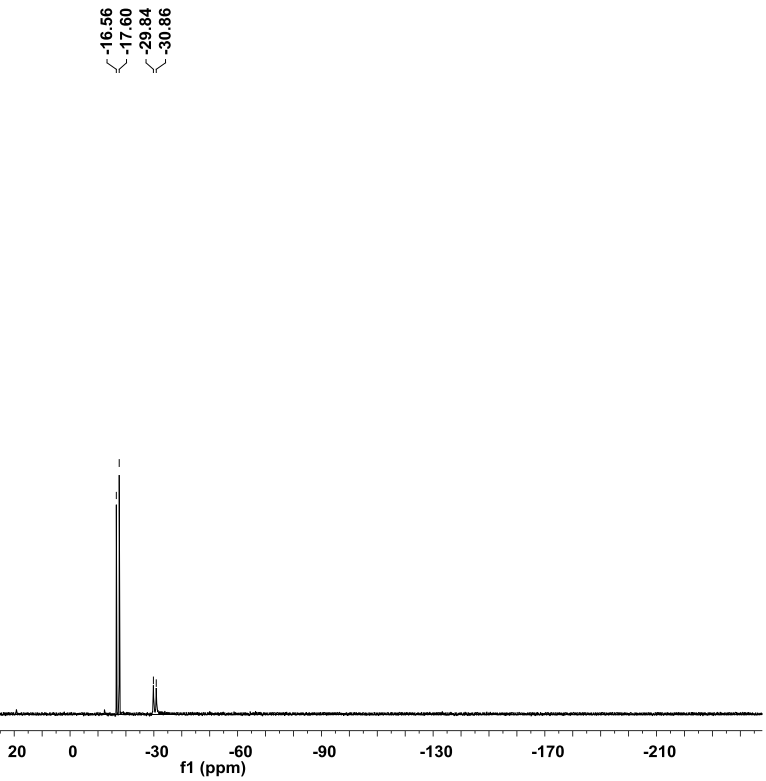

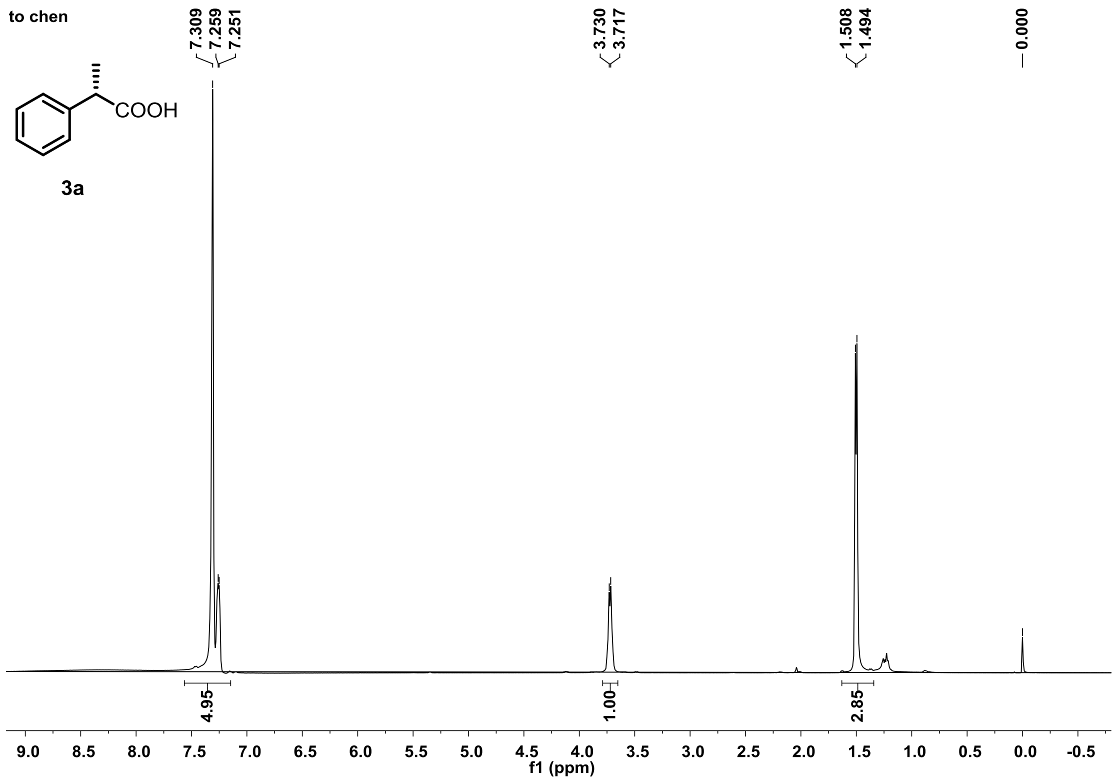

to chen
7.309
7.259
7.251
3.730
3.717
1.508
1.494
0.000
COOH
3a
4.95
1.00
2.85
9.0 8.5 8.0 7.5 7.0 6.5 6.0 5.5 5.0 4.5 4.0 3.5 3.0 2.5 2.0 1.5 1.0 0.5 0.0 -0.5
f1 (ppm)

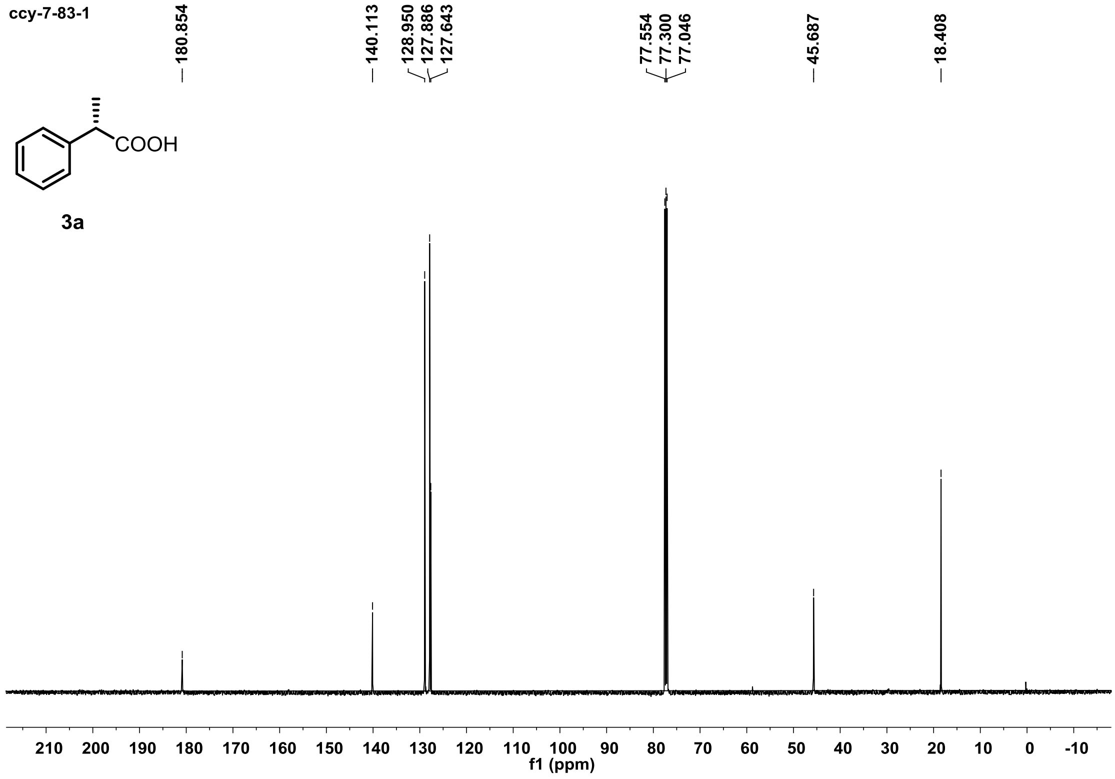

ccy-7-83-1
180.854
140.113
128.950
127.886
127.643
77.554
77.300
77.046
45.687
18.408
COOH
3a
210 200 190 180 170 160 150 140 130 120 110 100 90 80 70 60 50 40 30 20 10 0 -10
f1 (ppm)

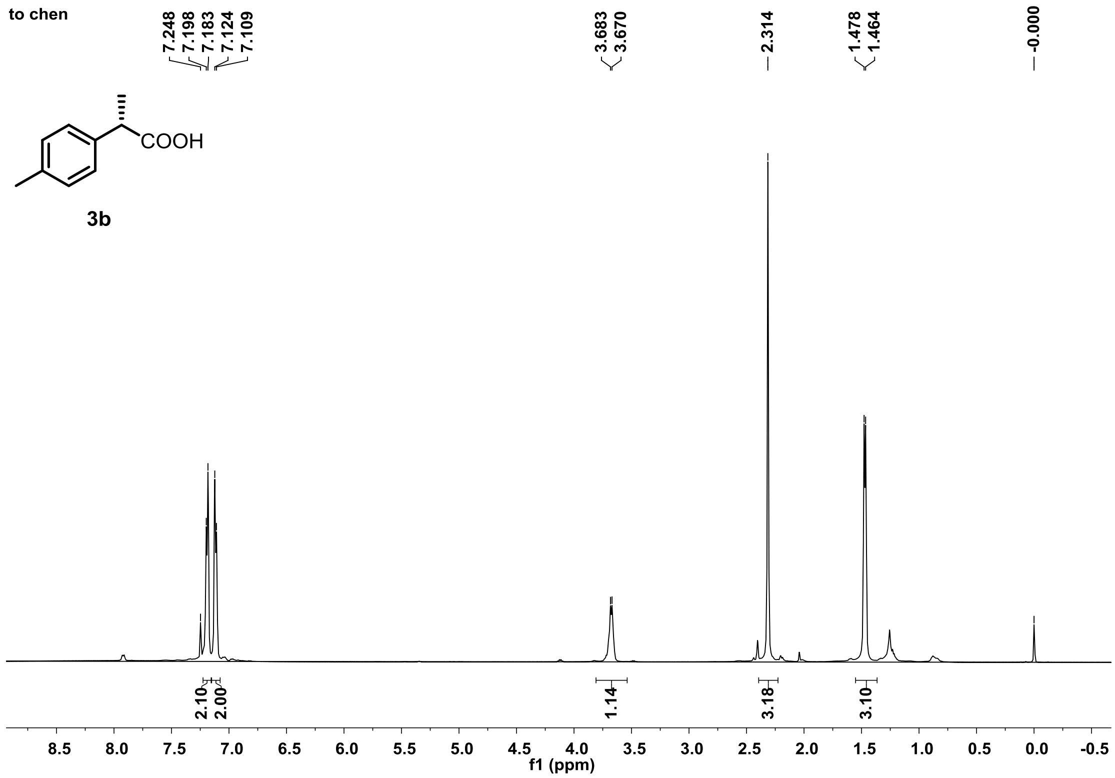

to chen
7.248
7.198
7.183
7.124
7.109
3.683
3.670
2.314
1.478
1.464
-0.000
COOH
3b
2.10
2.00
1.14
3.18
3.10
8.5 8.0 7.5 7.0 6.5 6.0 5.5 5.0 4.5 4.0 3.5 3.0 2.5 2.0 1.5 1.0 0.5 0.0 -0.5
f1 (ppm)

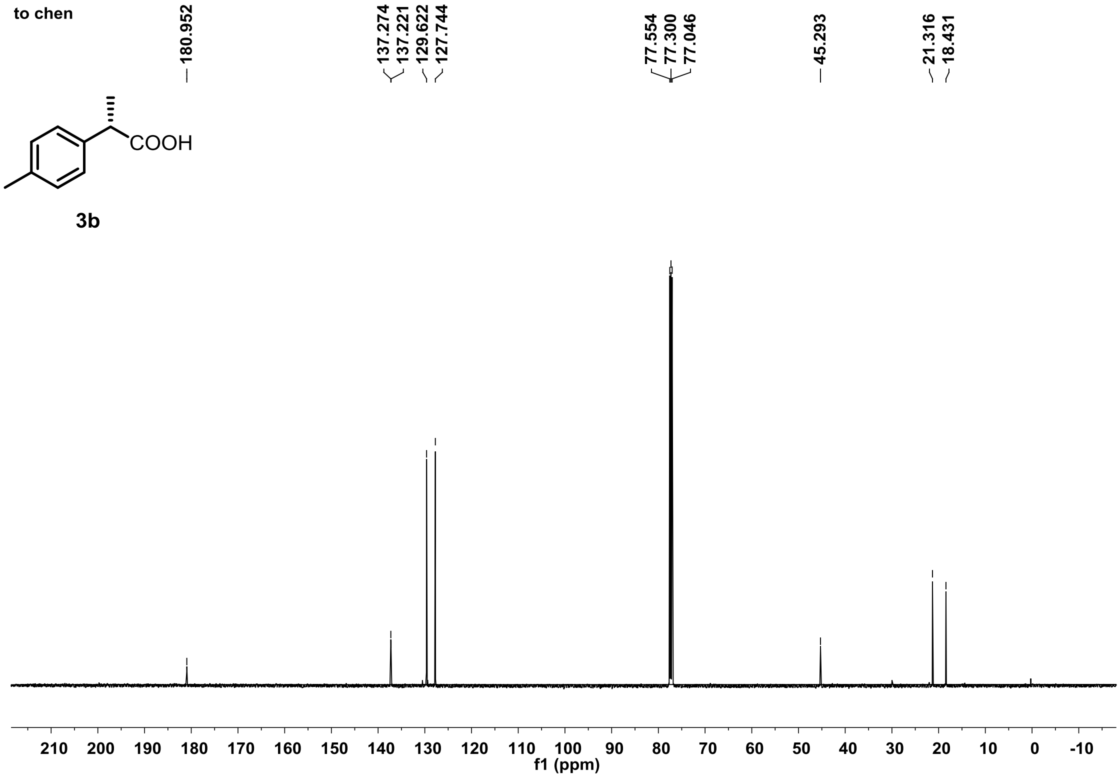

to chen
180.952
137.274
137.221
129.622
127.744
77.554
77.300
77.046
45.293
21.316
18.431
COOH
3b
210 200 190 180 170 160 150 140 130 120 110 100 90 80 70 60 50 40 30 20 10 0 -10
f1 (ppm)

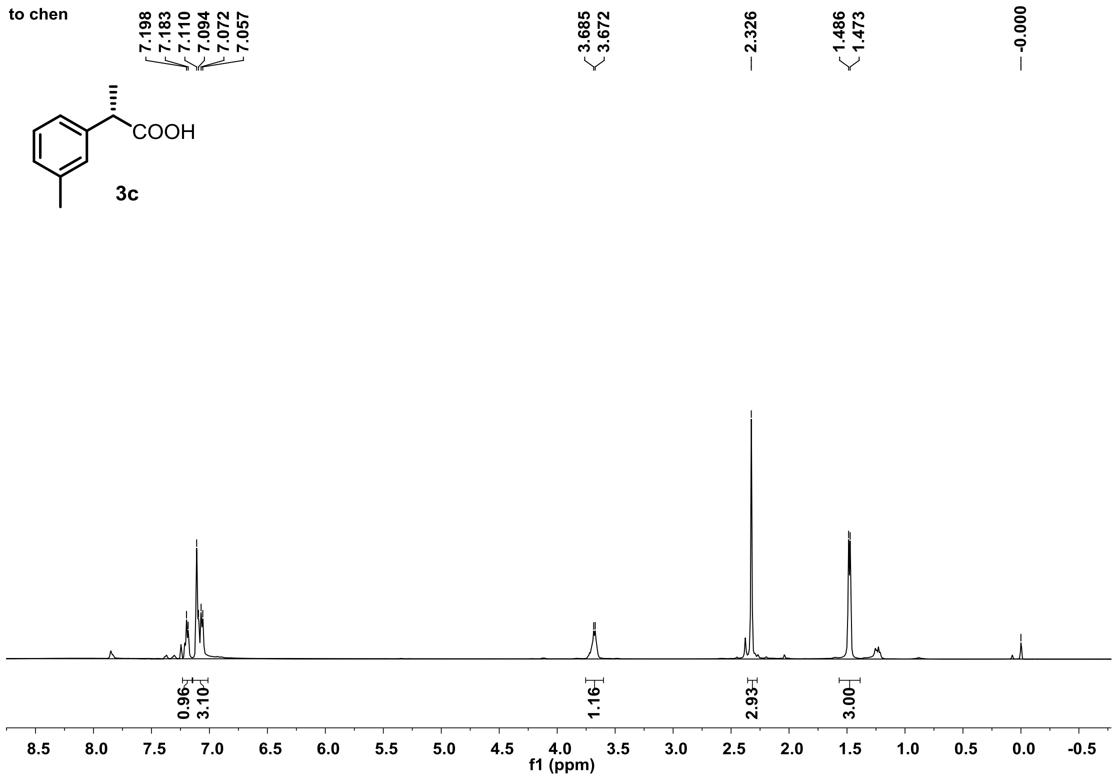
to chen
7.198
7.183
7.110
7.094
7.072
7.057
3.685
3.672
2.326
1.486
1.473
-0.000
COOH
3c
0.96
3.10
1.16
2.93
3.00
8.5 8.0 7.5 7.0 6.5 6.0 5.5 5.0 4.5 4.0 3.5 3.0 2.5 2.0 1.5 1.0 0.5 0.0 -0.5
f1 (ppm)

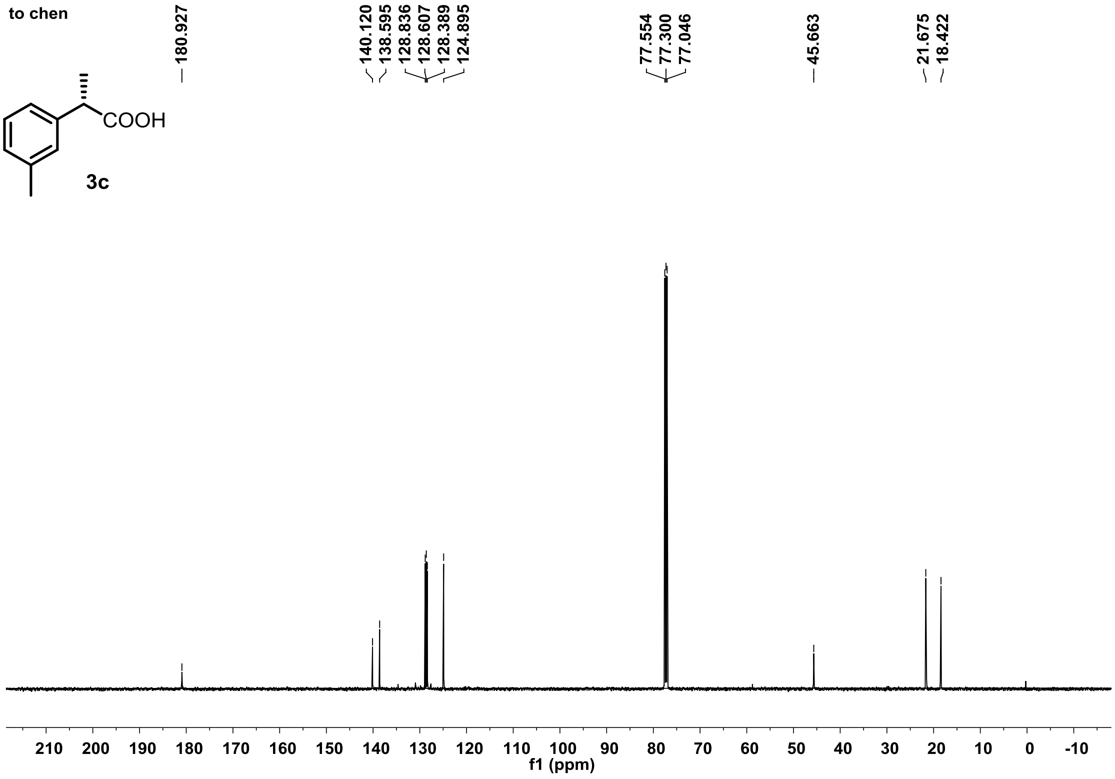
to chen
180.927
140.120
138.595
128.836
128.607
128.389
124.895
77.554
77.300
77.046
45.663
21.675
18.422
COOH
3c
210 200 190 180 170 160 150 140 130 120 110 100 90 80 70 60 50 40 30 20 10 0 -10
f1 (ppm)

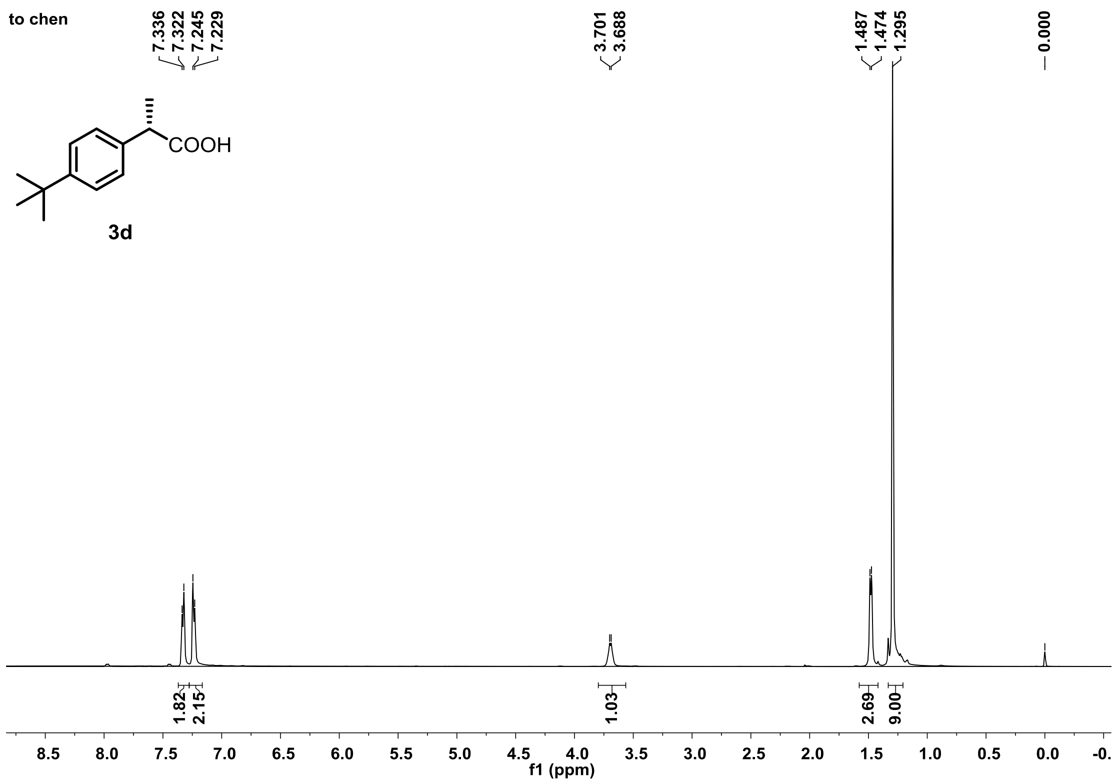
to chen
7.336
7.322
7.245
7.229
3.701
3.688
1.487
1.474
1.295
0.000
COOH
3d
1.82
2.15
1.03
2.69
9.00
8.5 8.0 7.5 7.0 6.5 6.0 5.5 5.0 4.5 4.0 3.5 3.0 2.5 2.0 1.5 1.0 0.5 0.0 -0.
f1 (ppm)

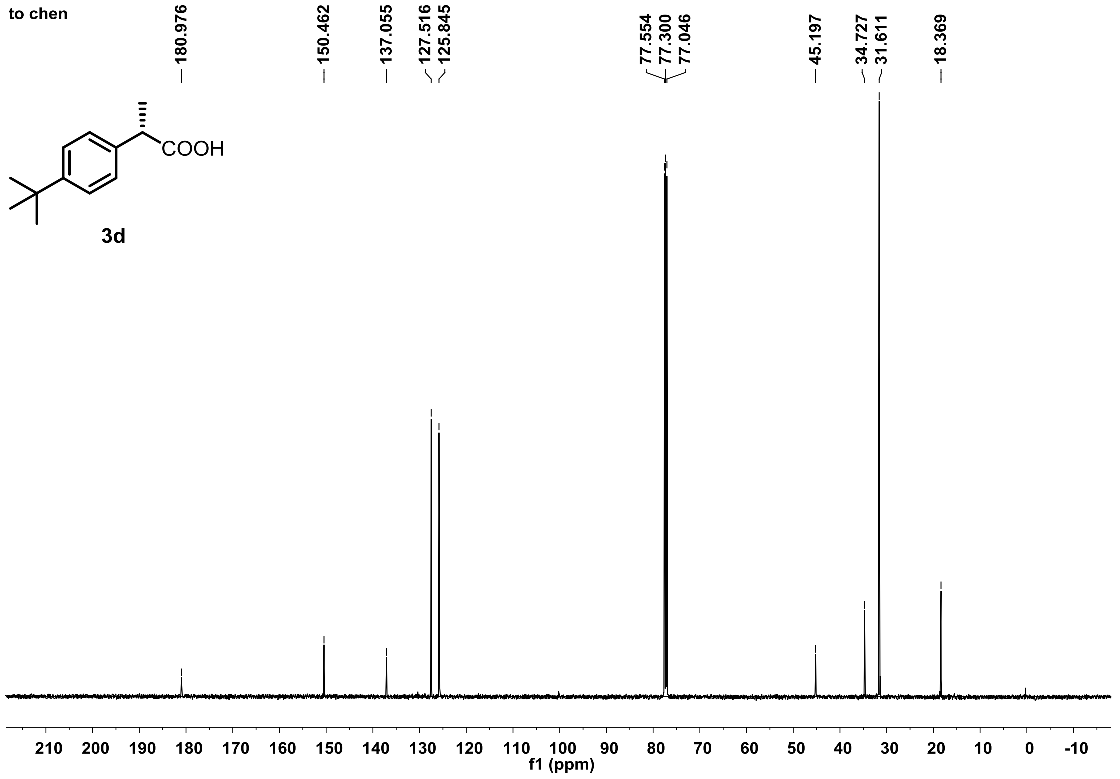
to chen
180.976
150.462
137.055
127.516
125.845
77.554
77.300
77.046
45.197
34.727
31.611
18.369
COOH
3d
210 200 190 180 170 160 150 140 130 120 110 100 90 80 70 60 50 40 30 20 10 0 -10
f1 (ppm)

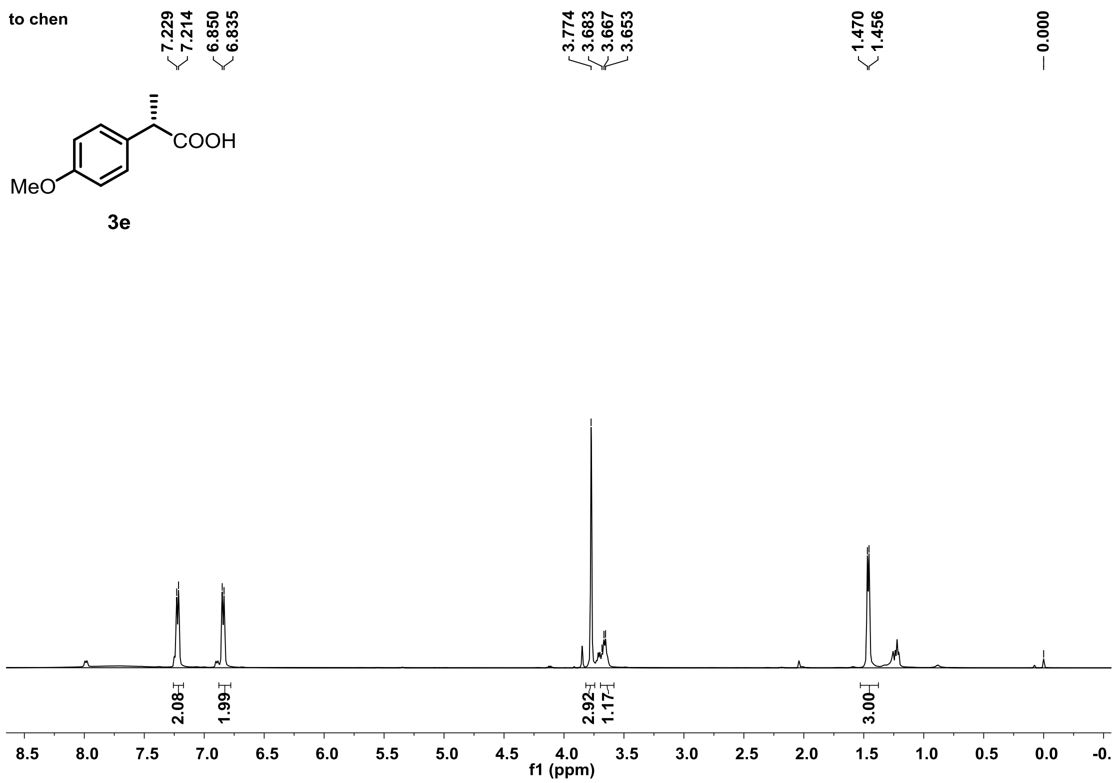
to chen
7.229
7.214
6.850
6.835
3.774
3.683
3.667
3.653
1.470
1.456
0.000
COOH
MeO
3e
2.08
1.99
2.92
1.17
3.00
8.5 8.0 7.5 7.0 6.5 6.0 5.5 5.0 4.5 4.0 3.5 3.0 2.5 2.0 1.5 1.0 0.5 0.0 -0.
f1 (ppm)

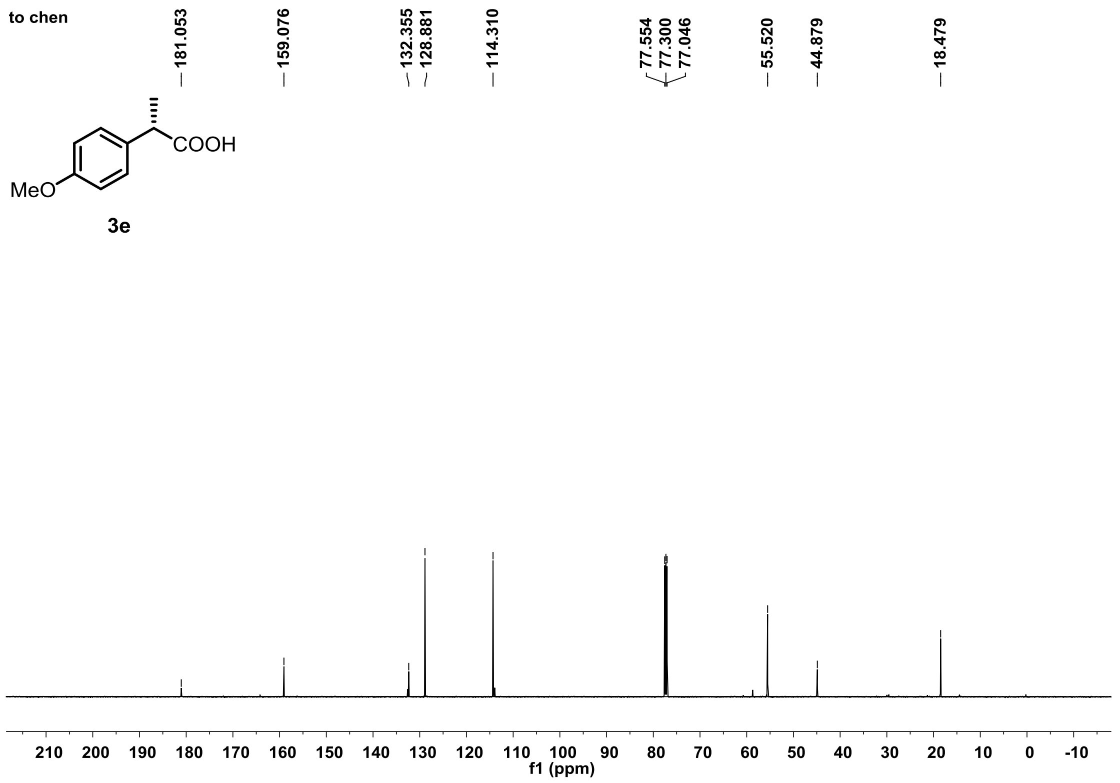
to chen
181.053
159.076
132.355
128.881
114.310
77.554
77.300
77.046
55.520
44.879
18.479
COOH
MeO
3e
210 200 190 180 170 160 150 140 130 120 110 100 90 80 70 60 50 40 30 20 10 0 -10
f1 (ppm)

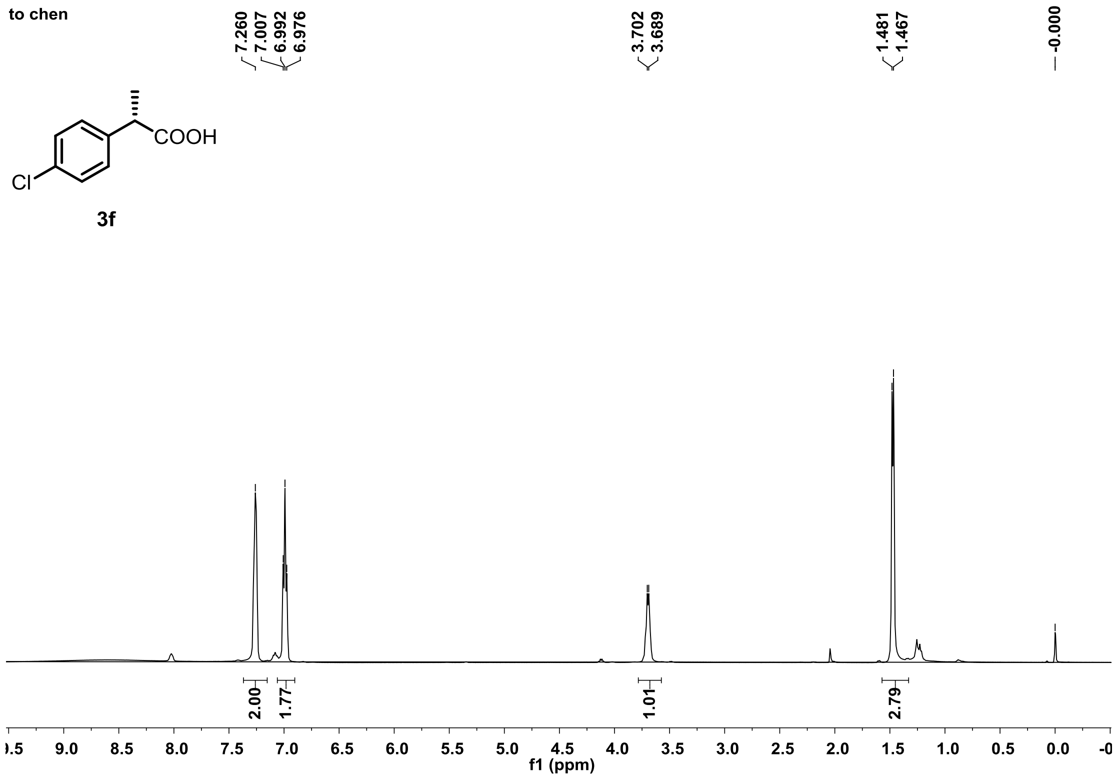
to chen
7.260
7.007
6.992
6.976
3.702
3.689
1.481
1.467
-0.000
COOH
Cl
3f
2.00
1.77
1.01
2.79
f1 (ppm)

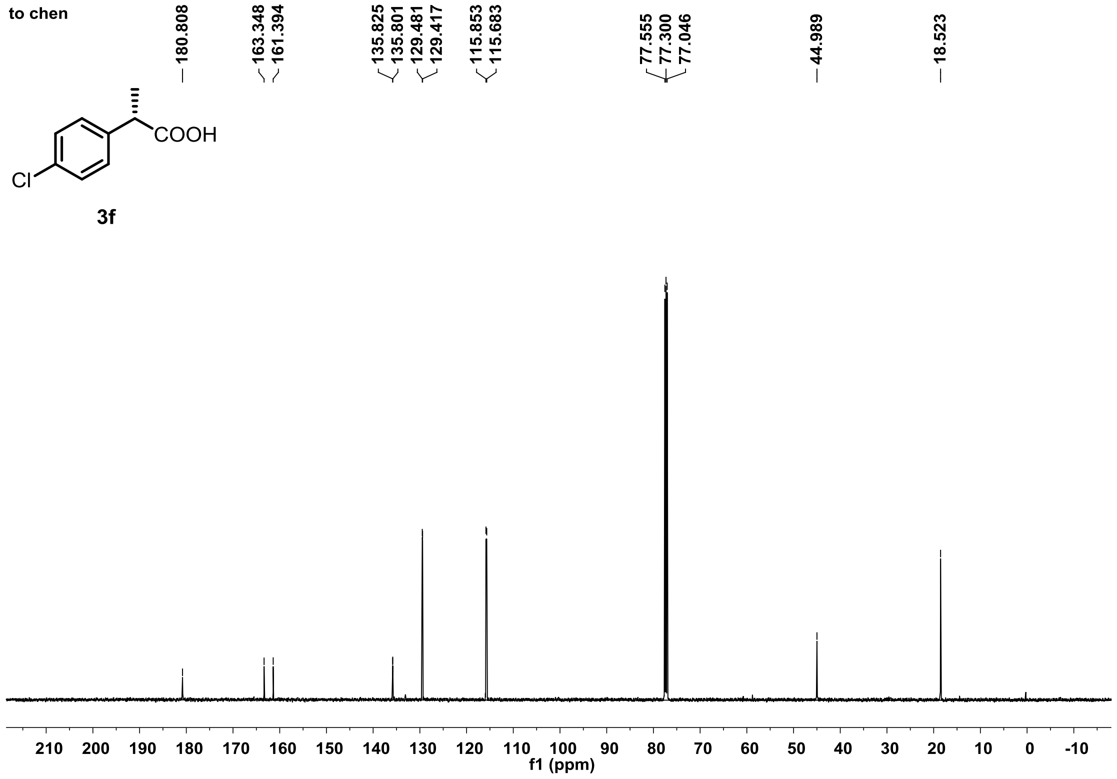
to chen
180.808
163.348
161.394
135.825
135.801
129.481
129.417
115.853
115.683
77.555
77.300
77.046
44.989
18.523
COOH
Cl
3f
f1 (ppm)

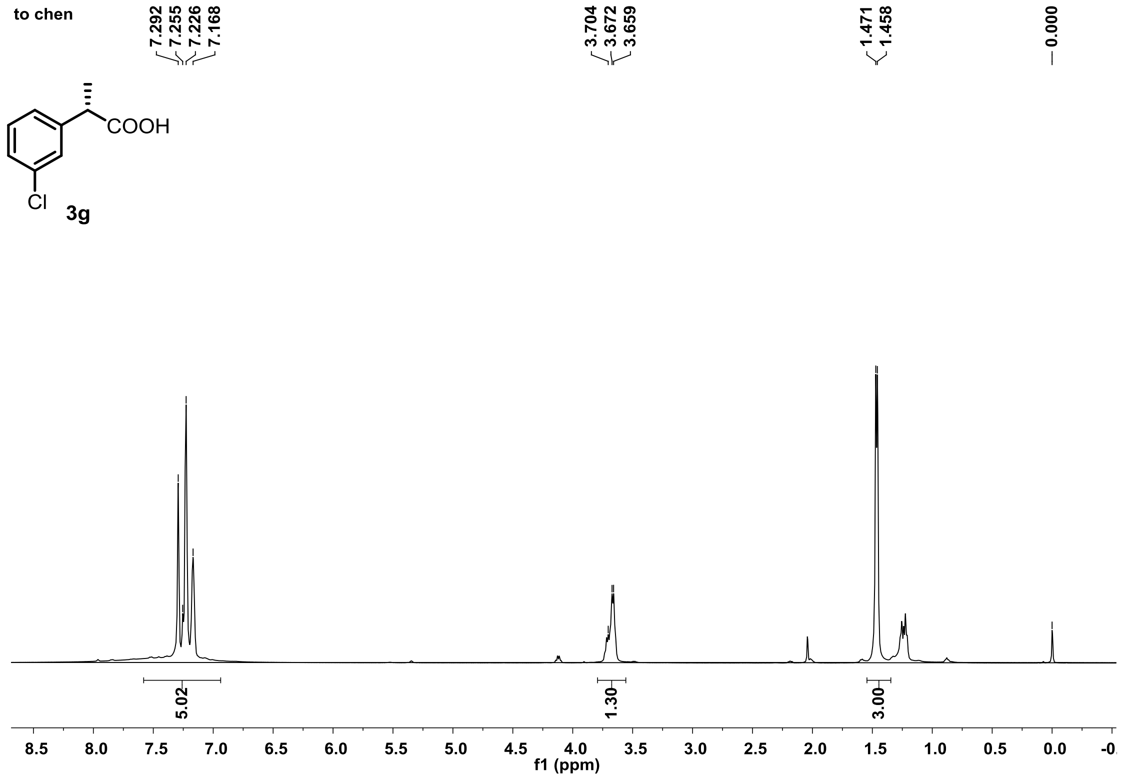

to chen
7.292
7.255
7.226
7.168
3.704
3.672
3.659
1.471
1.458
0.000
COOH
Cl
3g
5.02
1.30
3.00
8.5 8.0 7.5 7.0 6.5 6.0 5.5 5.0 4.5 4.0 3.5 3.0 2.5 2.0 1.5 1.0 0.5 0.0
f1 (ppm)

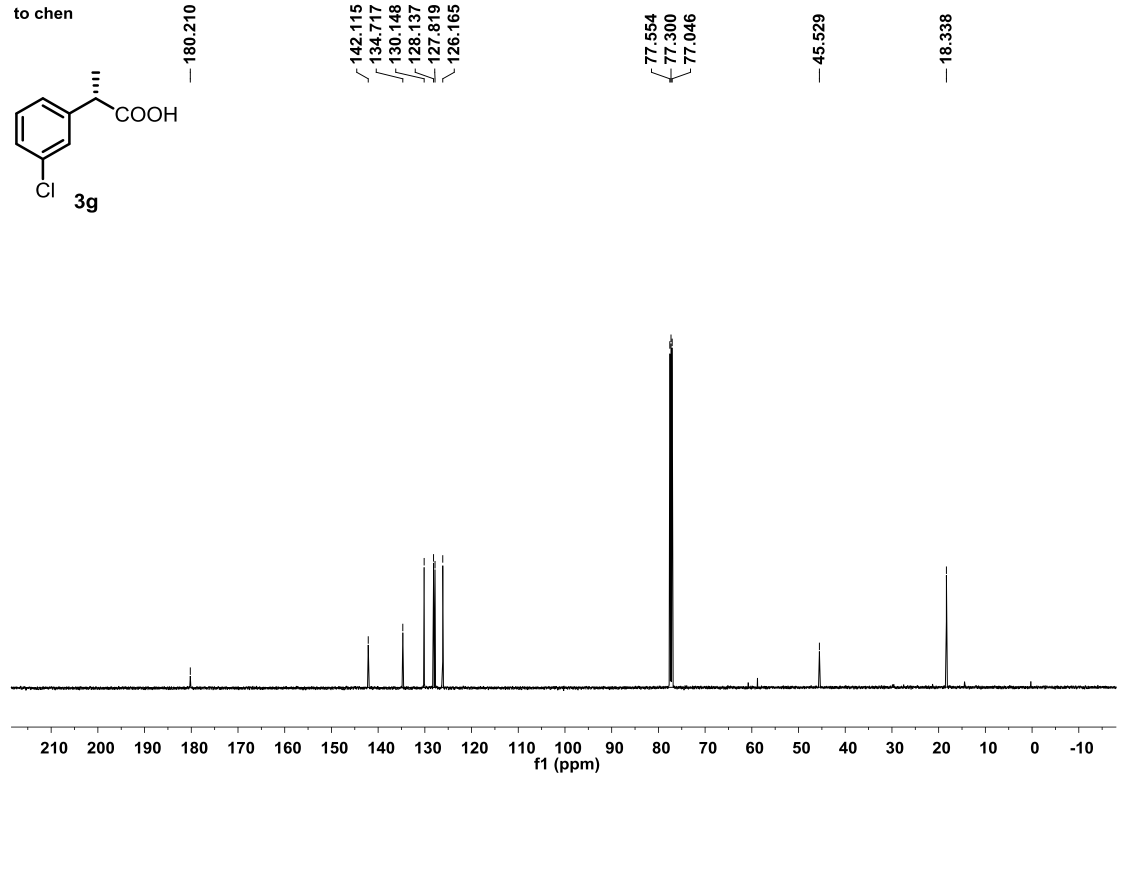

to chen
180.210
142.115
134.717
130.148
128.137
127.819
126.165
77.554
77.300
77.046
45.529
18.338
COOH
Cl
3g
210 200 190 180 170 160 150 140 130 120 110 100 90 80 70 60 50 40 30 20 10 0 -10
f1 (ppm)

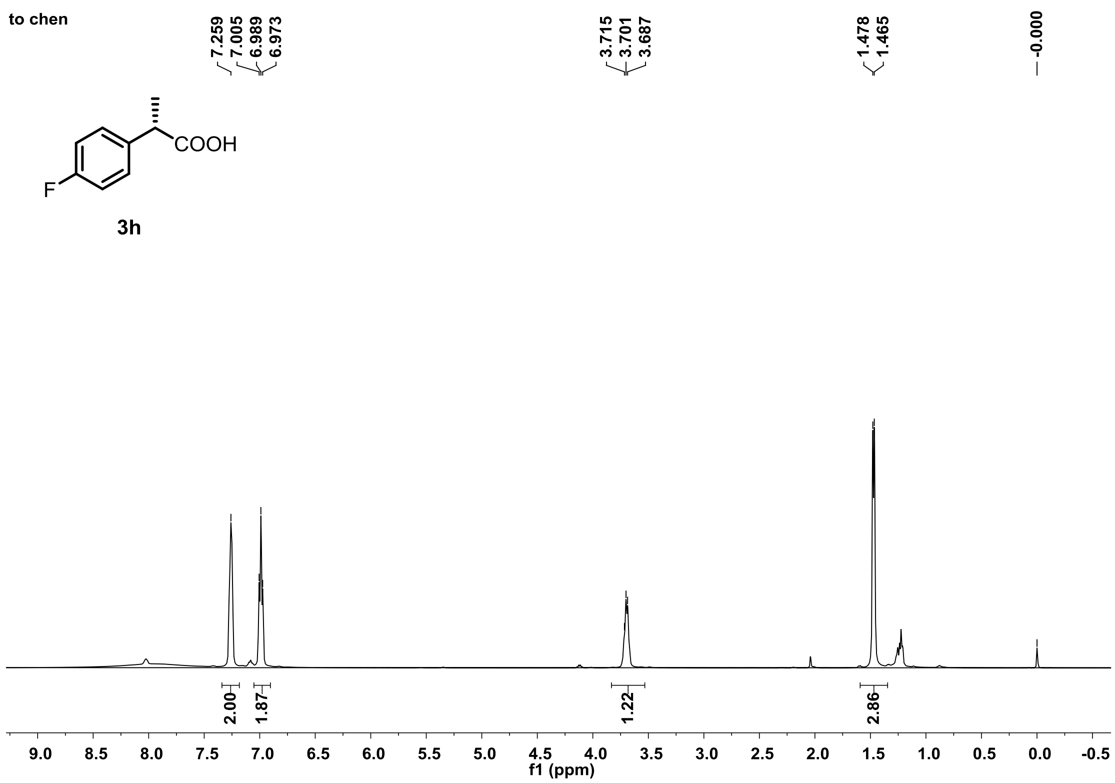
to chen
7.259
7.005
6.989
6.973
3.715
3.701
3.687
1.478
1.465
-0.000
COOH
F
3h
2.00
1.87
1.22
2.86
9.0 8.5 8.0 7.5 7.0 6.5 6.0 5.5 5.0 4.5 4.0 3.5 3.0 2.5 2.0 1.5 1.0 0.5 0.0 -0.5
f1 (ppm)

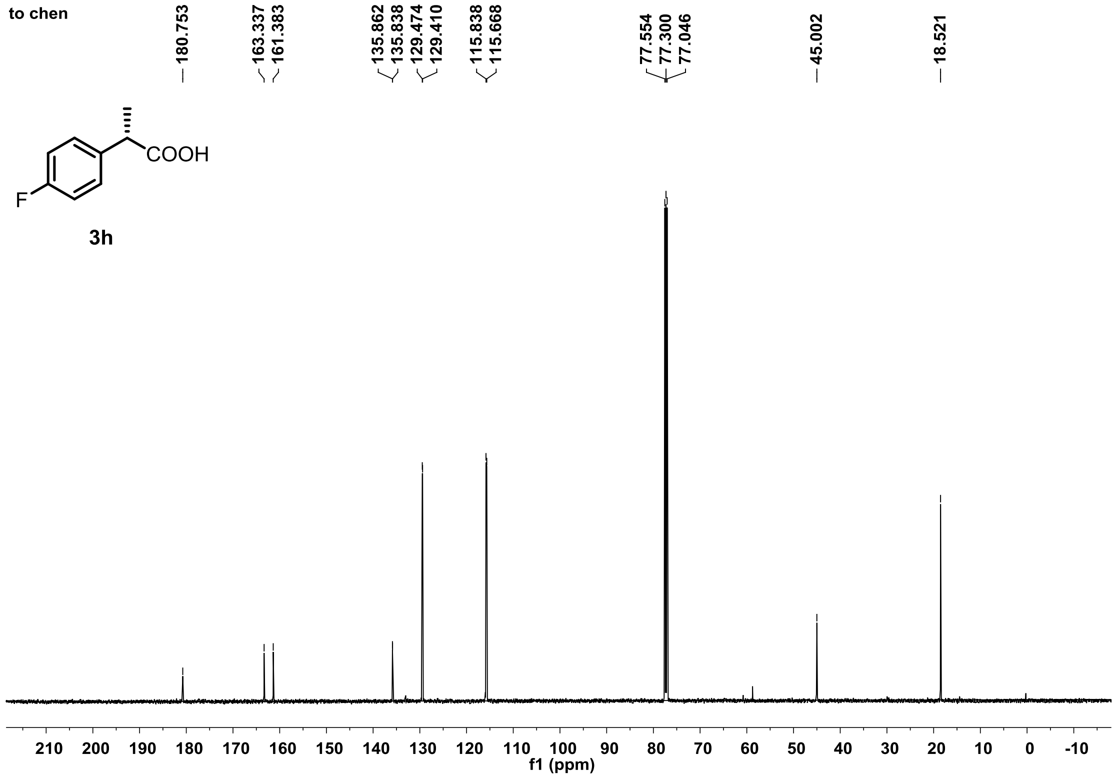
to chen
180.753
163.337
161.383
135.862
135.838
129.474
129.410
115.838
115.668
77.554
77.300
77.046
45.002
18.521
COOH
F
3h
210 200 190 180 170 160 150 140 130 120 110 100 90 80 70 60 50 40 30 20 10 0 -10
f1 (ppm)

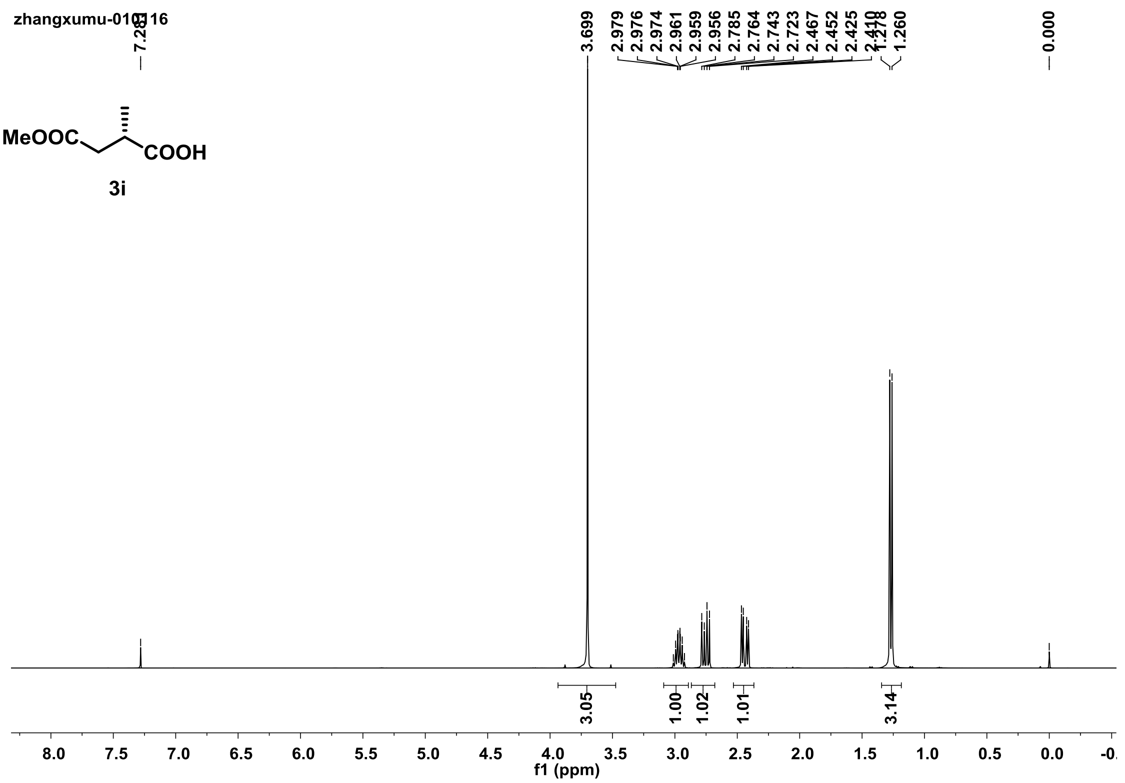
zhangxumu-010116
3.699
2.979
2.976
2.974
2.961
2.959
2.956
2.785
2.764
2.743
2.723
2.467
2.452
2.425
1.260
0.000
MeOOC
COOH
3i
3.05
1.00
1.02
1.01
3.14
8.0 7.5 7.0 6.5 6.0 5.5 5.0 4.5 4.0 3.5 3.0 2.5 2.0 1.5 1.0 0.5 0.0
f1 (ppm)

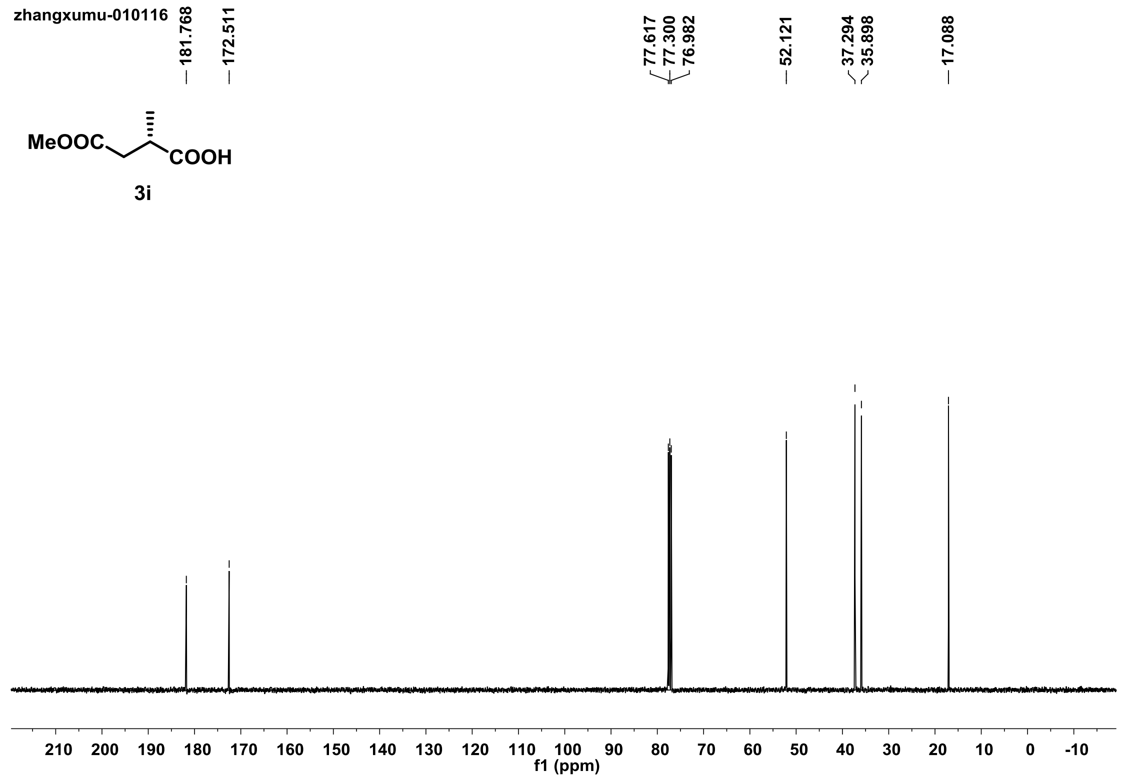
zhangxumu-010116
181.768
172.511
77.617
77.300
76.982
52.121
37.294
35.898
17.088
MeOOC
COOH
3i
210 200 190 180 170 160 150 140 130 120 110 100 90 80 70 60 50 40 30 20 10 0 -10
f1 (ppm)

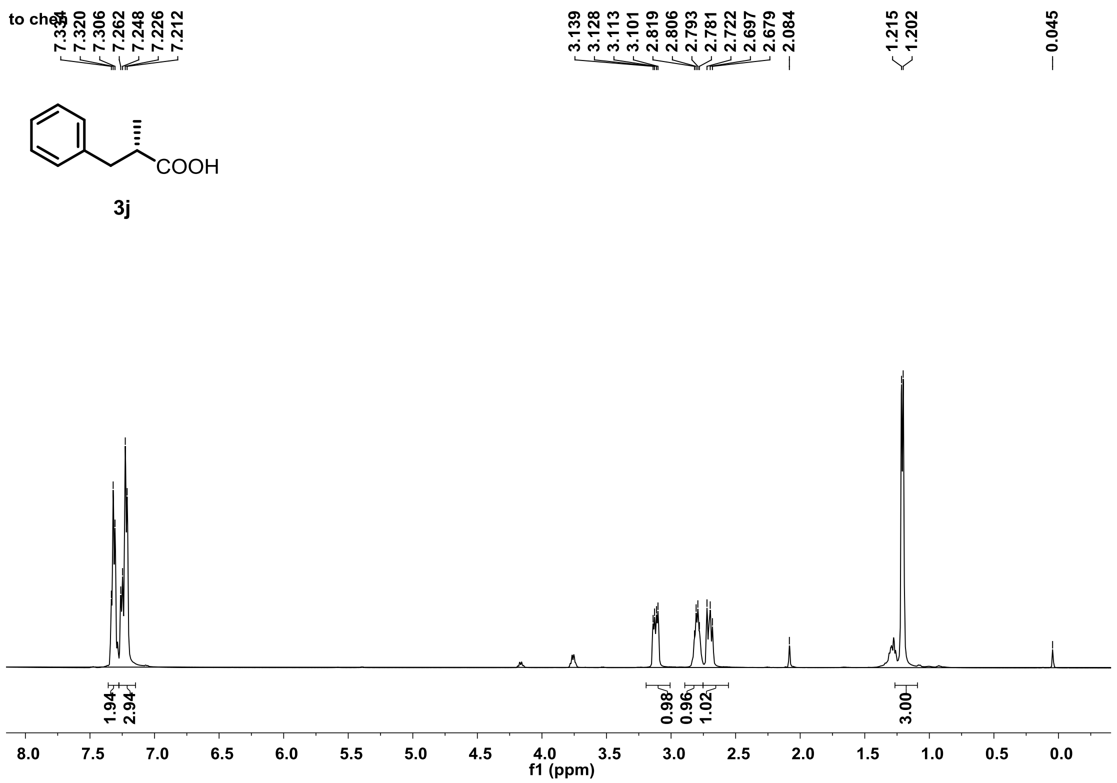
to chen
7.334
7.320
7.306
7.262
7.248
7.226
7.212
3.139
3.128
3.113
3.101
2.819
2.806
2.793
2.781
2.722
2.697
2.679
2.084
1.215
1.202
0.045
COOH
3j
1.94
2.94
0.98
0.96
1.02
3.00
8.0 7.5 7.0 6.5 6.0 5.5 5.0 4.5 4.0 3.5 3.0 2.5 2.0 1.5 1.0 0.5 0.0
f1 (ppm)

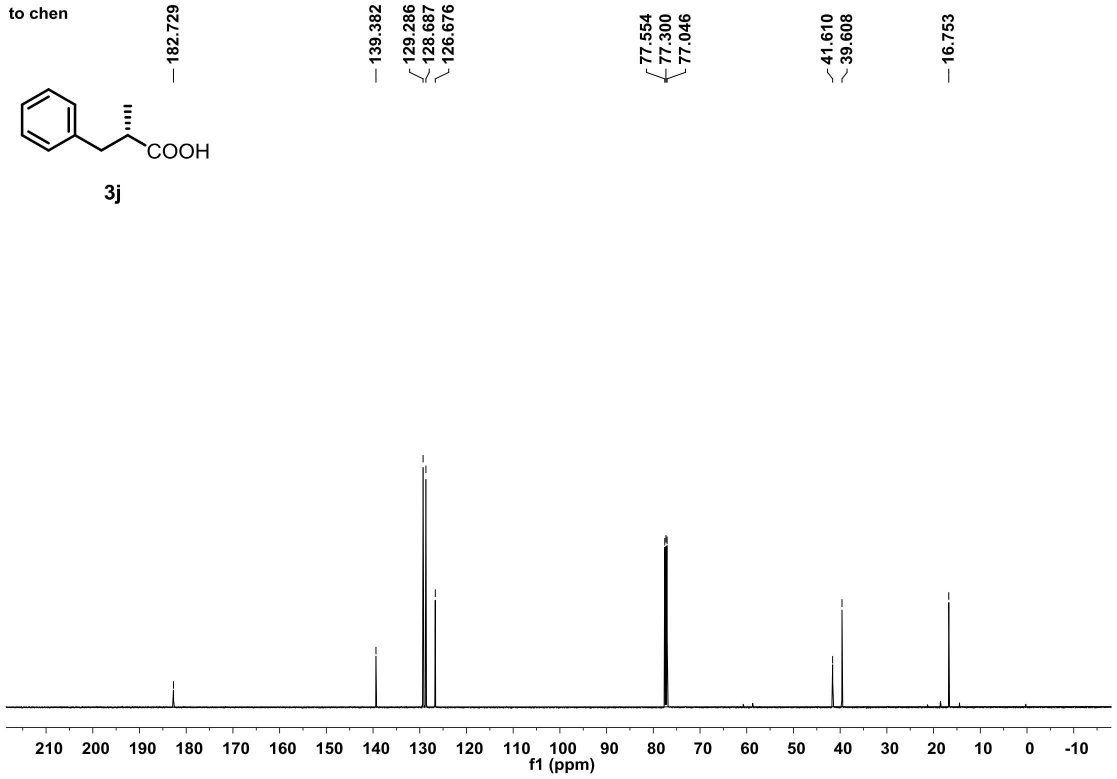
to chen
182.729
139.382
129.286
128.687
126.676
77.554
77.300
77.046
41.610
39.608
16.753
COOH
3j
210 200 190 180 170 160 150 140 130 120 110 100 90 80 70 60 50 40 30 20 10 0 -10
f1 (ppm)

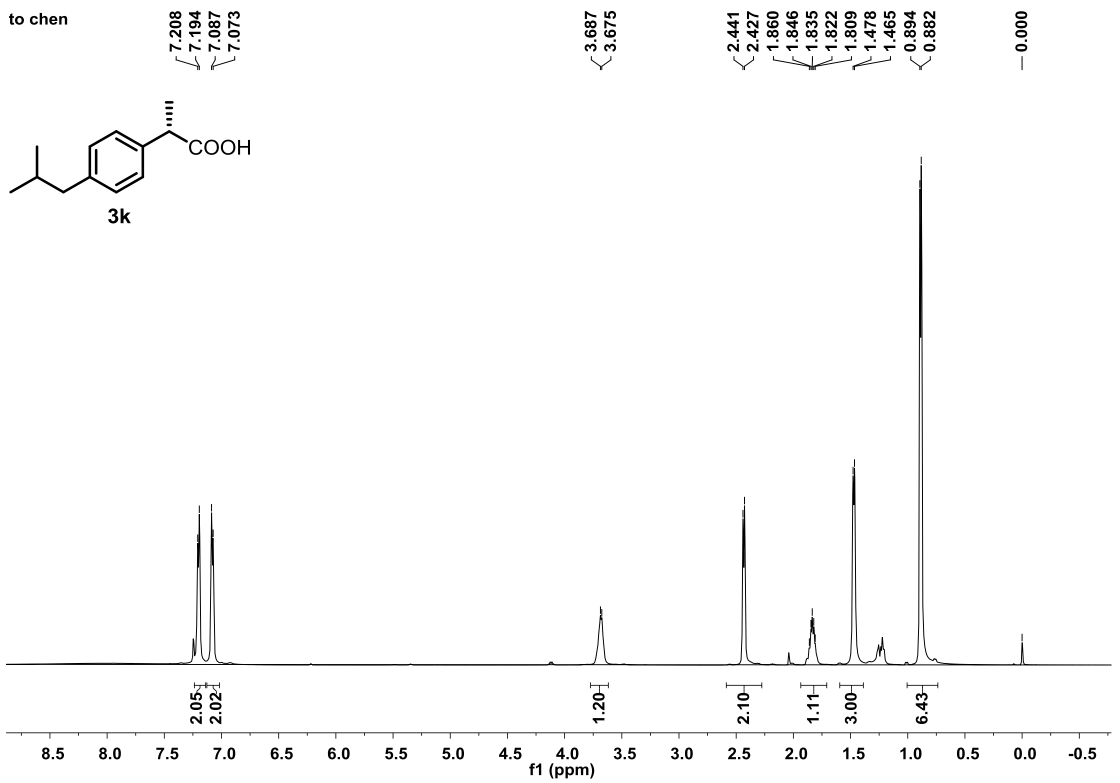
to chen
7.208
7.194
7.087
7.073
3.687
3.675
2.441
2.427
1.860
1.846
1.835
1.822
1.809
1.478
1.465
0.894
0.882
0.000
COOH
3k
2.05
2.02
1.20
2.10
1.11
3.00
6.43
8.5 8.0 7.5 7.0 6.5 6.0 5.5 5.0 4.5 4.0 3.5 3.0 2.5 2.0 1.5 1.0 0.5 0.0 -0.5
f1 (ppm)

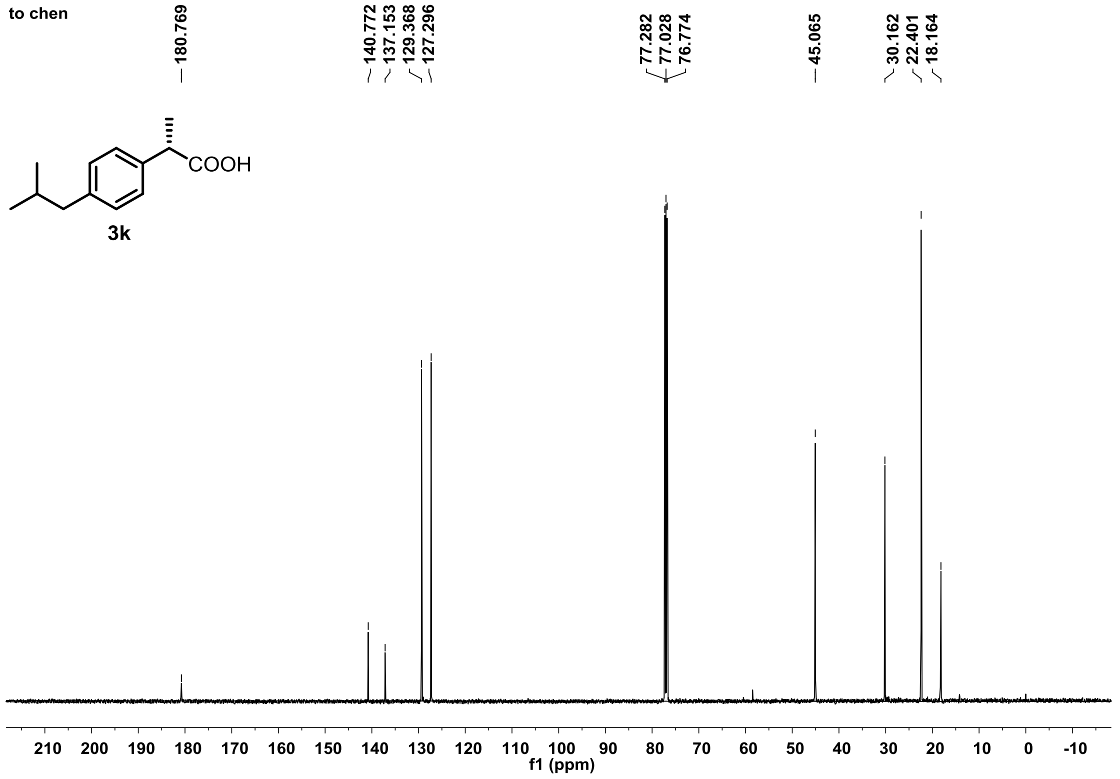
to chen
180.769
140.772
137.153
129.368
127.296
77.282
77.028
76.774
45.065
30.162
22.401
18.164
COOH
3k
210 200 190 180 170 160 150 140 130 120 110 100 90 80 70 60 50 40 30 20 10 0 -10
f1 (ppm)

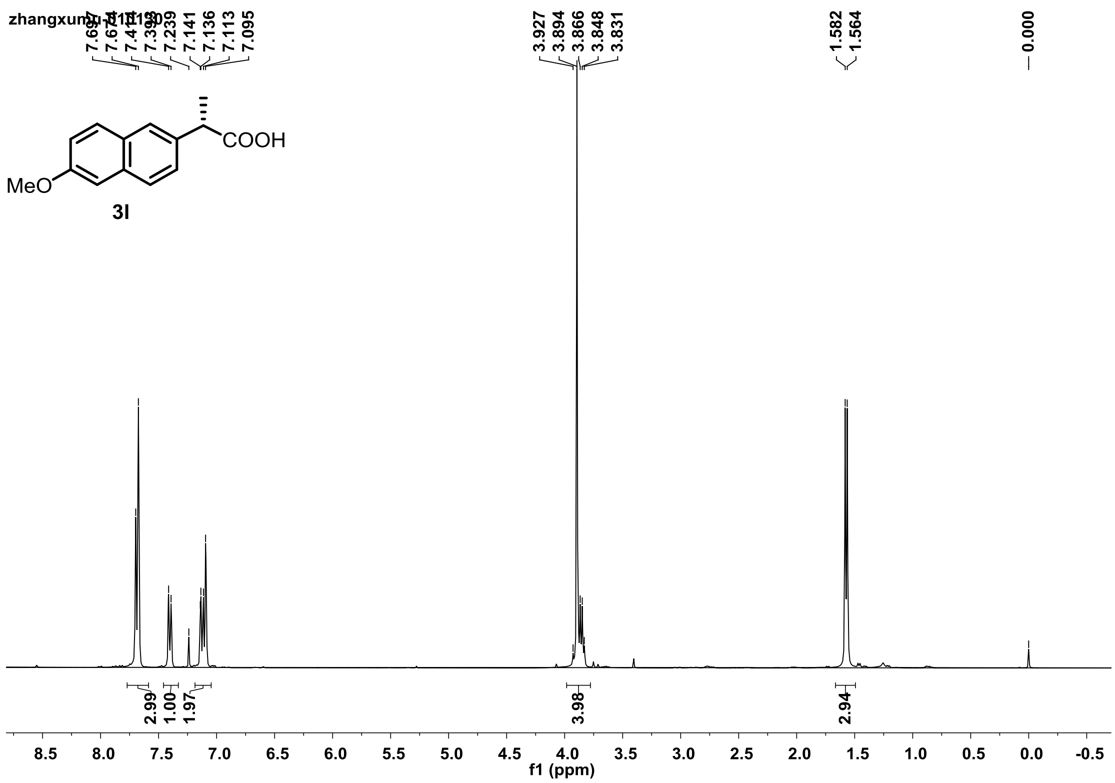
7.239
7.141
7.136
7.113
7.095
3.927
3.894
3.866
3.848
3.831
1.582
1.564
0.000
COOH
MeO
3l
2.99
1.00
1.97
3.98
2.94
8.5 8.0 7.5 7.0 6.5 6.0 5.5 5.0 4.5 4.0 3.5 3.0 2.5 2.0 1.5 1.0 0.5 0.0 -0.5
f1 (ppm)

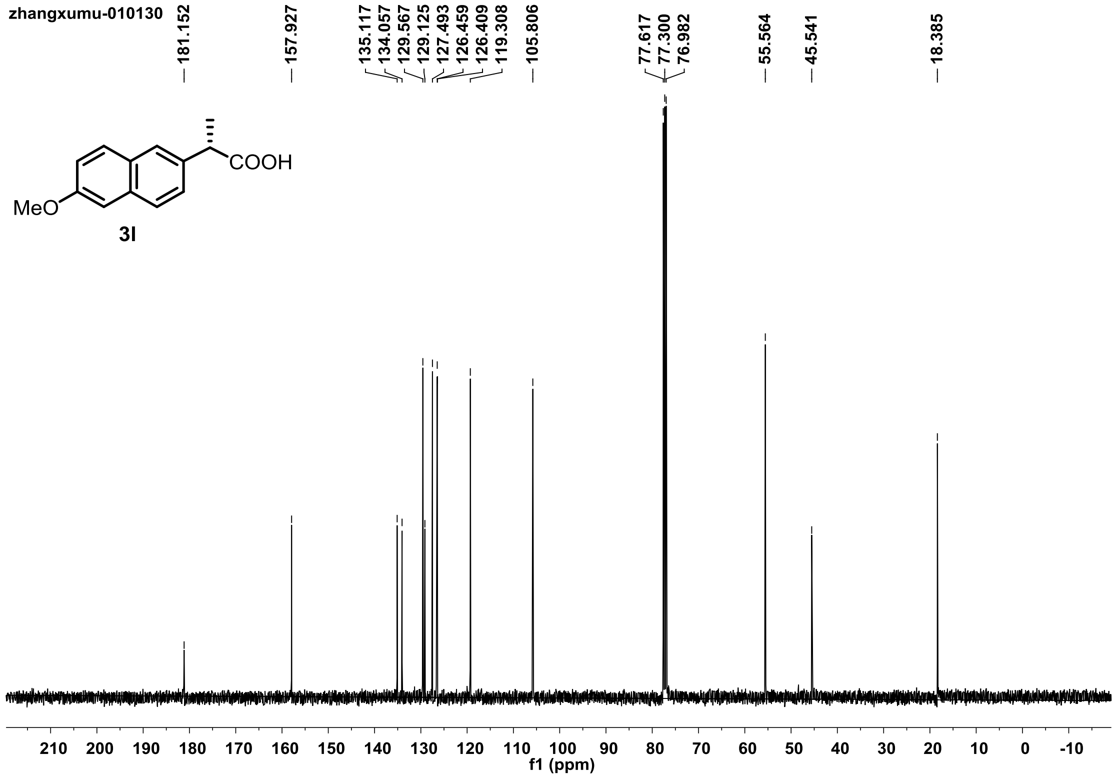
zhangxumu-010130
181.152
157.927
135.117
134.057
129.567
129.125
127.493
126.459
126.409
119.308
105.806
77.617
77.300
76.982
55.564
45.541
18.385
COOH
MeO
3l
210 200 190 180 170 160 150 140 130 120 110 100 90 80 70 60 50 40 30 20 10 0 -10
f1 (ppm)

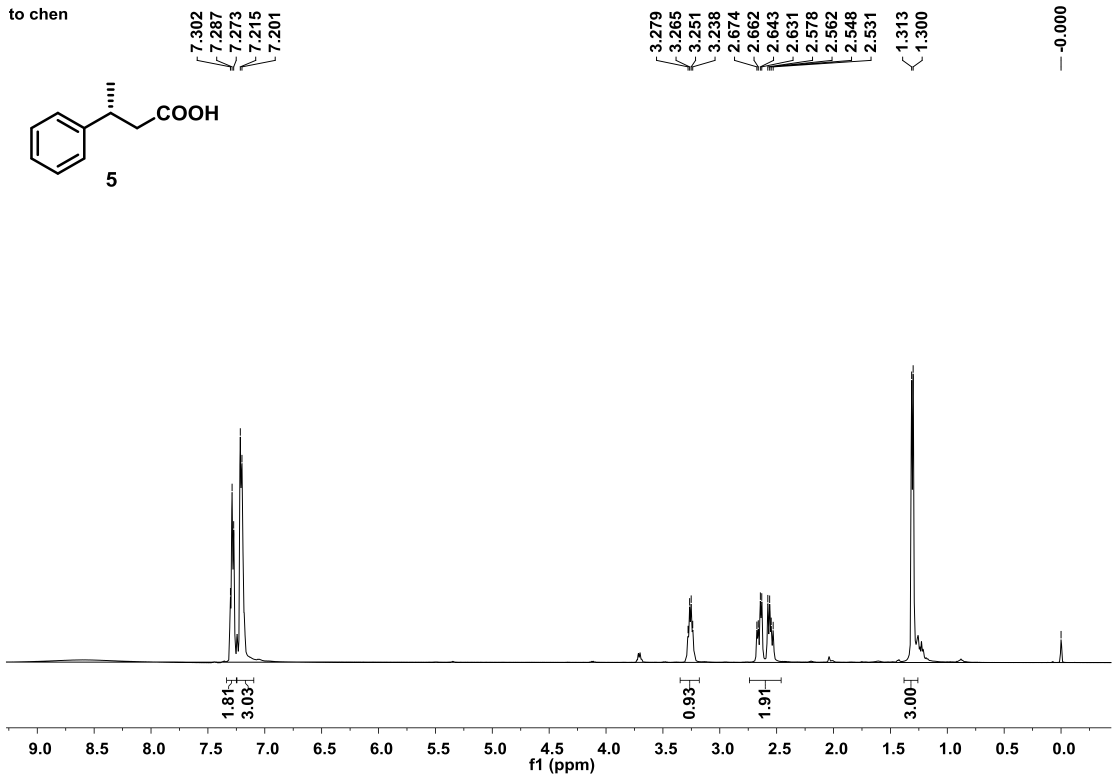
to chen
7.302
7.287
7.273
7.215
7.201
3.279
3.265
3.251
3.238
2.674
2.662
2.643
2.631
2.578
2.562
2.548
2.531
1.313
1.300
-0.000
COOH
5
1.81
3.03
0.93
1.91
3.00
f1 (ppm)

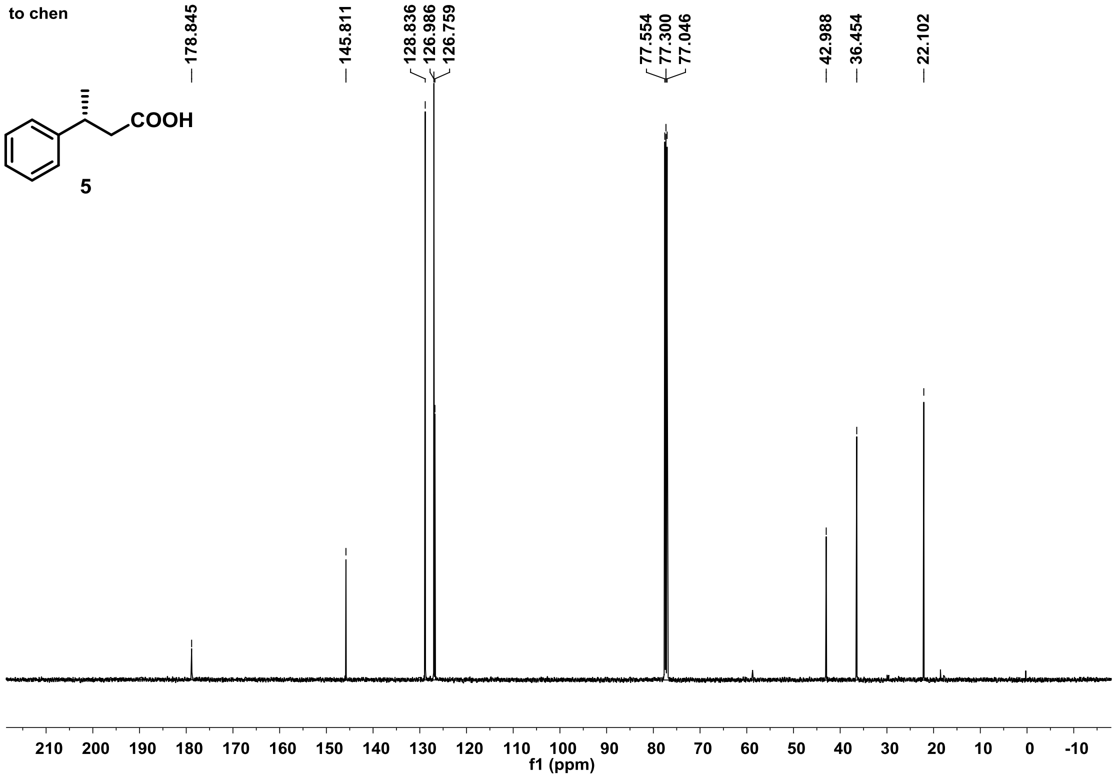
to chen
178.845
145.811
128.836
126.986
126.759
77.554
77.300
77.046
42.988
36.454
22.102
COOH
5
f1 (ppm)

HPLC and GC Spectra

Analysis Method : E:\DATA\CCY\CCY-5-197\CCY-5-197 2015-06-26 15-58-10\VWD-OJ(1-6)-99-1-1ML-205NM-20MIN.M (Sequence Method)

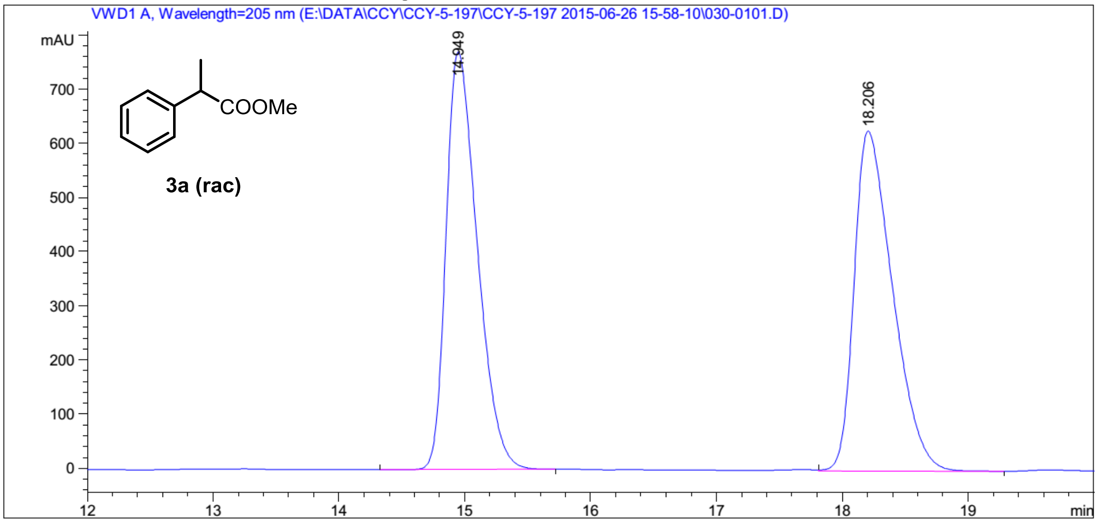

Signal 1: VWD1 A, Wavelength=205 nm

Peak #	RetTime [min]	Type	Width [min]	Area [mAU*s]	Height [mAU]	Area %
1	14.949	BB	0.2651	1.32967e4	771.43469	49.9510
2	18.206	VB	0.3237	1.33228e4	627.53394	50.0490

Totals : 2.66195e4 1398.96863

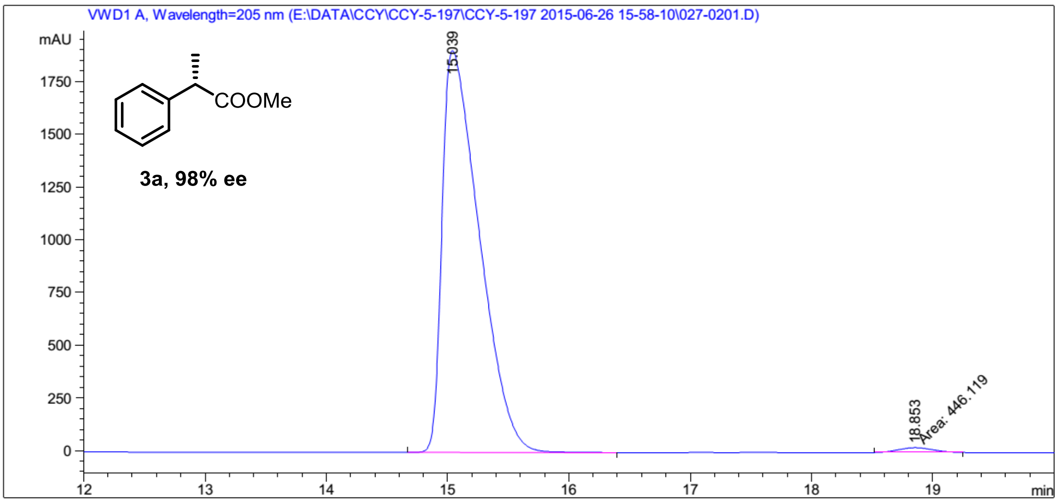

Signal 1: VWD1 A, Wavelength=205 nm

Peak #	RetTime [min]	Type	Width [min]	Area [mAU*s]	Height [mAU]	Area %
1	15.039	BB	0.3293	4.05055e4	1902.95129	98.9106
2	18.853	MM	0.3253	446.11923	22.85547	1.0894

Totals : 4.09516e4 1925.80677

Analysis Method : E:\DATA\CCY\CCY-6-20\CCY-6-20 2015-08-01 09-15-34\DAD-OJ(1-6)-99-1-1.0ML-
210-254-25MIN.M (Sequence Method)

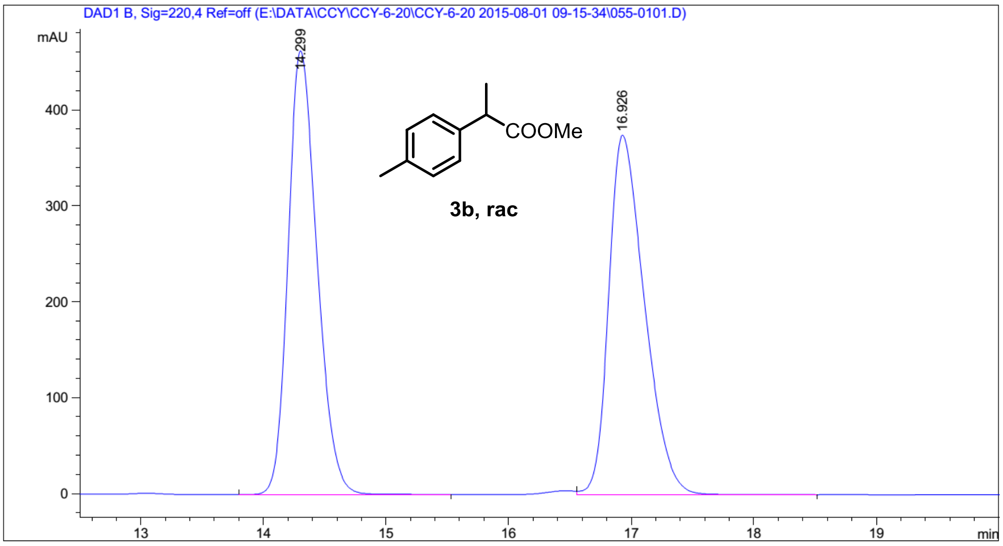

Signal 1: DAD1 B, Sig=220,4 Ref=off

Peak #	RetTime [min]	Type	Width [min]	Area [mAU*s]	Height [mAU]	Area %
1	14.299	BB	0.2530	7573.33154	462.63379	49.9295
2	16.926	VB	0.3083	7594.71289	374.90457	50.0705

Totals : 1.51680e4 837.53836

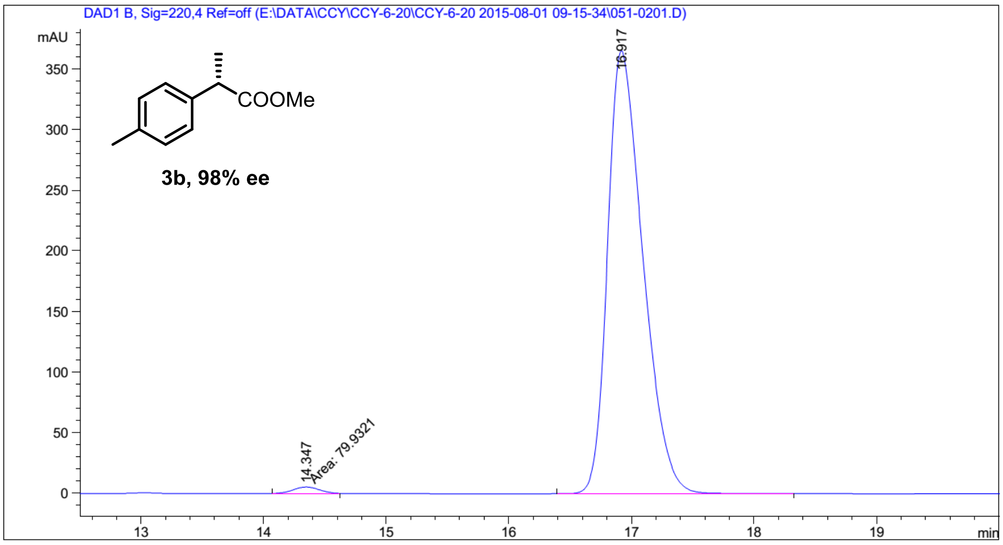

Signal 1: DAD1 B, Sig=220,4 Ref=off

Peak #	RetTime [min]	Type	Width [min]	Area [mAU*s]	Height [mAU]	Area %
1	14.347	MM	0.2497	79.93214	5.33452	1.0815
2	16.917	BB	0.3054	7310.85742	365.45529	98.9185

Totals : 7390.78956 370.78981

Analysis Method : E:\DATA\CCY\CCY-6-13\CCY-6-13A 2015-07-30 15-17-04\DAD-OJ(1-6)-99-1-1.0ML-210-254-25MIN.M (Sequence Method)

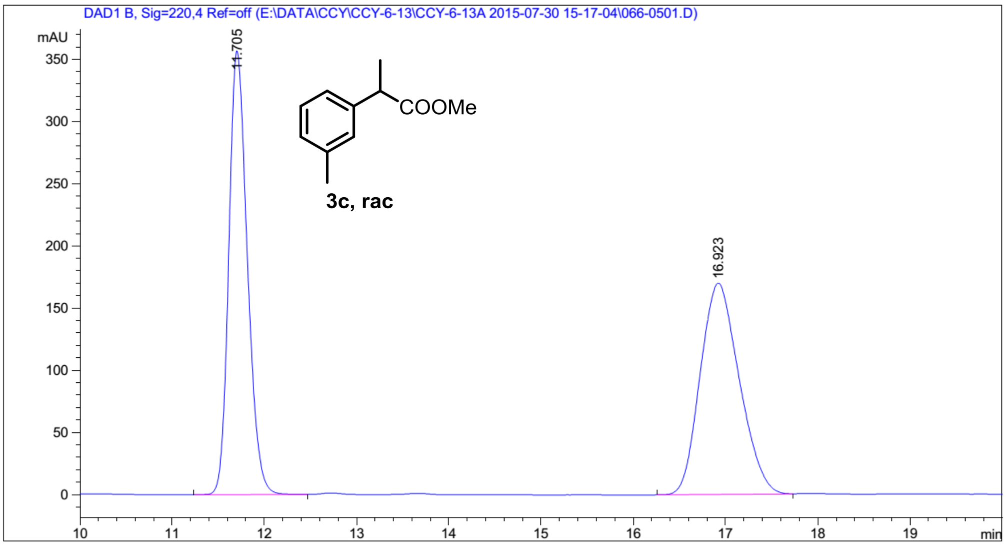

Signal 1: DAD1 B, Sig=220,4 Ref=off

Peak #	RetTime [min]	Type	Width [min]	Area [mAU*s]	Height [mAU]	Area %
1	11.705	BB	0.2149	4983.80322	356.84039	49.9957
2	16.923	BB	0.4602	4984.65430	169.92957	50.0043

Totals : 9968.45752 526.76996

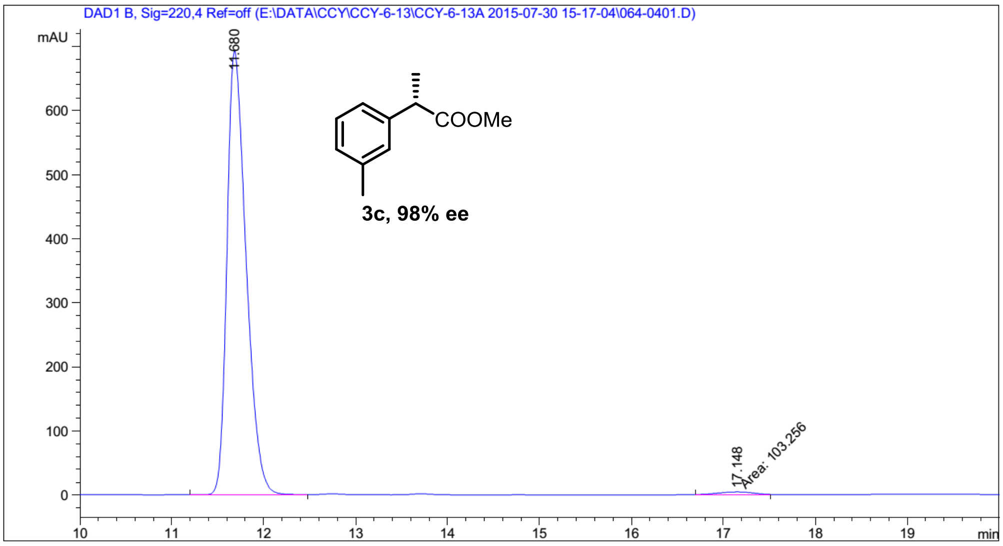

Signal 1: DAD1 B, Sig=220,4 Ref=off

Peak #	RetTime [min]	Type	Width [min]	Area [mAU*s]	Height [mAU]	Area %
1	11.680	BB	0.2206	1.00144e4	692.77075	98.9794
2	17.148	MM	0.4205	103.25599	4.09267	1.0206

Totals : 1.01177e4 696.86342

Analysis Method : E:\DATA\CCY\CCY-6-27\CCY-6-27 2015-08-06 09-06-01\DAD-OJ(1-6)-99-1-1.0ML-210-254-25MIN.M (Sequence Method)

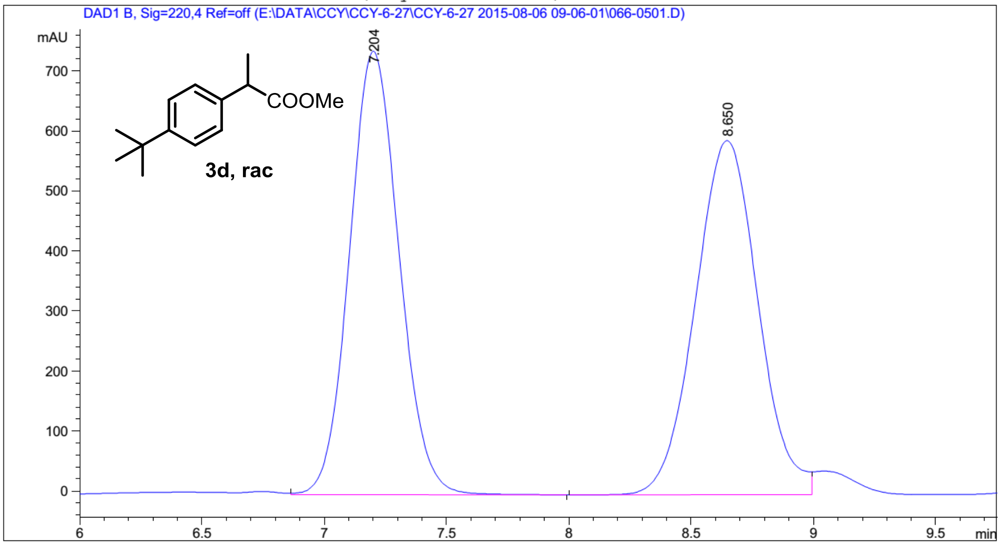

Signal 1: DAD1 B, Sig=220,4 Ref=off

Peak #	RetTime [min]	Type	Width [min]	Area [mAU*s]	Height [mAU]	Area %
1	7.204	VB	0.2182	1.04017e4	739.05127	49.7660
2	8.650	BV	0.2775	1.04995e4	590.20929	50.2340

Totals : 2.09012e4 1329.26056

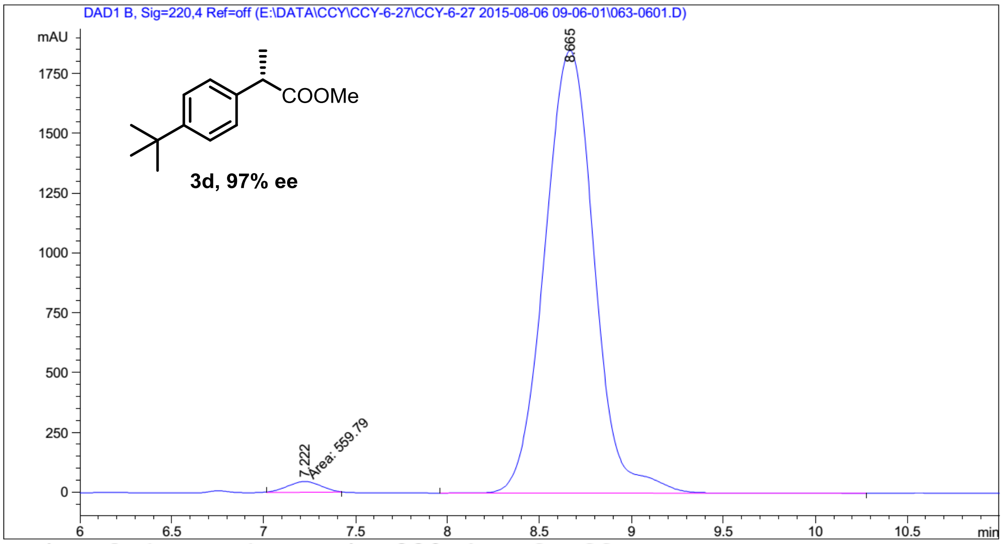

Signal 1: DAD1 B, Sig=220,4 Ref=off

Peak #	RetTime [min]	Type	Width [min]	Area [mAU*s]	Height [mAU]	Area %
1	7.222	MM	0.2103	559.78986	44.36273	1.5895
2	8.665	BB	0.2925	3.46586e4	1850.28870	98.4105

Totals : 3.52184e4 1894.65142

Analysis Method : E:\DATA\CCY\CCY-6-13\CCY-6-13A 2015-07-30 15-17-04\DAD-OJ(1-6)-99-1-1.0ML-210-254-25MIN.M (Sequence Method)

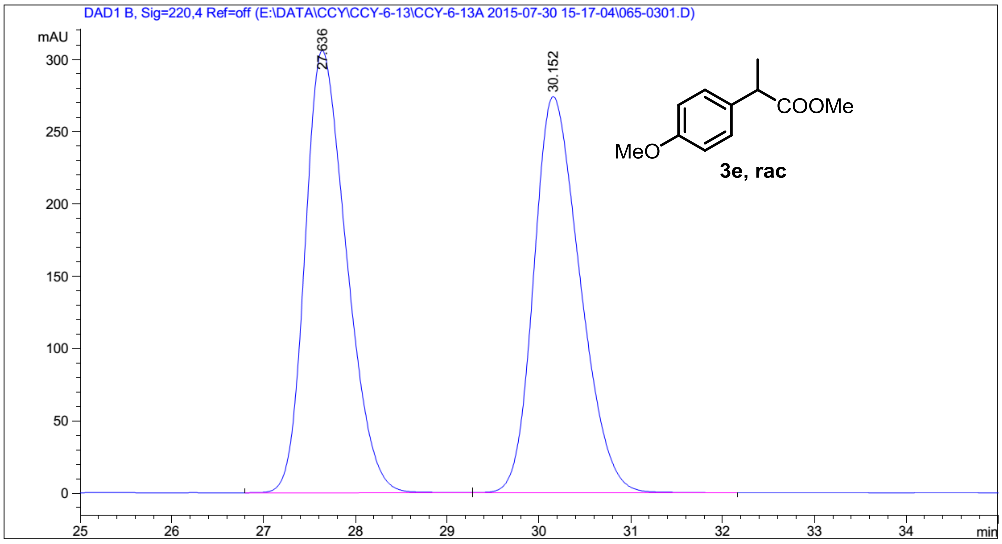

Signal 1: DAD1 B, Sig=220,4 Ref=off

Peak #	RetTime [min]	Type	Width [min]	Area [mAU*s]	Height [mAU]	Area %
1	27.636	BB	0.4834	9577.51270	305.78653	49.9969
2	30.152	BB	0.5380	9578.70313	273.88351	50.0031

Totals : 1.91562e4 579.67004

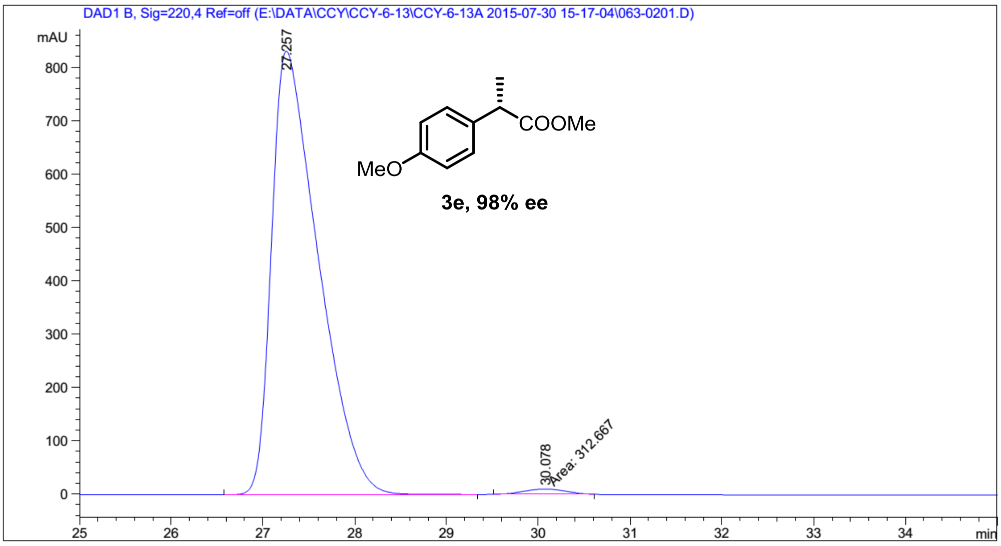

Signal 1: DAD1 B, Sig=220,4 Ref=off

Peak #	RetTime [min]	Type	Width [min]	Area [mAU*s]	Height [mAU]	Area %
1	27.257	BB	0.5282	2.93625e4	831.09302	98.9464
2	30.078	MM	0.5265	312.66672	9.89798	1.0536

Totals : 2.96752e4 840.99099

Analysis Method : E:\DATA\GM\GAOMIN20150725\GAOMIN20150725OJ 2015-07-25 16-49-07\DAD-OJ(1-6)-99-1-1.0ML-210-254-25MIN.M (Sequence Method)

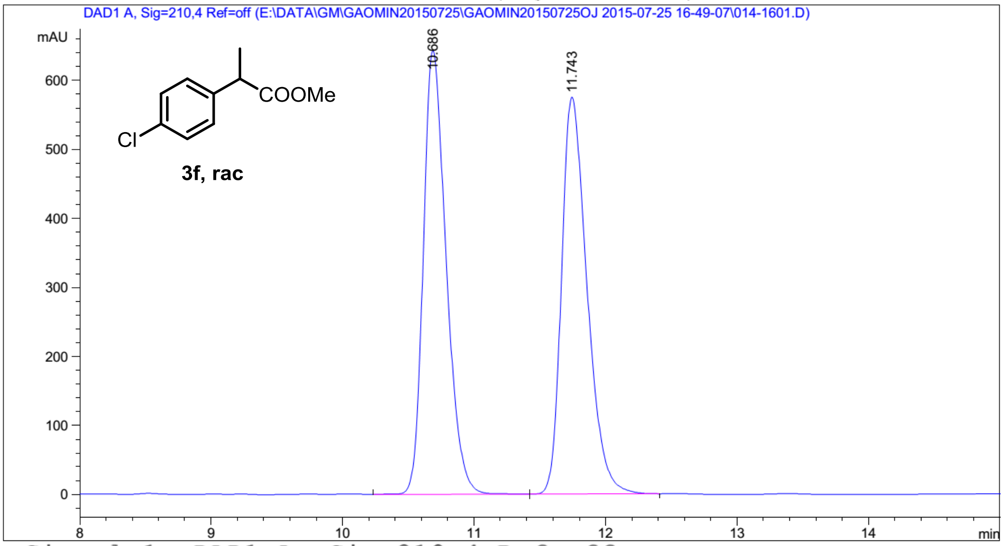

Signal 1: DAD1 A, Sig=210,4 Ref=off

Peak #	RetTime [min]	Type	Width [min]	Area [mAU*s]	Height [mAU]	Area %
1	10.686	BB	0.1847	7755.68506	642.89801	50.0583
2	11.743	BB	0.2048	7737.62402	575.71167	49.9417

Totals : 1.54933e4 1218.60968

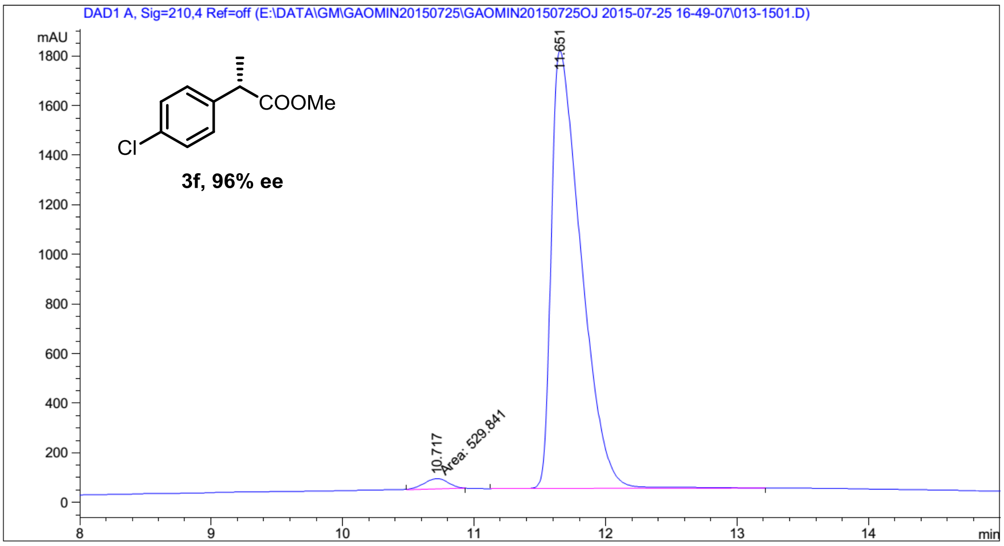

Signal 1: DAD1 A, Sig=210,4 Ref=off

Peak #	RetTime [min]	Type	Width [min]	Area [mAU*s]	Height [mAU]	Area %
1	10.717	MM	0.2130	529.84058	41.46698	1.8751
2	11.651	BB	0.2394	2.77274e4	1763.71790	98.1249

Totals : 2.82572e4 1805.18487

Analysis Method : E:\DATA\CCY\CCY-6-34\CCY-6-34 2015-08-27 19-40-29\VWD-OJ(1-6)-99-1-1.0ML-210NM-30MIN.M (Sequence Method)

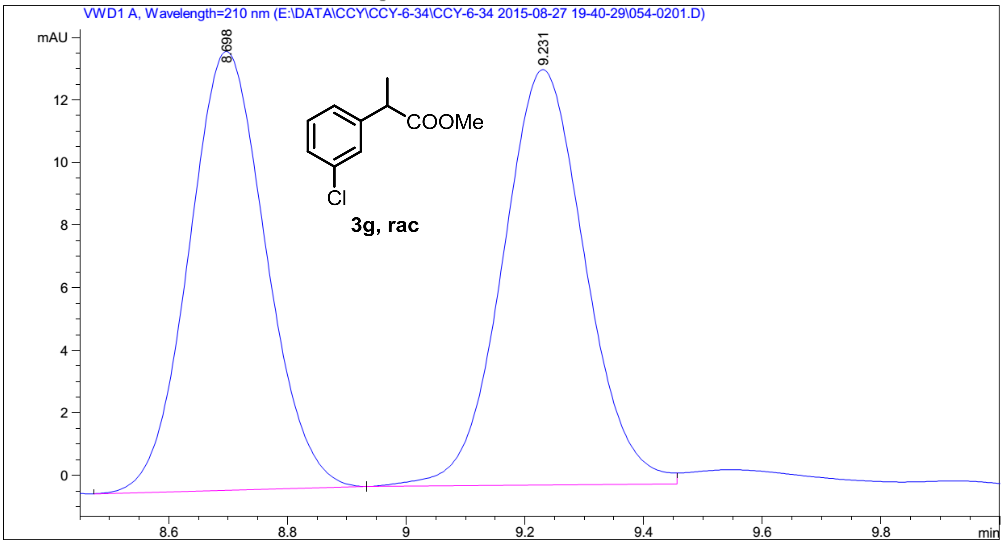

Signal 1: VWD1 A, Wavelength=210 nm

Peak #	RetTime [min]	Type	Width [min]	Area [mAU*s]	Height [mAU]	Area %
1	8.698	BB	0.1402	126.51311	14.03487	49.6177
2	9.231	BV	0.1498	128.46265	13.28364	50.3823

Totals : 254.97576 27.31851

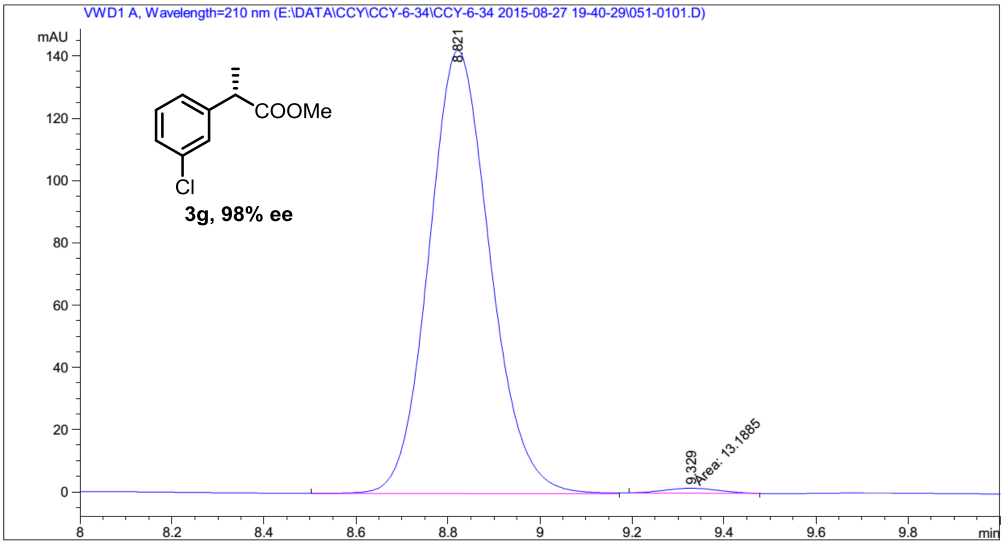

Signal 1: VWD1 A, Wavelength=210 nm

Peak #	RetTime [min]	Type	Width [min]	Area [mAU*s]	Height [mAU]	Area %
1	8.821	BV	0.1421	1319.13843	142.33096	99.0101
2	9.329	MM	0.1401	13.18855	1.56934	0.9899

Totals : 1332.32698 143.90031

Analysis Method : E:\DATA\CCY\CCY-6-27\CCY-6-27 2015-08-06 09-06-01\DAD-OJ(1-6)-99-1-1.0ML-210-254-25MIN.M (Sequence Method)

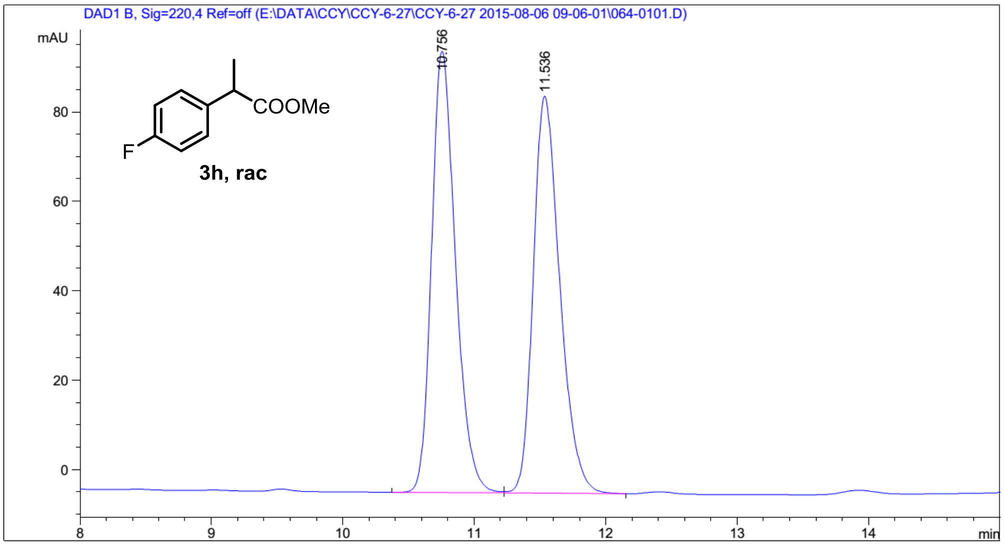

Signal 1: DAD1 B, Sig=220,4 Ref=off

Peak #	RetTime [min]	Type	Width [min]	Area [mAU*s]	Height [mAU]	Area %
1	10.756	BV	0.1937	1249.49084	98.69353	50.0773
2	11.536	VB	0.2156	1245.63208	88.82416	49.9227

Totals : 2495.12292 187.51769

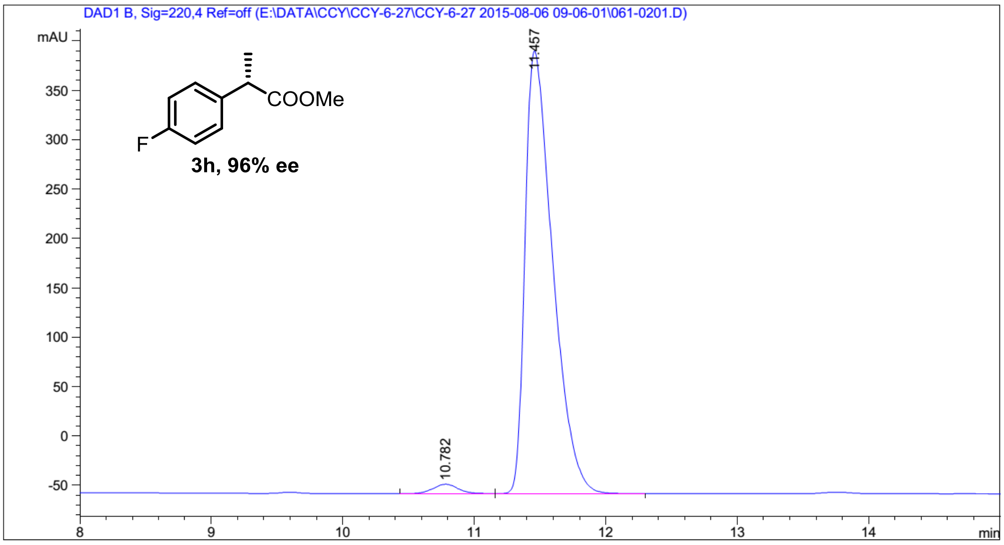

Signal 1: DAD1 B, Sig=220,4 Ref=off

Peak #	RetTime [min]	Type	Width [min]	Area [mAU*s]	Height [mAU]	Area %
1	10.782	BB	0.1985	127.03754	9.59172	1.9083
2	11.457	BB	0.2219	6530.15576	448.41965	98.0917

Totals : 6657.19330 458.01137

<Sample Information>

Sample Name : ccy-5-205-7
Sample ID :
Data Filename : ccy-5-205-7.gcd
Method Filename : b-dex225-250-80-220-260-70min.gcm

<Chromatogram>

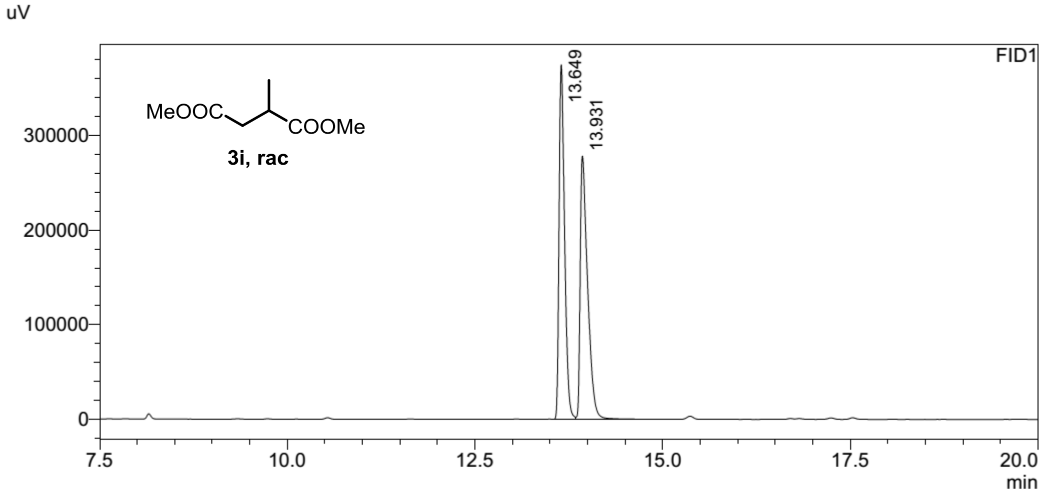

<Peak Table>

FID1

Peak#	Ret. Time	Area	Height	Conc.	Unit	Mark	Name
1	13.649	1917091	374530	50.065			
2	13.931	1912150	277825	49.935		SV	
Total		3829241	652354				

<Sample Information>

Sample Name : ccy-5-205-4
Sample ID :
Data Filename : ccy-5-205-4.gcd
Method Filename : b-dex225-250-80-220-260-70min.gcm

<Chromatogram>

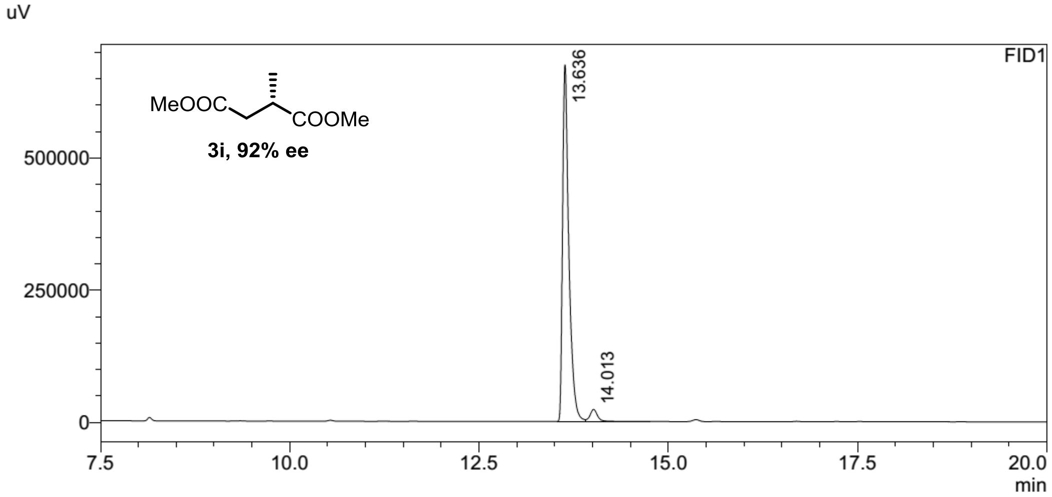

<Peak Table>

FID1

Peak#	Ret. Time	Area	Height	Conc.	Unit	Mark	Name
1	13.636	3894209	673999	95.903		V	
2	14.013	166372	23066	4.097		SV	
Total		4060580	697065				

Analysis Method : E:\DATA\CCY\CCY-5-204-205\DX20150628-ZXB-33-34-VWD-OJ(1-6)-95-5 2015-07-07 09-41-18\VWD-OJ(1-6)-99-1-1ML-205NM-20MIN.M (Sequence Method)

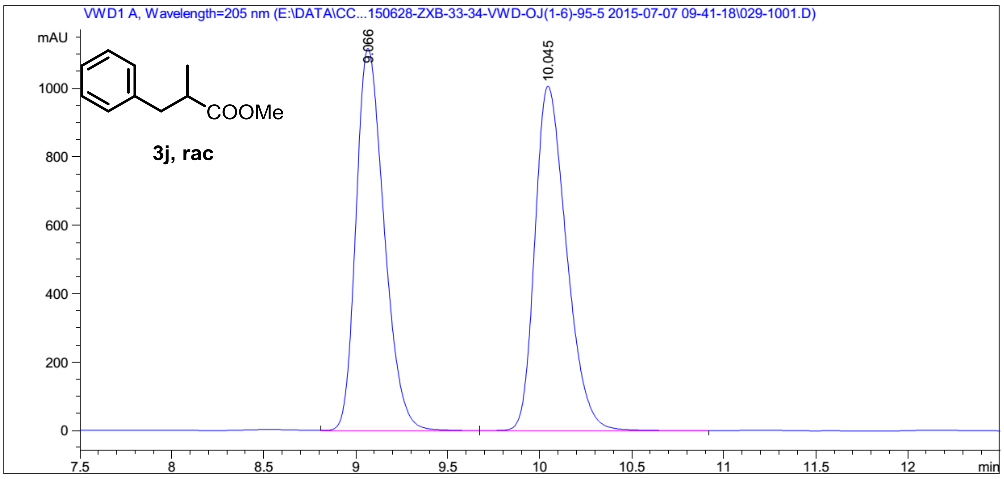

Signal 1: VWD1 A, Wavelength=205 nm

Peak #	RetTime [min]	Type	Width [min]	Area [mAU*s]	Height [mAU]	Area %
1	9.066	VB	0.1639	1.18696e4	1117.72522	49.6954
2	10.045	BB	0.1841	1.20151e4	1008.02130	50.3046

Totals : 2.38846e4 2125.74652

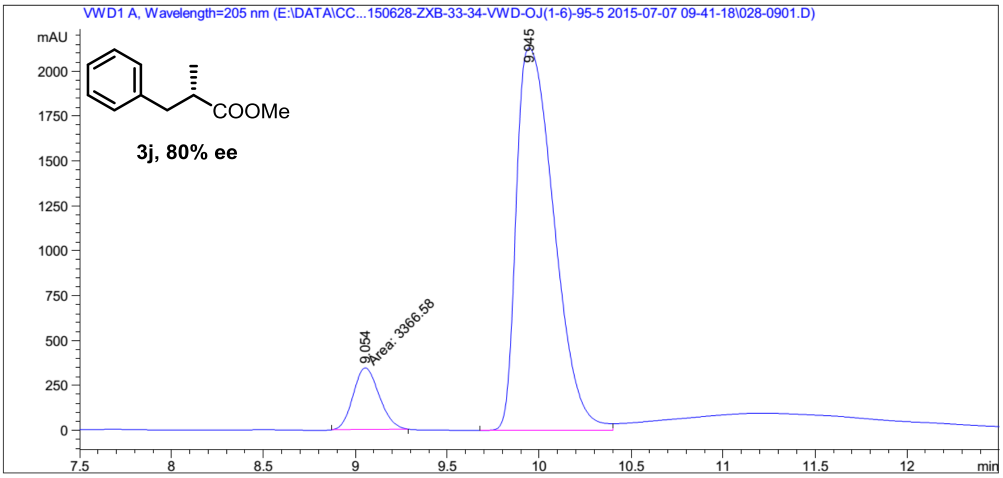

Signal 1: VWD1 A, Wavelength=205 nm

Peak #	RetTime [min]	Type	Width [min]	Area [mAU*s]	Height [mAU]	Area %
1	9.054	MM	0.1635	3366.58398	343.21301	9.9904
2	9.945	BV	0.2251	3.03318e4	2130.07104	90.0096

Totals : 3.36984e4 2473.28406

Analysis Method : E:\DATA\GM\GAOMIN20150725\GAOMIN20150725OJ 2015-07-25 16-49-07\DAD-OJ(1-6)-99-1-1.0ML-210-254-25MIN.M (Sequence Method)

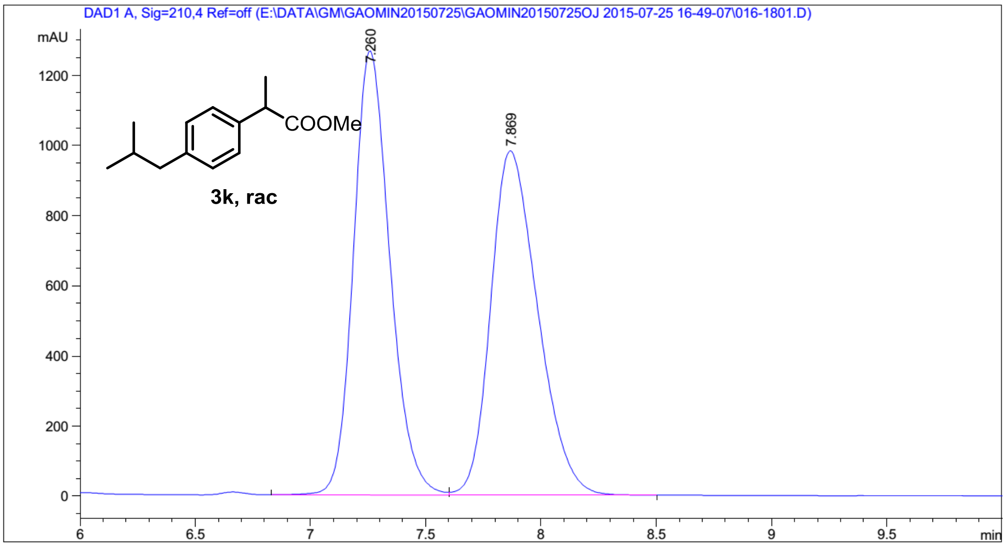

Signal 1: DAD1 A, Sig=210,4 Ref=off

Peak #	RetTime [min]	Type	Width [min]	Area [mAU*s]	Height [mAU]	Area %
1	7.260	BV	0.1703	1.39336e4	1266.96326	50.0365
2	7.869	VB	0.2193	1.39132e4	982.13269	49.9635

Totals : 2.78468e4 2249.09595

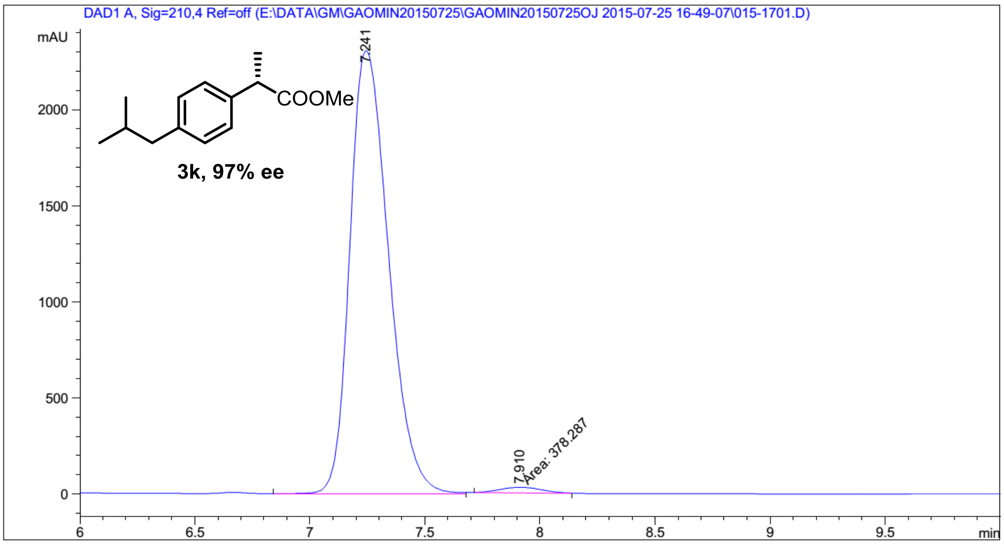

Signal 1: DAD1 A, Sig=210,4 Ref=off

Peak #	RetTime [min]	Type	Width [min]	Area [mAU*s]	Height [mAU]	Area %
1	7.241	BV	0.1804	2.69508e4	2304.97705	98.6158
2	7.910	MM	0.2144	378.28711	29.40901	1.3842

Totals : 2.73291e4 2334.38606

Analysis Method : E:\DATA\CCY\CCY-6-34\CCY-6-34 2015-08-27 19-40-29\VWD-OJ(1-6)-90-10-1.0ML-210NM-40MIN.M (Sequence Method)

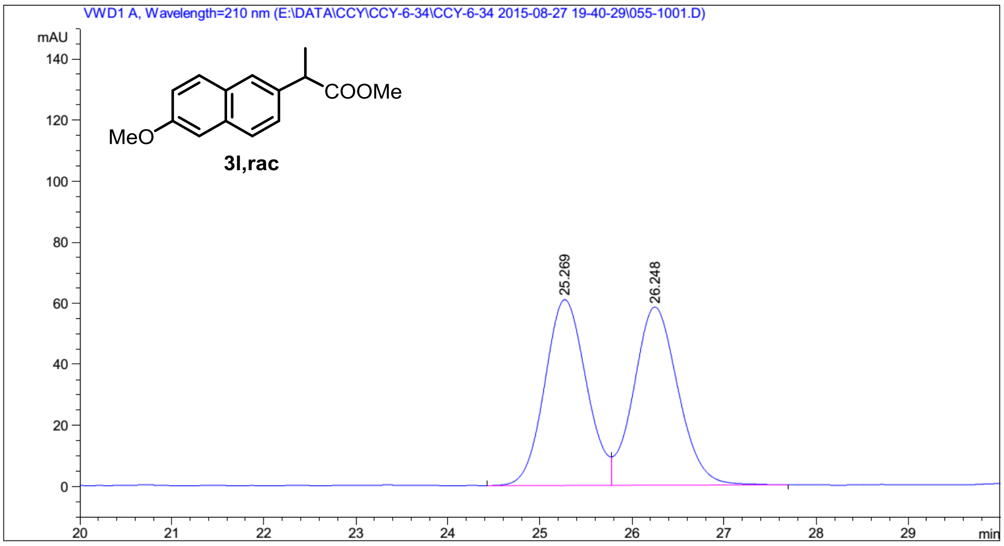

Signal 1: VWD1 A, Wavelength=210 nm

Peak #	RetTime [min]	Type	Width [min]	Area [mAU*s]	Height [mAU]	Area %
1	25.269	BV	0.4861	1909.86865	60.86845	49.4233
2	26.248	VB	0.5143	1954.44250	58.41681	50.5767

Totals : 3864.31116 119.28525

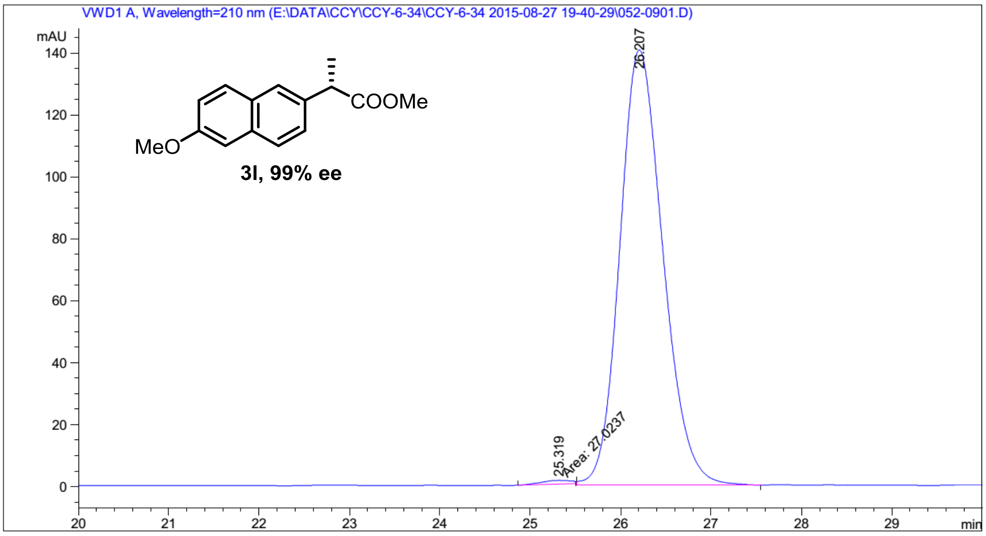

Signal 1: VWD1 A, Wavelength=210 nm

Peak #	RetTime [min]	Type	Width [min]	Area [mAU*s]	Height [mAU]	Area %
1	25.319	MM	0.3943	27.02372	1.14234	0.5766
2	26.207	VB	0.5129	4659.44189	140.49760	99.4234

Totals : 4686.46562 141.63995

<Sample Information>

Sample Name : ccy-6-72-2
Sample ID :
Data Filename : ccy-6-72-2.gcd
Method Filename : CB-230-6=90-190-240-30min.gcm

<Chromatogram>

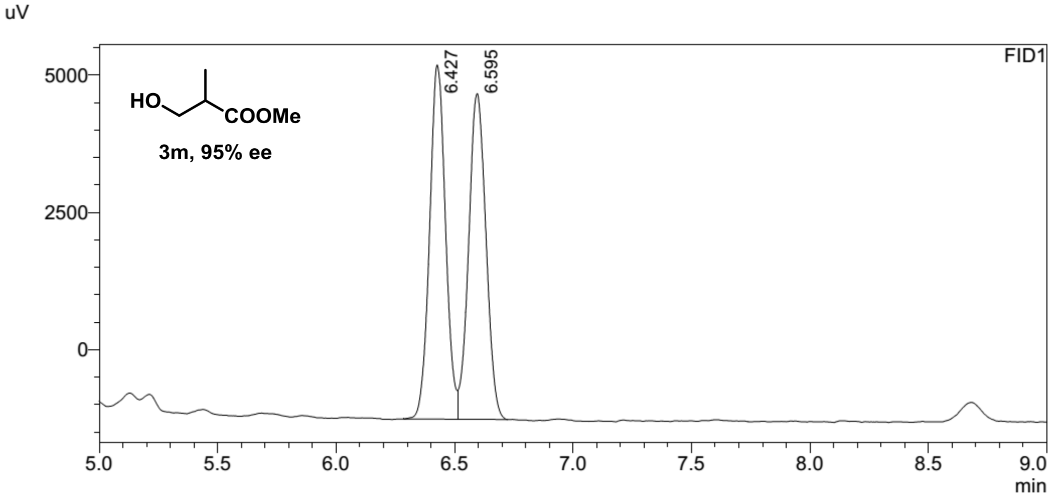

<Peak Table>

FID1

Peak#	Ret. Time	Area	Height	Conc.	Unit	Mark	Name
1	6.427	30264	6416	50.201			
2	6.595	30022	5916	49.799		V	
Total		60286	12331				

<Sample Information>

Sample Name : ccy-6-72-1
Sample ID :
Data Filename : ccy-6-72-1.gcd
Method Filename : CB-230-6=90-190-240-30min.gcm

<Chromatogram>

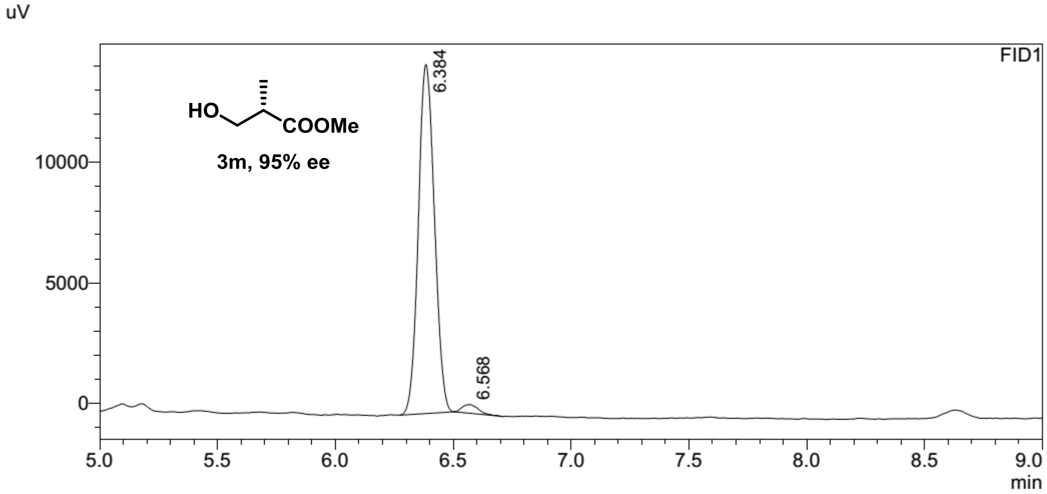

<Peak Table>

FID1

Peak#	Ret. Time	Area	Height	Conc.	Unit	Mark	Name
1	6.384	68848	14458	97.609			
2	6.568	1687	356	2.391		M	
Total		70534	14814				

References

1. (*a*) S. Ghosh, S. N. Pardo, R. G. Salomon, *J. Org. Chem.*, **1982**, *47*, 4693; (*b*) F. X. Felpin, K. Miqueu, J. M. Sotiropoulos, E. Fouquet, O. Ibarguren, J. Laudien, *Chem. Eur. J.*, **2010**, *16*, 5191.
2. R. R. Kurtz, D. J. Houser, *J. Org. Chem.*, **1981**, *46*, 202.
3. F. Bellezza, A. Cipiciani, G. Ricci, R. Ruzziconi, *Tetrahedron*, **2005**, *61*, 8005
4. T. Mendgen, T. Scholz, C. D. Klein, *Bioorg. Med. Chem. Lett.* **2010**, *20*, 5757
5. A. Takemiya, J. F. Hartwig, J. *Am. Chem. Soc.*, **2006**, *128*, 6042.
6. X. Sun, L. Zhou, C.-J. Wang, X. Zhang, *Angew. Chem. Int. Ed.*, 2007, **46**, 2623.
7. S.-F. Zhu, Y.-B. Yu, S. Li, L.-X. Wang, Q.-L. Zhou, *Angew. Chem. Int. Ed.*, 2012, **51**, 8872.
8. E. Boyd, E. Coulbeck, G. S. Coumbarides, S. Chavda, M. Dingjan, J. Eames, A. Flinn, M. Motevalli, J. Northend, Y. Yohannes, *Tetrahedron: Asymmetr*y, 2007, **18**, 2515.
9. Z.-L. Wu, Z.-Y. Li, *Tetrahedron: Asymmetry*, 2001, **12**, 3305.
10. O. Hiromichi, *Jpn. Kokai Tokkyo Koho*, 1993, JP 05176778 A 19930720.
11. O. Piccolo, F. Spreafico, G. Visentin, *J. Org. Chem.*, 1987, **52**, 10.
12. C. E. Stivala, A. Zakarian, *J. Am. Chem. Soc*, **2011**, *133*, 11936.
13. P. C. B. Page, M. J. McKenzie, S. M. Allin, S. S. Klair, *Tetrahedron*. **1997**, *53*, 13149.
14. S. Wang, Z. Wang, L. Yang, J. Dong, C. Chi, D. Sui, Y. Wang, J. Ren, M. Hung, Y. Jiang, *Journal of Molecular Catalysis A: Chemical*, **2007**, *264*, 60.
